# Supplementary material for: AssemblyTron: flexible automation of DNA assembly with Opentrons OT-2 lab robots
Source: Synth Biol (Oxf). 2022 Dec 22;8(1):ysac032. doi: 10.1093/synbio/ysac032 (PMC9832943; doi:10.1093/synbio/ysac032)
Supplement: ysac032_Supp [file ysac032_supp.zip › suppl_data/File5_rxn1.rtf]

Mon Sep 12, 2022 11:01 EDT-ARF5-pdar-pmas00001.gb from 1 to 9308to_B10_IVA_rxn1_C5+206_TCYC1_Seq_R.ab1--  Matches:1277; Mismatches:5; Gaps:8181; Unattempted:0_C10_IVA_rxn1_C6+206_TCYC1_Seq_R.ab1--  Matches:1130; Mismatches:2; Gaps:3635; Unattempted:4687_A10_IVA_rxn1_C4+206_TCYC1_Seq_R.ab1--  Matches:1127; Mismatches:2; Gaps:3632; Unattempted:4693_E03_IVA_rxn1_C3+206_TCYC1_Seq_R.ab1--  Matches:1106; Mismatches:3; Gaps:3641; Unattempted:4708_C03_IVA_rxn1_C1+206_TCYC1_Seq_R.ab1--  Matches:1012; Mismatches:0; Gaps:3688; Unattempted:4810_D03_IVA_rxn1_C2+206_TCYC1_Seq_R.ab1--  Matches:999; Mismatches:4; Gaps:3685; Unattempted:4823                *         *         *         *         *         *         *         *         *         *     1>tcgcgcgtttcggtgatgacggtgaaaacctctgacacatgcagctcccggagacggtcacagcttgtctgtaagcggatgccgggagcagacaagcccg>100      1438<~~~~~~~~~~~~~~~~~~~~~~~~~~~~~~~~~~~~~~~~~~~~~~~~~~~~~~~~~~~~~~~~~~~~~~~~~~~~~~~~~~~~~~~~~~~~~~~~~~~~<1438     1468<~~~~~~~~~~~~~~~~~~~~~~~~~~~~~~~~~~~~~~~~~~~~~~~~~~~~~~~~~~~~~~~~~~~~~~~~~~~~~~~~~~~~~~~~~~~~~~~~~~~~<1468     1464<~~~~~~~~~~~~~~~~~~~~~~~~~~~~~~~~~~~~~~~~~~~~~~~~~~~~~~~~~~~~~~~~~~~~~~~~~~~~~~~~~~~~~~~~~~~~~~~~~~~~<1464     1407<~~~~~~~~~~~~~~~~~~~~~~~~~~~~~~~~~~~~~~~~~~~~~~~~~~~~~~~~~~~~~~~~~~~~~~~~~~~~~~~~~~~~~~~~~~~~~~~~~~~~<1407     1379<~~~~~~~~~~~~~~~~~~~~~~~~~~~~~~~~~~~~~~~~~~~~~~~~~~~~~~~~~~~~~~~~~~~~~~~~~~~~~~~~~~~~~~~~~~~~~~~~~~~~<1379     1410<~~~~~~~~~~~~~~~~~~~~~~~~~~~~~~~~~~~~~~~~~~~~~~~~~~~~~~~~~~~~~~~~~~~~~~~~~~~~~~~~~~~~~~~~~~~~~~~~~~~~<1410                   *         *         *         *         *         *         *         *         *         *   101>tcagggcgcgtcagcgggtgttggcgggtgtcggggctggcttaactatgcggcatcagagcagattgtactgagagtgcaccataattcgtttaaaccg>200      1438<~~~~~~~~~~~~~~~~~~~~~~~~~~~~~~~~~~~~~~~~~~~~~~~~~~~~~~~~~~~~~~~~~~~~~~~~~~~~~~~~~~~~~~~~~~~~~~~~~~~~<1438     1468<~~~~~~~~~~~~~~~~~~~~~~~~~~~~~~~~~~~~~~~~~~~~~~~~~~~~~~~~~~~~~~~~~~~~~~~~~~~~~~~~~~~~~~~~~~~~~~~~~~~~<1468     1464<~~~~~~~~~~~~~~~~~~~~~~~~~~~~~~~~~~~~~~~~~~~~~~~~~~~~~~~~~~~~~~~~~~~~~~~~~~~~~~~~~~~~~~~~~~~~~~~~~~~~<1464     1407<~~~~~~~~~~~~~~~~~~~~~~~~~~~~~~~~~~~~~~~~~~~~~~~~~~~~~~~~~~~~~~~~~~~~~~~~~~~~~~~~~~~~~~~~~~~~~~~~~~~~<1407     1379<~~~~~~~~~~~~~~~~~~~~~~~~~~~~~~~~~~~~~~~~~~~~~~~~~~~~~~~~~~~~~~~~~~~~~~~~~~~~~~~~~~~~~~~~~~~~~~~~~~~~<1379     1410<~~~~~~~~~~~~~~~~~~~~~~~~~~~~~~~~~~~~~~~~~~~~~~~~~~~~~~~~~~~~~~~~~~~~~~~~~~~~~~~~~~~~~~~~~~~~~~~~~~~~<1410                   *         *         *         *         *         *         *         *         *         *   201>ttttaagagcttggtgagcgctaggagtcactgccaggtatcgtttgaacacggcattagtcagggaagtcataacacagtcctttcccgcaattttctt>300      1438<~~~~~~~~~~~~~~~~~~~~~~~~~~~~~~~~~~~~~~~~~~~~~~~~~~~~~~~~~~~~~~~~~~~~~~~~~~~~~~~~~~~~~~~~~~~~~~~~~~~~<1438     1468<~~~~~~~~~~~~~~~~~~~~~~~~~~~~~~~~~~~~~~~~~~~~~~~~~~~~~~~~~~~~~~~~~~~~~~~~~~~~~~~~~~~~~~~~~~~~~~~~~~~~<1468     1464<~~~~~~~~~~~~~~~~~~~~~~~~~~~~~~~~~~~~~~~~~~~~~~~~~~~~~~~~~~~~~~~~~~~~~~~~~~~~~~~~~~~~~~~~~~~~~~~~~~~~<1464     1407<~~~~~~~~~~~~~~~~~~~~~~~~~~~~~~~~~~~~~~~~~~~~~~~~~~~~~~~~~~~~~~~~~~~~~~~~~~~~~~~~~~~~~~~~~~~~~~~~~~~~<1407     1379<~~~~~~~~~~~~~~~~~~~~~~~~~~~~~~~~~~~~~~~~~~~~~~~~~~~~~~~~~~~~~~~~~~~~~~~~~~~~~~~~~~~~~~~~~~~~~~~~~~~~<1379     1410<~~~~~~~~~~~~~~~~~~~~~~~~~~~~~~~~~~~~~~~~~~~~~~~~~~~~~~~~~~~~~~~~~~~~~~~~~~~~~~~~~~~~~~~~~~~~~~~~~~~~<1410                   *         *         *         *         *         *         *         *         *         *   301>tttctattactcttggcctcctctagtacactctatatttttttatgcctcggtaatgattttcatttttttttttccacctagcggatgactctttttt>400      1438<~~~~~~~~~~~~~~~~~~~~~~~~~~~~~~~~~~~~~~~~~~~~~~~~~~~~~~~~~~~~~~~~~~~~~~~~~~~~~~~~~~~~~~~~~~~~~~~~~~~~<1438     1468<~~~~~~~~~~~~~~~~~~~~~~~~~~~~~~~~~~~~~~~~~~~~~~~~~~~~~~~~~~~~~~~~~~~~~~~~~~~~~~~~~~~~~~~~~~~~~~~~~~~~<1468     1464<~~~~~~~~~~~~~~~~~~~~~~~~~~~~~~~~~~~~~~~~~~~~~~~~~~~~~~~~~~~~~~~~~~~~~~~~~~~~~~~~~~~~~~~~~~~~~~~~~~~~<1464     1407<~~~~~~~~~~~~~~~~~~~~~~~~~~~~~~~~~~~~~~~~~~~~~~~~~~~~~~~~~~~~~~~~~~~~~~~~~~~~~~~~~~~~~~~~~~~~~~~~~~~~<1407     1379<~~~~~~~~~~~~~~~~~~~~~~~~~~~~~~~~~~~~~~~~~~~~~~~~~~~~~~~~~~~~~~~~~~~~~~~~~~~~~~~~~~~~~~~~~~~~~~~~~~~~<1379     1410<~~~~~~~~~~~~~~~~~~~~~~~~~~~~~~~~~~~~~~~~~~~~~~~~~~~~~~~~~~~~~~~~~~~~~~~~~~~~~~~~~~~~~~~~~~~~~~~~~~~~<1410                   *         *         *         *         *         *         *         *         *         *   401>tttcttagcgattggcattatcacataatgaattatacattatataaagtaatgtgatttcttcgaagaatatactaaaaaatgagcaggcaagataaac>500      1438<~~~~~~~~~~~~~~~~~~~~~~~~~~~~~~~~~~~~~~~~~~~~~~~~~~~~~~~~~~~~~~~~~~~~~~~~~~~~~~~~~~~~~~~~~~~~~~~~~~~~<1438     1468<~~~~~~~~~~~~~~~~~~~~~~~~~~~~~~~~~~~~~~~~~~~~~~~~~~~~~~~~~~~~~~~~~~~~~~~~~~~~~~~~~~~~~~~~~~~~~~~~~~~~<1468     1464<~~~~~~~~~~~~~~~~~~~~~~~~~~~~~~~~~~~~~~~~~~~~~~~~~~~~~~~~~~~~~~~~~~~~~~~~~~~~~~~~~~~~~~~~~~~~~~~~~~~~<1464     1407<~~~~~~~~~~~~~~~~~~~~~~~~~~~~~~~~~~~~~~~~~~~~~~~~~~~~~~~~~~~~~~~~~~~~~~~~~~~~~~~~~~~~~~~~~~~~~~~~~~~~<1407     1379<~~~~~~~~~~~~~~~~~~~~~~~~~~~~~~~~~~~~~~~~~~~~~~~~~~~~~~~~~~~~~~~~~~~~~~~~~~~~~~~~~~~~~~~~~~~~~~~~~~~~<1379     1410<~~~~~~~~~~~~~~~~~~~~~~~~~~~~~~~~~~~~~~~~~~~~~~~~~~~~~~~~~~~~~~~~~~~~~~~~~~~~~~~~~~~~~~~~~~~~~~~~~~~~<1410                   *         *         *         *         *         *         *         *         *         *   501>gaaggcaaagatgacagagcagaaagccctagtaaagcgtattacaaatgaaaccaagattcagattgcgatctctttaaagggtggtcccctagcgata>600      1438<~~~~~~~~~~~~~~~~~~~~~~~~~~~~~~~~~~~~~~~~~~~~~~~~~~~~~~~~~~~~~~~~~~~~~~~~~~~~~~~~~~~~~~~~~~~~~~~~~~~~<1438     1468<~~~~~~~~~~~~~~~~~~~~~~~~~~~~~~~~~~~~~~~~~~~~~~~~~~~~~~~~~~~~~~~~~~~~~~~~~~~~~~~~~~~~~~~~~~~~~~~~~~~~<1468     1464<~~~~~~~~~~~~~~~~~~~~~~~~~~~~~~~~~~~~~~~~~~~~~~~~~~~~~~~~~~~~~~~~~~~~~~~~~~~~~~~~~~~~~~~~~~~~~~~~~~~~<1464     1407<~~~~~~~~~~~~~~~~~~~~~~~~~~~~~~~~~~~~~~~~~~~~~~~~~~~~~~~~~~~~~~~~~~~~~~~~~~~~~~~~~~~~~~~~~~~~~~~~~~~~<1407     1379<~~~~~~~~~~~~~~~~~~~~~~~~~~~~~~~~~~~~~~~~~~~~~~~~~~~~~~~~~~~~~~~~~~~~~~~~~~~~~~~~~~~~~~~~~~~~~~~~~~~~<1379     1410<~~~~~~~~~~~~~~~~~~~~~~~~~~~~~~~~~~~~~~~~~~~~~~~~~~~~~~~~~~~~~~~~~~~~~~~~~~~~~~~~~~~~~~~~~~~~~~~~~~~~<1410                   *         *         *         *         *         *         *         *         *         *   601>gagcactcgatcttcccagaaaaagaggcagaagcagtagcagaacaggccacacaatcgcaagtgattaacgtccacacaggtatagggtttctggacc>700      1438<~~~~~~~~~~~~~~~~~~~~~~~~~~~~~~~~~~~~~~~~~~~~~~~~~~~~~~~~~~~~~~~~~~~~~~~~~~~~~~~~~~~~~~~~~~~~~~~~~~~~<1438     1468<~~~~~~~~~~~~~~~~~~~~~~~~~~~~~~~~~~~~~~~~~~~~~~~~~~~~~~~~~~~~~~~~~~~~~~~~~~~~~~~~~~~~~~~~~~~~~~~~~~~~<1468     1464<~~~~~~~~~~~~~~~~~~~~~~~~~~~~~~~~~~~~~~~~~~~~~~~~~~~~~~~~~~~~~~~~~~~~~~~~~~~~~~~~~~~~~~~~~~~~~~~~~~~~<1464     1407<~~~~~~~~~~~~~~~~~~~~~~~~~~~~~~~~~~~~~~~~~~~~~~~~~~~~~~~~~~~~~~~~~~~~~~~~~~~~~~~~~~~~~~~~~~~~~~~~~~~~<1407     1379<~~~~~~~~~~~~~~~~~~~~~~~~~~~~~~~~~~~~~~~~~~~~~~~~~~~~~~~~~~~~~~~~~~~~~~~~~~~~~~~~~~~~~~~~~~~~~~~~~~~~<1379     1410<~~~~~~~~~~~~~~~~~~~~~~~~~~~~~~~~~~~~~~~~~~~~~~~~~~~~~~~~~~~~~~~~~~~~~~~~~~~~~~~~~~~~~~~~~~~~~~~~~~~~<1410                   *         *         *         *         *         *         *         *         *         *   701>atatgatacatgctctggccaagcattccggctggtcgctaatcgttgagtgcattggtgacttacacatagacgaccatcacaccactgaagactgcgg>800      1438<~~~~~~~~~~~~~~~~~~~~~~~~~~~~~~~~~~~~~~~~~~~~~~~~~~~~~~~~~~~~~~~~~~~~~~~~~~~~~~~~~~~~~~~~~~~~~~~~~~~~<1438     1468<~~~~~~~~~~~~~~~~~~~~~~~~~~~~~~~~~~~~~~~~~~~~~~~~~~~~~~~~~~~~~~~~~~~~~~~~~~~~~~~~~~~~~~~~~~~~~~~~~~~~<1468     1464<~~~~~~~~~~~~~~~~~~~~~~~~~~~~~~~~~~~~~~~~~~~~~~~~~~~~~~~~~~~~~~~~~~~~~~~~~~~~~~~~~~~~~~~~~~~~~~~~~~~~<1464     1407<~~~~~~~~~~~~~~~~~~~~~~~~~~~~~~~~~~~~~~~~~~~~~~~~~~~~~~~~~~~~~~~~~~~~~~~~~~~~~~~~~~~~~~~~~~~~~~~~~~~~<1407     1379<~~~~~~~~~~~~~~~~~~~~~~~~~~~~~~~~~~~~~~~~~~~~~~~~~~~~~~~~~~~~~~~~~~~~~~~~~~~~~~~~~~~~~~~~~~~~~~~~~~~~<1379     1410<~~~~~~~~~~~~~~~~~~~~~~~~~~~~~~~~~~~~~~~~~~~~~~~~~~~~~~~~~~~~~~~~~~~~~~~~~~~~~~~~~~~~~~~~~~~~~~~~~~~~<1410                   *         *         *         *         *         *         *         *         *         *   801>gattgctctcggtcaagcttttaaagaggccctaggggccgtgcgtggagtaaaaaggtttggatcaggatttgcgcctttggatgaggcactttccaga>900      1438<~~~~~~~~~~~~~~~~~~~~~~~~~~~~~~~~~~~~~~~~~~~~~~~~~~~~~~~~~~~~~~~~~~~~~~~~~~~~~~~~~~~~~~~~~~~~~~~~~~~~<1438     1468<~~~~~~~~~~~~~~~~~~~~~~~~~~~~~~~~~~~~~~~~~~~~~~~~~~~~~~~~~~~~~~~~~~~~~~~~~~~~~~~~~~~~~~~~~~~~~~~~~~~~<1468     1464<~~~~~~~~~~~~~~~~~~~~~~~~~~~~~~~~~~~~~~~~~~~~~~~~~~~~~~~~~~~~~~~~~~~~~~~~~~~~~~~~~~~~~~~~~~~~~~~~~~~~<1464     1407<~~~~~~~~~~~~~~~~~~~~~~~~~~~~~~~~~~~~~~~~~~~~~~~~~~~~~~~~~~~~~~~~~~~~~~~~~~~~~~~~~~~~~~~~~~~~~~~~~~~~<1407     1379<~~~~~~~~~~~~~~~~~~~~~~~~~~~~~~~~~~~~~~~~~~~~~~~~~~~~~~~~~~~~~~~~~~~~~~~~~~~~~~~~~~~~~~~~~~~~~~~~~~~~<1379     1410<~~~~~~~~~~~~~~~~~~~~~~~~~~~~~~~~~~~~~~~~~~~~~~~~~~~~~~~~~~~~~~~~~~~~~~~~~~~~~~~~~~~~~~~~~~~~~~~~~~~~<1410                   *         *         *         *         *         *         *         *         *         *   901>gcggtggtagatctttcgaacaggccgtacgcagttgtcgaacttggtttgcaaagggagaaagtaggagatctctcttgcgagatgatcccgcattttc>1000     1438<~~~~~~~~~~~~~~~~~~~~~~~~~~~~~~~~~~~~~~~~~~~~~~~~~~~~~~~~~~~~~~~~~~~~~~~~~~~~~~~~~~~~~~~~~~~~~~~~~~~~<1438     1468<~~~~~~~~~~~~~~~~~~~~~~~~~~~~~~~~~~~~~~~~~~~~~~~~~~~~~~~~~~~~~~~~~~~~~~~~~~~~~~~~~~~~~~~~~~~~~~~~~~~~<1468     1464<~~~~~~~~~~~~~~~~~~~~~~~~~~~~~~~~~~~~~~~~~~~~~~~~~~~~~~~~~~~~~~~~~~~~~~~~~~~~~~~~~~~~~~~~~~~~~~~~~~~~<1464     1407<~~~~~~~~~~~~~~~~~~~~~~~~~~~~~~~~~~~~~~~~~~~~~~~~~~~~~~~~~~~~~~~~~~~~~~~~~~~~~~~~~~~~~~~~~~~~~~~~~~~~<1407     1379<~~~~~~~~~~~~~~~~~~~~~~~~~~~~~~~~~~~~~~~~~~~~~~~~~~~~~~~~~~~~~~~~~~~~~~~~~~~~~~~~~~~~~~~~~~~~~~~~~~~~<1379     1410<~~~~~~~~~~~~~~~~~~~~~~~~~~~~~~~~~~~~~~~~~~~~~~~~~~~~~~~~~~~~~~~~~~~~~~~~~~~~~~~~~~~~~~~~~~~~~~~~~~~~<1410                   *         *         *         *         *         *         *         *         *         *  1001>ttgaaagctttgcagaggctagcagaattaccctccacgttgattgtctgcgaggcaagaatgatcatcaccgtagtgagagtgcgttcaaggctcttgc>1100     1438<~~~~~~~~~~~~~~~~~~~~~~~~~~~~~~~~~~~~~~~~~~~~~~~~~~~~~~~~~~~~~~~~~~~~~~~~~~~~~~~~~~~~~~~~~~~~~~~~~~~~<1438     1468<~~~~~~~~~~~~~~~~~~~~~~~~~~~~~~~~~~~~~~~~~~~~~~~~~~~~~~~~~~~~~~~~~~~~~~~~~~~~~~~~~~~~~~~~~~~~~~~~~~~~<1468     1464<~~~~~~~~~~~~~~~~~~~~~~~~~~~~~~~~~~~~~~~~~~~~~~~~~~~~~~~~~~~~~~~~~~~~~~~~~~~~~~~~~~~~~~~~~~~~~~~~~~~~<1464     1407<~~~~~~~~~~~~~~~~~~~~~~~~~~~~~~~~~~~~~~~~~~~~~~~~~~~~~~~~~~~~~~~~~~~~~~~~~~~~~~~~~~~~~~~~~~~~~~~~~~~~<1407     1379<~~~~~~~~~~~~~~~~~~~~~~~~~~~~~~~~~~~~~~~~~~~~~~~~~~~~~~~~~~~~~~~~~~~~~~~~~~~~~~~~~~~~~~~~~~~~~~~~~~~~<1379     1410<~~~~~~~~~~~~~~~~~~~~~~~~~~~~~~~~~~~~~~~~~~~~~~~~~~~~~~~~~~~~~~~~~~~~~~~~~~~~~~~~~~~~~~~~~~~~~~~~~~~~<1410                   *         *         *         *         *         *         *         *         *         *  1101>ggttgccataagagaagccacctcgcccaatggtaccaacgatgttccctccaccaaaggtgttcttatgtaggcgaatttcttatgatttatgattttt>1200     1438<~~~~~~~~~~~~~~~~~~~~~~~~~~~~~~~~~~~~~~~~~~~~~~~~~~~~~~~~~~~~~~~~~~~~~~~~~~~~~~~~~~~~~~~~~~~~~~~~~~~~<1438     1468<~~~~~~~~~~~~~~~~~~~~~~~~~~~~~~~~~~~~~~~~~~~~~~~~~~~~~~~~~~~~~~~~~~~~~~~~~~~~~~~~~~~~~~~~~~~~~~~~~~~~<1468     1464<~~~~~~~~~~~~~~~~~~~~~~~~~~~~~~~~~~~~~~~~~~~~~~~~~~~~~~~~~~~~~~~~~~~~~~~~~~~~~~~~~~~~~~~~~~~~~~~~~~~~<1464     1407<~~~~~~~~~~~~~~~~~~~~~~~~~~~~~~~~~~~~~~~~~~~~~~~~~~~~~~~~~~~~~~~~~~~~~~~~~~~~~~~~~~~~~~~~~~~~~~~~~~~~<1407     1379<~~~~~~~~~~~~~~~~~~~~~~~~~~~~~~~~~~~~~~~~~~~~~~~~~~~~~~~~~~~~~~~~~~~~~~~~~~~~~~~~~~~~~~~~~~~~~~~~~~~~<1379     1410<~~~~~~~~~~~~~~~~~~~~~~~~~~~~~~~~~~~~~~~~~~~~~~~~~~~~~~~~~~~~~~~~~~~~~~~~~~~~~~~~~~~~~~~~~~~~~~~~~~~~<1410                   *         *         *         *         *         *         *         *         *         *  1201>attattaaataagttataaaaaaaataagtgtatacaaattttaaagtgactcttaggttttaaaacgaaaattcttattcttgagtaactctttcctgt>1300     1438<~~~~~~~~~~~~~~~~~~~~~~~~~~~~~~~~~~~~~~~~~~~~~~~~~~~~~~~~~~~~~~~~~~~~~~~~~~~~~~~~~~~~~~~~~~~~~~~~~~~~<1438     1468<~~~~~~~~~~~~~~~~~~~~~~~~~~~~~~~~~~~~~~~~~~~~~~~~~~~~~~~~~~~~~~~~~~~~~~~~~~~~~~~~~~~~~~~~~~~~~~~~~~~~<1468     1464<~~~~~~~~~~~~~~~~~~~~~~~~~~~~~~~~~~~~~~~~~~~~~~~~~~~~~~~~~~~~~~~~~~~~~~~~~~~~~~~~~~~~~~~~~~~~~~~~~~~~<1464     1407<~~~~~~~~~~~~~~~~~~~~~~~~~~~~~~~~~~~~~~~~~~~~~~~~~~~~~~~~~~~~~~~~~~~~~~~~~~~~~~~~~~~~~~~~~~~~~~~~~~~~<1407     1379<~~~~~~~~~~~~~~~~~~~~~~~~~~~~~~~~~~~~~~~~~~~~~~~~~~~~~~~~~~~~~~~~~~~~~~~~~~~~~~~~~~~~~~~~~~~~~~~~~~~~<1379     1410<~~~~~~~~~~~~~~~~~~~~~~~~~~~~~~~~~~~~~~~~~~~~~~~~~~~~~~~~~~~~~~~~~~~~~~~~~~~~~~~~~~~~~~~~~~~~~~~~~~~~<1410                   *         *         *         *         *         *         *         *         *         *  1301>aggtcaggttgctttctcaggtatagcatgaggtcgctcttattgaccacacctcaagaaatgatggtaaatgaaataggaaatcaaggagcatgaaggc>1400     1438<~~~~~~~~~~~~~~~~~~~~~~~~~~~~~~~~~~~~~~~~~~~~~~~~~~~~~~~~~~~~~~~~~~~~~~~~~~~~~~~~~~~~~~~~~~~~~~~~~~~~<1438     1468<~~~~~~~~~~~~~~~~~~~~~~~~~~~~~~~~~~~~~~~~~~~~~~~~~~~~~~~~~~~~~~~~~~~~~~~~~~~~~~~~~~~~~~~~~~~~~~~~~~~~<1468     1464<~~~~~~~~~~~~~~~~~~~~~~~~~~~~~~~~~~~~~~~~~~~~~~~~~~~~~~~~~~~~~~~~~~~~~~~~~~~~~~~~~~~~~~~~~~~~~~~~~~~~<1464     1407<~~~~~~~~~~~~~~~~~~~~~~~~~~~~~~~~~~~~~~~~~~~~~~~~~~~~~~~~~~~~~~~~~~~~~~~~~~~~~~~~~~~~~~~~~~~~~~~~~~~~<1407     1379<~~~~~~~~~~~~~~~~~~~~~~~~~~~~~~~~~~~~~~~~~~~~~~~~~~~~~~~~~~~~~~~~~~~~~~~~~~~~~~~~~~~~~~~~~~~~~~~~~~~~<1379     1410<~~~~~~~~~~~~~~~~~~~~~~~~~~~~~~~~~~~~~~~~~~~~~~~~~~~~~~~~~~~~~~~~~~~~~~~~~~~~~~~~~~~~~~~~~~~~~~~~~~~~<1410                   *         *         *         *         *         *         *         *         *         *  1401>aaaagacaaatataagggtcgaacgaaaaataaagtgaaaagtgttgatatgatgtatttggctttgcggcgccgaaaaaacgagtttacgcaattgcac>1500     1438<~~~~~~~~~~~~~~~~~~~~~~~~~~~~~~~~~~~~~~~~~~~~~~~~~~~~~~~~~~~~~~~~~~~~~~~~~~~~~~~~~~~~~~~~~~~~~~~~~~~~<1438     1468<~~~~~~~~~~~~~~~~~~~~~~~~~~~~~~~~~~~~~~~~~~~~~~~~~~~~~~~~~~~~~~~~~~~~~~~~~~~~~~~~~~~~~~~~~~~~~~~~~~~~<1468     1464<~~~~~~~~~~~~~~~~~~~~~~~~~~~~~~~~~~~~~~~~~~~~~~~~~~~~~~~~~~~~~~~~~~~~~~~~~~~~~~~~~~~~~~~~~~~~~~~~~~~~<1464     1407<~~~~~~~~~~~~~~~~~~~~~~~~~~~~~~~~~~~~~~~~~~~~~~~~~~~~~~~~~~~~~~~~~~~~~~~~~~~~~~~~~~~~~~~~~~~~~~~~~~~~<1407     1379<~~~~~~~~~~~~~~~~~~~~~~~~~~~~~~~~~~~~~~~~~~~~~~~~~~~~~~~~~~~~~~~~~~~~~~~~~~~~~~~~~~~~~~~~~~~~~~~~~~~~<1379     1410<~~~~~~~~~~~~~~~~~~~~~~~~~~~~~~~~~~~~~~~~~~~~~~~~~~~~~~~~~~~~~~~~~~~~~~~~~~~~~~~~~~~~~~~~~~~~~~~~~~~~<1410                   *         *         *         *         *         *         *         *         *         *  1501>aatcatgctgactctgtggcggacccgcgctcttgccggcccggcgataacgctgggcgtgaggctgtgcccggcggagttttttgcgcctgcattttcc>1600     1438<~~~~~~~~~~~~~~~~~~~~~~~~~~~~~~~~~~~~~~~~~~~~~~~~~~~~~~~~~~~~~~~~~~~~~~~~~~~~~~~~~~~~~~~~~~~~~~~~~~~~<1438     1468<~~~~~~~~~~~~~~~~~~~~~~~~~~~~~~~~~~~~~~~~~~~~~~~~~~~~~~~~~~~~~~~~~~~~~~~~~~~~~~~~~~~~~~~~~~~~~~~~~~~~<1468     1464<~~~~~~~~~~~~~~~~~~~~~~~~~~~~~~~~~~~~~~~~~~~~~~~~~~~~~~~~~~~~~~~~~~~~~~~~~~~~~~~~~~~~~~~~~~~~~~~~~~~~<1464     1407<~~~~~~~~~~~~~~~~~~~~~~~~~~~~~~~~~~~~~~~~~~~~~~~~~~~~~~~~~~~~~~~~~~~~~~~~~~~~~~~~~~~~~~~~~~~~~~~~~~~~<1407     1379<~~~~~~~~~~~~~~~~~~~~~~~~~~~~~~~~~~~~~~~~~~~~~~~~~~~~~~~~~~~~~~~~~~~~~~~~~~~~~~~~~~~~~~~~~~~~~~~~~~~~<1379     1410<~~~~~~~~~~~~~~~~~~~~~~~~~~~~~~~~~~~~~~~~~~~~~~~~~~~~~~~~~~~~~~~~~~~~~~~~~~~~~~~~~~~~~~~~~~~~~~~~~~~~<1410                   *         *         *         *         *         *         *         *         *         *  1601>aaggtttaccctgcgctaaggggcgagattggagaagcaataagaatgccggttggggttgcgatgatgacgaccacgacaactggtgtcattatttaag>1700     1438<~~~~~~~~~~~~~~~~~~~~~~~~~~~~~~~~~~~~~~~~~~~~~~~~~~~~~~~~~~~~~~~~~~~~~~~~~~~~~~~~~~~~~~~~~~~~~~~~~~~~<1438     1468<~~~~~~~~~~~~~~~~~~~~~~~~~~~~~~~~~~~~~~~~~~~~~~~~~~~~~~~~~~~~~~~~~~~~~~~~~~~~~~~~~~~~~~~~~~~~~~~~~~~~<1468     1464<~~~~~~~~~~~~~~~~~~~~~~~~~~~~~~~~~~~~~~~~~~~~~~~~~~~~~~~~~~~~~~~~~~~~~~~~~~~~~~~~~~~~~~~~~~~~~~~~~~~~<1464     1407<~~~~~~~~~~~~~~~~~~~~~~~~~~~~~~~~~~~~~~~~~~~~~~~~~~~~~~~~~~~~~~~~~~~~~~~~~~~~~~~~~~~~~~~~~~~~~~~~~~~~<1407     1379<~~~~~~~~~~~~~~~~~~~~~~~~~~~~~~~~~~~~~~~~~~~~~~~~~~~~~~~~~~~~~~~~~~~~~~~~~~~~~~~~~~~~~~~~~~~~~~~~~~~~<1379     1410<~~~~~~~~~~~~~~~~~~~~~~~~~~~~~~~~~~~~~~~~~~~~~~~~~~~~~~~~~~~~~~~~~~~~~~~~~~~~~~~~~~~~~~~~~~~~~~~~~~~~<1410                   *         *         *         *         *         *         *         *         *         *  1701>ttgccgaaagaacctgagtgcatttgcaacatgagtatactagaagaatgagccaagacttgcgagacgcgagtttgccggtggtgcgaacaatagagcg>1800     1438<~~~~~~~~~~~~~~~~~~~~~~~~~~~~~~~~~~~~~~~~~~~~~~~~~~~~~~~~~~~~~~~~~~~~~~~~~~~~~~~~~~~~~~~~~~~~~~~~~~~~<1438     1468<~~~~~~~~~~~~~~~~~~~~~~~~~~~~~~~~~~~~~~~~~~~~~~~~~~~~~~~~~~~~~~~~~~~~~~~~~~~~~~~~~~~~~~~~~~~~~~~~~~~~<1468     1464<~~~~~~~~~~~~~~~~~~~~~~~~~~~~~~~~~~~~~~~~~~~~~~~~~~~~~~~~~~~~~~~~~~~~~~~~~~~~~~~~~~~~~~~~~~~~~~~~~~~~<1464     1407<~~~~~~~~~~~~~~~~~~~~~~~~~~~~~~~~~~~~~~~~~~~~~~~~~~~~~~~~~~~~~~~~~~~~~~~~~~~~~~~~~~~~~~~~~~~~~~~~~~~~<1407     1379<~~~~~~~~~~~~~~~~~~~~~~~~~~~~~~~~~~~~~~~~~~~~~~~~~~~~~~~~~~~~~~~~~~~~~~~~~~~~~~~~~~~~~~~~~~~~~~~~~~~~<1379     1410<~~~~~~~~~~~~~~~~~~~~~~~~~~~~~~~~~~~~~~~~~~~~~~~~~~~~~~~~~~~~~~~~~~~~~~~~~~~~~~~~~~~~~~~~~~~~~~~~~~~~<1410                   *         *         *         *         *         *         *         *         *         *  1801>accatgaccttgaaggtgagacgcgcataaccgctagagtactttgaagaggaaacagcaatagggttgctaccagtataaatagacaggtacatacaac>1900     1438<~~~~~~~~~~~~~~~~~~~~~~~~~~~~~~~~~~~~~~~~~~~~~~~~~~~~~~~~~~~~~~~~~~~~~~~~~~~~~~~~~~~~~~~~~~~~~~~~~~~~<1438     1468<~~~~~~~~~~~~~~~~~~~~~~~~~~~~~~~~~~~~~~~~~~~~~~~~~~~~~~~~~~~~~~~~~~~~~~~~~~~~~~~~~~~~~~~~~~~~~~~~~~~~<1468     1464<~~~~~~~~~~~~~~~~~~~~~~~~~~~~~~~~~~~~~~~~~~~~~~~~~~~~~~~~~~~~~~~~~~~~~~~~~~~~~~~~~~~~~~~~~~~~~~~~~~~~<1464     1407<~~~~~~~~~~~~~~~~~~~~~~~~~~~~~~~~~~~~~~~~~~~~~~~~~~~~~~~~~~~~~~~~~~~~~~~~~~~~~~~~~~~~~~~~~~~~~~~~~~~~<1407     1379<~~~~~~~~~~~~~~~~~~~~~~~~~~~~~~~~~~~~~~~~~~~~~~~~~~~~~~~~~~~~~~~~~~~~~~~~~~~~~~~~~~~~~~~~~~~~~~~~~~~~<1379     1410<~~~~~~~~~~~~~~~~~~~~~~~~~~~~~~~~~~~~~~~~~~~~~~~~~~~~~~~~~~~~~~~~~~~~~~~~~~~~~~~~~~~~~~~~~~~~~~~~~~~~<1410                   *         *         *         *         *         *         *         *         *         *  1901>actggaaatggttgtctgtttgagtacgctttcaattcatttgggtgtgcactttattatgttacaatatggaagggaactttacacttctcctatgcac>2000     1438<~~~~~~~~~~~~~~~~~~~~~~~~~~~~~~~~~~~~~~~~~~~~~~~~~~~~~~~~~~~~~~~~~~~~~~~~~~~~~~~~~~~~~~~~~~~~~~~~~~~~<1438     1468<~~~~~~~~~~~~~~~~~~~~~~~~~~~~~~~~~~~~~~~~~~~~~~~~~~~~~~~~~~~~~~~~~~~~~~~~~~~~~~~~~~~~~~~~~~~~~~~~~~~~<1468     1464<~~~~~~~~~~~~~~~~~~~~~~~~~~~~~~~~~~~~~~~~~~~~~~~~~~~~~~~~~~~~~~~~~~~~~~~~~~~~~~~~~~~~~~~~~~~~~~~~~~~~<1464     1407<~~~~~~~~~~~~~~~~~~~~~~~~~~~~~~~~~~~~~~~~~~~~~~~~~~~~~~~~~~~~~~~~~~~~~~~~~~~~~~~~~~~~~~~~~~~~~~~~~~~~<1407     1379<~~~~~~~~~~~~~~~~~~~~~~~~~~~~~~~~~~~~~~~~~~~~~~~~~~~~~~~~~~~~~~~~~~~~~~~~~~~~~~~~~~~~~~~~~~~~~~~~~~~~<1379     1410<~~~~~~~~~~~~~~~~~~~~~~~~~~~~~~~~~~~~~~~~~~~~~~~~~~~~~~~~~~~~~~~~~~~~~~~~~~~~~~~~~~~~~~~~~~~~~~~~~~~~<1410                   *         *         *         *         *         *         *         *         *         *  2001>atatattaattaaagtccaatgctagtagagaaggggggtaacacccctccgcgctcttttccgatttttttctaaaccgtggaatatttcggatatcct>2100     1438<~~~~~~~~~~~~~~~~~~~~~~~~~~~~~~~~~~~~~~~~~~~~~~~~~~~~~~~~~~~~~~~~~~~~~~~~~~~~~~~~~~~~~~~~~~~~~~~~~~~~<1438     1468<~~~~~~~~~~~~~~~~~~~~~~~~~~~~~~~~~~~~~~~~~~~~~~~~~~~~~~~~~~~~~~~~~~~~~~~~~~~~~~~~~~~~~~~~~~~~~~~~~~~~<1468     1464<~~~~~~~~~~~~~~~~~~~~~~~~~~~~~~~~~~~~~~~~~~~~~~~~~~~~~~~~~~~~~~~~~~~~~~~~~~~~~~~~~~~~~~~~~~~~~~~~~~~~<1464     1407<~~~~~~~~~~~~~~~~~~~~~~~~~~~~~~~~~~~~~~~~~~~~~~~~~~~~~~~~~~~~~~~~~~~~~~~~~~~~~~~~~~~~~~~~~~~~~~~~~~~~<1407     1379<~~~~~~~~~~~~~~~~~~~~~~~~~~~~~~~~~~~~~~~~~~~~~~~~~~~~~~~~~~~~~~~~~~~~~~~~~~~~~~~~~~~~~~~~~~~~~~~~~~~~<1379     1410<~~~~~~~~~~~~~~~~~~~~~~~~~~~~~~~~~~~~~~~~~~~~~~~~~~~~~~~~~~~~~~~~~~~~~~~~~~~~~~~~~~~~~~~~~~~~~~~~~~~~<1410                   *         *         *         *         *         *         *         *         *         *  2101>tttgttgtttccgggtgtacaatatggacttcctcttttctggcaaccaaacccatacatcgggattcctataataccttcgttggtctccctaacatgt>2200     1438<~~~~~~~~~~~~~~~~~~~~~~~~~~~~~~~~~~~~~~~~~~~~~~~~~~~~~~~~~~~~~~~~~~~~~~~~~~~~~~~~~~~~~~~~~~~~~~~~~~~~<1438     1468<~~~~~~~~~~~~~~~~~~~~~~~~~~~~~~~~~~~~~~~~~~~~~~~~~~~~~~~~~~~~~~~~~~~~~~~~~~~~~~~~~~~~~~~~~~~~~~~~~~~~<1468     1464<~~~~~~~~~~~~~~~~~~~~~~~~~~~~~~~~~~~~~~~~~~~~~~~~~~~~~~~~~~~~~~~~~~~~~~~~~~~~~~~~~~~~~~~~~~~~~~~~~~~~<1464     1407<~~~~~~~~~~~~~~~~~~~~~~~~~~~~~~~~~~~~~~~~~~~~~~~~~~~~~~~~~~~~~~~~~~~~~~~~~~~~~~~~~~~~~~~~~~~~~~~~~~~~<1407     1379<~~~~~~~~~~~~~~~~~~~~~~~~~~~~~~~~~~~~~~~~~~~~~~~~~~~~~~~~~~~~~~~~~~~~~~~~~~~~~~~~~~~~~~~~~~~~~~~~~~~~<1379     1410<~~~~~~~~~~~~~~~~~~~~~~~~~~~~~~~~~~~~~~~~~~~~~~~~~~~~~~~~~~~~~~~~~~~~~~~~~~~~~~~~~~~~~~~~~~~~~~~~~~~~<1410                   *         *         *         *         *         *         *         *         *         *  2201>aggtggcggaggggagatatacaatagaacagataccagacaagacataatgggctaaacaagactacaccaattacactgcctcattgatggtggtaca>2300     1438<~~~~~~~~~~~~~~~~~~~~~~~~~~~~~~~~~~~~~~~~~~~~~~~~~~~~~~~~~~~~~~~~~~~~~~~~~~~~~~~~~~~~~~~~~~~~~~~~~~~~<1438     1468<~~~~~~~~~~~~~~~~~~~~~~~~~~~~~~~~~~~~~~~~~~~~~~~~~~~~~~~~~~~~~~~~~~~~~~~~~~~~~~~~~~~~~~~~~~~~~~~~~~~~<1468     1464<~~~~~~~~~~~~~~~~~~~~~~~~~~~~~~~~~~~~~~~~~~~~~~~~~~~~~~~~~~~~~~~~~~~~~~~~~~~~~~~~~~~~~~~~~~~~~~~~~~~~<1464     1407<~~~~~~~~~~~~~~~~~~~~~~~~~~~~~~~~~~~~~~~~~~~~~~~~~~~~~~~~~~~~~~~~~~~~~~~~~~~~~~~~~~~~~~~~~~~~~~~~~~~~<1407     1379<~~~~~~~~~~~~~~~~~~~~~~~~~~~~~~~~~~~~~~~~~~~~~~~~~~~~~~~~~~~~~~~~~~~~~~~~~~~~~~~~~~~~~~~~~~~~~~~~~~~~<1379     1410<~~~~~~~~~~~~~~~~~~~~~~~~~~~~~~~~~~~~~~~~~~~~~~~~~~~~~~~~~~~~~~~~~~~~~~~~~~~~~~~~~~~~~~~~~~~~~~~~~~~~<1410                   *         *         *         *         *         *         *         *         *         *  2301>taacgaactaatactgtagccctagacttgatagccatcatcatatcgaagtttcactaccctttttccatttgccatctattgaagtaataataggcgc>2400     1438<~~~~~~~~~~~~~~~~~~~~~~~~~~~~~~~~~~~~~~~~~~~~~~~~~~~~~~~~~~~~~~~~~~~~~~~~~~~~~~~~~~~~~~~~~~~~~~~~~~~~<1438     1468<~~~~~~~~~~~~~~~~~~~~~~~~~~~~~~~~~~~~~~~~~~~~~~~~~~~~~~~~~~~~~~~~~~~~~~~~~~~~~~~~~~~~~~~~~~~~~~~~~~~~<1468     1464<~~~~~~~~~~~~~~~~~~~~~~~~~~~~~~~~~~~~~~~~~~~~~~~~~~~~~~~~~~~~~~~~~~~~~~~~~~~~~~~~~~~~~~~~~~~~~~~~~~~~<1464     1407<~~~~~~~~~~~~~~~~~~~~~~~~~~~~~~~~~~~~~~~~~~~~~~~~~~~~~~~~~~~~~~~~~~~~~~~~~~~~~~~~~~~~~~~~~~~~~~~~~~~~<1407     1379<~~~~~~~~~~~~~~~~~~~~~~~~~~~~~~~~~~~~~~~~~~~~~~~~~~~~~~~~~~~~~~~~~~~~~~~~~~~~~~~~~~~~~~~~~~~~~~~~~~~~<1379     1410<~~~~~~~~~~~~~~~~~~~~~~~~~~~~~~~~~~~~~~~~~~~~~~~~~~~~~~~~~~~~~~~~~~~~~~~~~~~~~~~~~~~~~~~~~~~~~~~~~~~~<1410                   *         *         *         *         *         *         *         *         *         *  2401>atgcaacttcttttctttttttttcttttctctctcccccgttgttgtctcaccatatccgcaatgacaaaaaaatgatggaagacactaaaggaaaaaa>2500     1438<~~~~~~~~~~~~~~~~~~~~~~~~~~~~~~~~~~~~~~~~~~~~~~~~~~~~~~~~~~~~~~~~~~~~~~~~~~~~~~~~~~~~~~~~~~~~~~~~~~~~<1438     1468<~~~~~~~~~~~~~~~~~~~~~~~~~~~~~~~~~~~~~~~~~~~~~~~~~~~~~~~~~~~~~~~~~~~~~~~~~~~~~~~~~~~~~~~~~~~~~~~~~~~~<1468     1464<~~~~~~~~~~~~~~~~~~~~~~~~~~~~~~~~~~~~~~~~~~~~~~~~~~~~~~~~~~~~~~~~~~~~~~~~~~~~~~~~~~~~~~~~~~~~~~~~~~~~<1464     1407<~~~~~~~~~~~~~~~~~~~~~~~~~~~~~~~~~~~~~~~~~~~~~~~~~~~~~~~~~~~~~~~~~~~~~~~~~~~~~~~~~~~~~~~~~~~~~~~~~~~~<1407     1379<~~~~~~~~~~~~~~~~~~~~~~~~~~~~~~~~~~~~~~~~~~~~~~~~~~~~~~~~~~~~~~~~~~~~~~~~~~~~~~~~~~~~~~~~~~~~~~~~~~~~<1379     1410<~~~~~~~~~~~~~~~~~~~~~~~~~~~~~~~~~~~~~~~~~~~~~~~~~~~~~~~~~~~~~~~~~~~~~~~~~~~~~~~~~~~~~~~~~~~~~~~~~~~~<1410                   *         *         *         *         *         *         *         *         *         *  2501>ttaacgacaaagacagcaccaacagatgtcgttgttccagagctgatgaggggtatctcgaagcacacgaaactttttccttccttcattcacgcacact>2600     1438<~~~~~~~~~~~~~~~~~~~~~~~~~~~~~~~~~~~~~~~~~~~~~~~~~~~~~~~~~~~~~~~~~~~~~~~~~~~~~~~~~~~~~~~~~~~~~~~~~~~~<1438     1468<~~~~~~~~~~~~~~~~~~~~~~~~~~~~~~~~~~~~~~~~~~~~~~~~~~~~~~~~~~~~~~~~~~~~~~~~~~~~~~~~~~~~~~~~~~~~~~~~~~~~<1468     1464<~~~~~~~~~~~~~~~~~~~~~~~~~~~~~~~~~~~~~~~~~~~~~~~~~~~~~~~~~~~~~~~~~~~~~~~~~~~~~~~~~~~~~~~~~~~~~~~~~~~~<1464     1407<~~~~~~~~~~~~~~~~~~~~~~~~~~~~~~~~~~~~~~~~~~~~~~~~~~~~~~~~~~~~~~~~~~~~~~~~~~~~~~~~~~~~~~~~~~~~~~~~~~~~<1407     1379<~~~~~~~~~~~~~~~~~~~~~~~~~~~~~~~~~~~~~~~~~~~~~~~~~~~~~~~~~~~~~~~~~~~~~~~~~~~~~~~~~~~~~~~~~~~~~~~~~~~~<1379     1410<~~~~~~~~~~~~~~~~~~~~~~~~~~~~~~~~~~~~~~~~~~~~~~~~~~~~~~~~~~~~~~~~~~~~~~~~~~~~~~~~~~~~~~~~~~~~~~~~~~~~<1410                   *         *         *         *         *         *         *         *         *         *  2601>actctctaatgagcaacggtatacggccttccttccagttacttgaatttgaaataaaaaaaagtttgctgtcttgctatcaagtataaatagacctgca>2700     1438<~~~~~~~~~~~~~~~~~~~~~~~~~~~~~~~~~~~~~~~~~~~~~~~~~~~~~~~~~~~~~~~~~~~~~~~~~~~~~~~~~~~~~~~~~~~~~~~~~~~~<1438     1468<~~~~~~~~~~~~~~~~~~~~~~~~~~~~~~~~~~~~~~~~~~~~~~~~~~~~~~~~~~~~~~~~~~~~~~~~~~~~~~~~~~~~~~~~~~~~~~~~~~~~<1468     1464<~~~~~~~~~~~~~~~~~~~~~~~~~~~~~~~~~~~~~~~~~~~~~~~~~~~~~~~~~~~~~~~~~~~~~~~~~~~~~~~~~~~~~~~~~~~~~~~~~~~~<1464     1407<~~~~~~~~~~~~~~~~~~~~~~~~~~~~~~~~~~~~~~~~~~~~~~~~~~~~~~~~~~~~~~~~~~~~~~~~~~~~~~~~~~~~~~~~~~~~~~~~~~~~<1407     1379<~~~~~~~~~~~~~~~~~~~~~~~~~~~~~~~~~~~~~~~~~~~~~~~~~~~~~~~~~~~~~~~~~~~~~~~~~~~~~~~~~~~~~~~~~~~~~~~~~~~~<1379     1410<~~~~~~~~~~~~~~~~~~~~~~~~~~~~~~~~~~~~~~~~~~~~~~~~~~~~~~~~~~~~~~~~~~~~~~~~~~~~~~~~~~~~~~~~~~~~~~~~~~~~<1410                   *         *         *         *         *         *         *         *         *         *  2701>attattaatcttttgtttcctcgtcattgttctcgttccctttcttccttgtttctttttctgcacaatatttcaagctataccaagcatacaatcaact>2800     1438<~~~~~~~~~~~~~~~~~~~~~~~~~~~~~~~~~~~~~~~~~~~~~~~~~~~~~~~~~~~~~~~~~~~~~~~~~~~~~~~~~~~~~~~~~~~~~~~~~~~~<1438     1468<~~~~~~~~~~~~~~~~~~~~~~~~~~~~~~~~~~~~~~~~~~~~~~~~~~~~~~~~~~~~~~~~~~~~~~~~~~~~~~~~~~~~~~~~~~~~~~~~~~~~<1468     1464<~~~~~~~~~~~~~~~~~~~~~~~~~~~~~~~~~~~~~~~~~~~~~~~~~~~~~~~~~~~~~~~~~~~~~~~~~~~~~~~~~~~~~~~~~~~~~~~~~~~~<1464     1407<~~~~~~~~~~~~~~~~~~~~~~~~~~~~~~~~~~~~~~~~~~~~~~~~~~~~~~~~~~~~~~~~~~~~~~~~~~~~~~~~~~~~~~~~~~~~~~~~~~~~<1407     1379<~~~~~~~~~~~~~~~~~~~~~~~~~~~~~~~~~~~~~~~~~~~~~~~~~~~~~~~~~~~~~~~~~~~~~~~~~~~~~~~~~~~~~~~~~~~~~~~~~~~~<1379     1410<~~~~~~~~~~~~~~~~~~~~~~~~~~~~~~~~~~~~~~~~~~~~~~~~~~~~~~~~~~~~~~~~~~~~~~~~~~~~~~~~~~~~~~~~~~~~~~~~~~~~<1410                   *         *         *         *         *         *         *         *         *         *  2801>atctcatatacatctagaactagtggatcccccatcacaagtttgtacaaaaaagcaggcttcaaaatgatggcttcattgtcttgtgttgaagacaaga>2900     1438<~~~~~~~~~~~~~~~~~~~~~~~~~~~~~~~~~~~~~~~~~~~~~~~~~~~~~~~~~~~~~~~~~~~~~~~~~~~~~~~~~~~~~~~~~~~~~~~~~~~~<1438     1468<~~~~~~~~~~~~~~~~~~~~~~~~~~~~~~~~~~~~~~~~~~~~~~~~~~~~~~~~~~~~~~~~~~~~~~~~~~~~~~~~~~~~~~~~~~~~~~~~~~~~<1468     1464<~~~~~~~~~~~~~~~~~~~~~~~~~~~~~~~~~~~~~~~~~~~~~~~~~~~~~~~~~~~~~~~~~~~~~~~~~~~~~~~~~~~~~~~~~~~~~~~~~~~~<1464     1407<~~~~~~~~~~~~~~~~~~~~~~~~~~~~~~~~~~~~~~~~~~~~~~~~~~~~~~~~~~~~~~~~~~~~~~~~~~~~~~~~~~~~~~~~~~~~~~~~~~~~<1407     1379<~~~~~~~~~~~~~~~~~~~~~~~~~~~~~~~~~~~~~~~~~~~~~~~~~~~~~~~~~~~~~~~~~~~~~~~~~~~~~~~~~~~~~~~~~~~~~~~~~~~~<1379     1410<~~~~~~~~~~~~~~~~~~~~~~~~~~~~~~~~~~~~~~~~~~~~~~~~~~~~~~~~~~~~~~~~~~~~~~~~~~~~~~~~~~~~~~~~~~~~~~~~~~~~<1410                   *         *         *         *         *         *         *         *         *         *  2901>tgaaaacaagttgtttggttaatggtggaggaactataacaacaacaacatctcaatctaccttgcttgaagagatgaagctgttgaaagaccagtcagg>3000     1438<~~~~~~~~~~~~~~~~~~~~~~~~~~~~~~~~~~~~~~~~~~~~~~~~~~~~~~~~~~~~~~~~~~~~~~~~~~~~~~~~~~~~~~~~~~~~~~~~~~~~<1438     1468<~~~~~~~~~~~~~~~~~~~~~~~~~~~~~~~~~~~~~~~~~~~~~~~~~~~~~~~~~~~~~~~~~~~~~~~~~~~~~~~~~~~~~~~~~~~~~~~~~~~~<1468     1464<~~~~~~~~~~~~~~~~~~~~~~~~~~~~~~~~~~~~~~~~~~~~~~~~~~~~~~~~~~~~~~~~~~~~~~~~~~~~~~~~~~~~~~~~~~~~~~~~~~~~<1464     1407<~~~~~~~~~~~~~~~~~~~~~~~~~~~~~~~~~~~~~~~~~~~~~~~~~~~~~~~~~~~~~~~~~~~~~~~~~~~~~~~~~~~~~~~~~~~~~~~~~~~~<1407     1379<~~~~~~~~~~~~~~~~~~~~~~~~~~~~~~~~~~~~~~~~~~~~~~~~~~~~~~~~~~~~~~~~~~~~~~~~~~~~~~~~~~~~~~~~~~~~~~~~~~~~<1379     1410<~~~~~~~~~~~~~~~~~~~~~~~~~~~~~~~~~~~~~~~~~~~~~~~~~~~~~~~~~~~~~~~~~~~~~~~~~~~~~~~~~~~~~~~~~~~~~~~~~~~~<1410                   *         *         *         *         *         *         *         *         *         *  3001>tacaagaaagccggtaataaactcggagctatggcacgcttgtgcaggccctttggtgtgtctccctcaagttgggagcttagtgtattacttctcacaa>3100     1438<~~~~~~~~~~~~~~~~~~~~~~~~~~~~~~~~~~~~~~~~~~~~~~~~~~~~~~~~~~~~~~~~~~~~~~~~~~~~~~~~~~~~~~~~~~~~~~~~~~~~<1438     1468<~~~~~~~~~~~~~~~~~~~~~~~~~~~~~~~~~~~~~~~~~~~~~~~~~~~~~~~~~~~~~~~~~~~~~~~~~~~~~~~~~~~~~~~~~~~~~~~~~~~~<1468     1464<~~~~~~~~~~~~~~~~~~~~~~~~~~~~~~~~~~~~~~~~~~~~~~~~~~~~~~~~~~~~~~~~~~~~~~~~~~~~~~~~~~~~~~~~~~~~~~~~~~~~<1464     1407<~~~~~~~~~~~~~~~~~~~~~~~~~~~~~~~~~~~~~~~~~~~~~~~~~~~~~~~~~~~~~~~~~~~~~~~~~~~~~~~~~~~~~~~~~~~~~~~~~~~~<1407     1379<~~~~~~~~~~~~~~~~~~~~~~~~~~~~~~~~~~~~~~~~~~~~~~~~~~~~~~~~~~~~~~~~~~~~~~~~~~~~~~~~~~~~~~~~~~~~~~~~~~~~<1379     1410<~~~~~~~~~~~~~~~~~~~~~~~~~~~~~~~~~~~~~~~~~~~~~~~~~~~~~~~~~~~~~~~~~~~~~~~~~~~~~~~~~~~~~~~~~~~~~~~~~~~~<1410                   *         *         *         *         *         *         *         *         *         *  3101>ggtcatagcgagcaggttgctgtttcaaccagaagatcagcaacaacacaagttcctaattatccgaaccttccatctcagttgatgtgtcaagtccata>3200     1438<~~~~~~~~~~~~~~~~~~~~~~~~~~~~~~~~~~~~~~~~~~~~~~~~~~~~~~~~~~~~~~~~~~~~~~~~~~~~~~~~~~~~~~~~~~~~~~~~~~~~<1438     1468<~~~~~~~~~~~~~~~~~~~~~~~~~~~~~~~~~~~~~~~~~~~~~~~~~~~~~~~~~~~~~~~~~~~~~~~~~~~~~~~~~~~~~~~~~~~~~~~~~~~~<1468     1464<~~~~~~~~~~~~~~~~~~~~~~~~~~~~~~~~~~~~~~~~~~~~~~~~~~~~~~~~~~~~~~~~~~~~~~~~~~~~~~~~~~~~~~~~~~~~~~~~~~~~<1464     1407<~~~~~~~~~~~~~~~~~~~~~~~~~~~~~~~~~~~~~~~~~~~~~~~~~~~~~~~~~~~~~~~~~~~~~~~~~~~~~~~~~~~~~~~~~~~~~~~~~~~~<1407     1379<~~~~~~~~~~~~~~~~~~~~~~~~~~~~~~~~~~~~~~~~~~~~~~~~~~~~~~~~~~~~~~~~~~~~~~~~~~~~~~~~~~~~~~~~~~~~~~~~~~~~<1379     1410<~~~~~~~~~~~~~~~~~~~~~~~~~~~~~~~~~~~~~~~~~~~~~~~~~~~~~~~~~~~~~~~~~~~~~~~~~~~~~~~~~~~~~~~~~~~~~~~~~~~~<1410                   *         *         *         *         *         *         *         *         *         *  3201>atgttactcttcatgctgacaaagacagtgacgaaatctatgctcagatgagtcttcaacctgttcactctgagagagatgtgttccctgtaccagactt>3300     1438<~~~~~~~~~~~~~~~~~~~~~~~~~~~~~~~~~~~~~~~~~~~~~~~~~~~~~~~~~~~~~~~~~~~~~~~~~~~~~~~~~~~~~~~~~~~~~~~~~~~~<1438     1468<~~~~~~~~~~~~~~~~~~~~~~~~~~~~~~~~~~~~~~~~~~~~~~~~~~~~~~~~~~~~~~~~~~~~~~~~~~~~~~~~~~~~~~~~~~~~~~~~~~~~<1468     1464<~~~~~~~~~~~~~~~~~~~~~~~~~~~~~~~~~~~~~~~~~~~~~~~~~~~~~~~~~~~~~~~~~~~~~~~~~~~~~~~~~~~~~~~~~~~~~~~~~~~~<1464     1407<~~~~~~~~~~~~~~~~~~~~~~~~~~~~~~~~~~~~~~~~~~~~~~~~~~~~~~~~~~~~~~~~~~~~~~~~~~~~~~~~~~~~~~~~~~~~~~~~~~~~<1407     1379<~~~~~~~~~~~~~~~~~~~~~~~~~~~~~~~~~~~~~~~~~~~~~~~~~~~~~~~~~~~~~~~~~~~~~~~~~~~~~~~~~~~~~~~~~~~~~~~~~~~~<1379     1410<~~~~~~~~~~~~~~~~~~~~~~~~~~~~~~~~~~~~~~~~~~~~~~~~~~~~~~~~~~~~~~~~~~~~~~~~~~~~~~~~~~~~~~~~~~~~~~~~~~~~<1410                   *         *         *         *         *         *         *         *         *         *  3301>tggaatgctgagaggaagtaagcacccgactgagtttttctgcaaaacacttactgcaagtgacacaagcacacatggaggtttctcagtgccacgtaga>3400     1438<~~~~~~~~~~~~~~~~~~~~~~~~~~~~~~~~~~~~~~~~~~~~~~~~~~~~~~~~~~~~~~~~~~~~~~~~~~~~~~~~~~~~~~~~~~~~~~~~~~~~<1438     1468<~~~~~~~~~~~~~~~~~~~~~~~~~~~~~~~~~~~~~~~~~~~~~~~~~~~~~~~~~~~~~~~~~~~~~~~~~~~~~~~~~~~~~~~~~~~~~~~~~~~~<1468     1464<~~~~~~~~~~~~~~~~~~~~~~~~~~~~~~~~~~~~~~~~~~~~~~~~~~~~~~~~~~~~~~~~~~~~~~~~~~~~~~~~~~~~~~~~~~~~~~~~~~~~<1464     1407<~~~~~~~~~~~~~~~~~~~~~~~~~~~~~~~~~~~~~~~~~~~~~~~~~~~~~~~~~~~~~~~~~~~~~~~~~~~~~~~~~~~~~~~~~~~~~~~~~~~~<1407     1379<~~~~~~~~~~~~~~~~~~~~~~~~~~~~~~~~~~~~~~~~~~~~~~~~~~~~~~~~~~~~~~~~~~~~~~~~~~~~~~~~~~~~~~~~~~~~~~~~~~~~<1379     1410<~~~~~~~~~~~~~~~~~~~~~~~~~~~~~~~~~~~~~~~~~~~~~~~~~~~~~~~~~~~~~~~~~~~~~~~~~~~~~~~~~~~~~~~~~~~~~~~~~~~~<1410                   *         *         *         *         *         *         *         *         *         *  3401>gctgcagagaagctatttccaccattggactactcagcacagccgccaacgcaagagcttgtagttcgagatcttcatgagaatacttggacatttcgcc>3500     1438<~~~~~~~~~~~~~~~~~~~~~~~~~~~~~~~~~~~~~~~~~~~~~~~~~~~~~~~~~~~~~~~~~~~~~~~~~~~~~~~~~~~~~~~~~~~~~~~~~~~~<1438     1468<~~~~~~~~~~~~~~~~~~~~~~~~~~~~~~~~~~~~~~~~~~~~~~~~~~~~~~~~~~~~~~~~~~~~~~~~~~~~~~~~~~~~~~~~~~~~~~~~~~~~<1468     1464<~~~~~~~~~~~~~~~~~~~~~~~~~~~~~~~~~~~~~~~~~~~~~~~~~~~~~~~~~~~~~~~~~~~~~~~~~~~~~~~~~~~~~~~~~~~~~~~~~~~~<1464     1407<~~~~~~~~~~~~~~~~~~~~~~~~~~~~~~~~~~~~~~~~~~~~~~~~~~~~~~~~~~~~~~~~~~~~~~~~~~~~~~~~~~~~~~~~~~~~~~~~~~~~<1407     1379<~~~~~~~~~~~~~~~~~~~~~~~~~~~~~~~~~~~~~~~~~~~~~~~~~~~~~~~~~~~~~~~~~~~~~~~~~~~~~~~~~~~~~~~~~~~~~~~~~~~~<1379     1410<~~~~~~~~~~~~~~~~~~~~~~~~~~~~~~~~~~~~~~~~~~~~~~~~~~~~~~~~~~~~~~~~~~~~~~~~~~~~~~~~~~~~~~~~~~~~~~~~~~~~<1410                   *         *         *         *         *         *         *         *         *         *  3501>atatctaccgagggcaaccaaagagacatctcctaactacaggatggagtttgttcgttggatcgaagagattgagagctggggattctgttttgttcat>3600     1438<~~~~~~~~~~~~~~~~~~~~~~~~~~~~~~~~~~~~~~~~~~~~~~~~~~~~~~~~~~~~~~~~~~~~~~~~~~~~~~~~~~~~~~~~~~~~~~~~~~~~<1438     1468<~~~~~~~~~~~~~~~~~~~~~~~~~~~~~~~~~~~~~~~~~~~~~~~~~~~~~~~~~~~~~~~~~~~~~~~~~~~~~~~~~~~~~~~~~~~~~~~~~~~~<1468     1464<~~~~~~~~~~~~~~~~~~~~~~~~~~~~~~~~~~~~~~~~~~~~~~~~~~~~~~~~~~~~~~~~~~~~~~~~~~~~~~~~~~~~~~~~~~~~~~~~~~~~<1464     1407<~~~~~~~~~~~~~~~~~~~~~~~~~~~~~~~~~~~~~~~~~~~~~~~~~~~~~~~~~~~~~~~~~~~~~~~~~~~~~~~~~~~~~~~~~~~~~~~~~~~~<1407     1379<~~~~~~~~~~~~~~~~~~~~~~~~~~~~~~~~~~~~~~~~~~~~~~~~~~~~~~~~~~~~~~~~~~~~~~~~~~~~~~~~~~~~~~~~~~~~~~~~~~~~<1379     1410<~~~~~~~~~~~~~~~~~~~~~~~~~~~~~~~~~~~~~~~~~~~~~~~~~~~~~~~~~~~~~~~~~~~~~~~~~~~~~~~~~~~~~~~~~~~~~~~~~~~~<1410                   *         *         *         *         *         *         *         *         *         *  3601>cagggatgagaagtcacaacttatggtcggtgttaggcgtgccaatcgccaacaaacagcacttccttcatcagttctctcagcggatagtatgcacatc>3700     1438<~~~~~~~~~~~~~~~~~~~~~~~~~~~~~~~~~~~~~~~~~~~~~~~~~~~~~~~~~~~~~~~~~~~~~~~~~~~~~~~~~~~~~~~~~~~~~~~~~~~~<1438     1468<~~~~~~~~~~~~~~~~~~~~~~~~~~~~~~~~~~~~~~~~~~~~~~~~~~~~~~~~~~~~~~~~~~~~~~~~~~~~~~~~~~~~~~~~~~~~~~~~~~~~<1468     1464<~~~~~~~~~~~~~~~~~~~~~~~~~~~~~~~~~~~~~~~~~~~~~~~~~~~~~~~~~~~~~~~~~~~~~~~~~~~~~~~~~~~~~~~~~~~~~~~~~~~~<1464     1407<~~~~~~~~~~~~~~~~~~~~~~~~~~~~~~~~~~~~~~~~~~~~~~~~~~~~~~~~~~~~~~~~~~~~~~~~~~~~~~~~~~~~~~~~~~~~~~~~~~~~<1407     1379<~~~~~~~~~~~~~~~~~~~~~~~~~~~~~~~~~~~~~~~~~~~~~~~~~~~~~~~~~~~~~~~~~~~~~~~~~~~~~~~~~~~~~~~~~~~~~~~~~~~~<1379     1410<~~~~~~~~~~~~~~~~~~~~~~~~~~~~~~~~~~~~~~~~~~~~~~~~~~~~~~~~~~~~~~~~~~~~~~~~~~~~~~~~~~~~~~~~~~~~~~~~~~~~<1410                   *         *         *         *         *         *         *         *         *         *  3701>ggtgttcttgctgctgctgctcacgcaaccgccaaccgtactccttttttgatattctataatccaagagcttgtccagcagagttcgtgatccctctag>3800     1438<~~~~~~~~~~~~~~~~~~~~~~~~~~~~~~~~~~~~~~~~~~~~~~~~~~~~~~~~~~~~~~~~~~~~~~~~~~~~~~~~~~~~~~~~~~~~~~~~~~~~<1438     1468<~~~~~~~~~~~~~~~~~~~~~~~~~~~~~~~~~~~~~~~~~~~~~~~~~~~~~~~~~~~~~~~~~~~~~~~~~~~~~~~~~~~~~~~~~~~~~~~~~~~~<1468     1464<~~~~~~~~~~~~~~~~~~~~~~~~~~~~~~~~~~~~~~~~~~~~~~~~~~~~~~~~~~~~~~~~~~~~~~~~~~~~~~~~~~~~~~~~~~~~~~~~~~~~<1464     1407<~~~~~~~~~~~~~~~~~~~~~~~~~~~~~~~~~~~~~~~~~~~~~~~~~~~~~~~~~~~~~~~~~~~~~~~~~~~~~~~~~~~~~~~~~~~~~~~~~~~~<1407     1379<~~~~~~~~~~~~~~~~~~~~~~~~~~~~~~~~~~~~~~~~~~~~~~~~~~~~~~~~~~~~~~~~~~~~~~~~~~~~~~~~~~~~~~~~~~~~~~~~~~~~<1379     1410<~~~~~~~~~~~~~~~~~~~~~~~~~~~~~~~~~~~~~~~~~~~~~~~~~~~~~~~~~~~~~~~~~~~~~~~~~~~~~~~~~~~~~~~~~~~~~~~~~~~~<1410                   *         *         *         *         *         *         *         *         *         *  3801>ctaagtaccgtaaggcgatatgcgggtctcagctctcagttggtatgagatttggaatgatgtttgaaactgaagattccgggaaacgaaggtacatggg>3900     1438<~~~~~~~~~~~~~~~~~~~~~~~~~~~~~~~~~~~~~~~~~~~~~~~~~~~~~~~~~~~~~~~~~~~~~~~~~~~~~~~~~~~~~~~~~~~~~~~~~~~~<1438     1468<~~~~~~~~~~~~~~~~~~~~~~~~~~~~~~~~~~~~~~~~~~~~~~~~~~~~~~~~~~~~~~~~~~~~~~~~~~~~~~~~~~~~~~~~~~~~~~~~~~~~<1468     1464<~~~~~~~~~~~~~~~~~~~~~~~~~~~~~~~~~~~~~~~~~~~~~~~~~~~~~~~~~~~~~~~~~~~~~~~~~~~~~~~~~~~~~~~~~~~~~~~~~~~~<1464     1407<~~~~~~~~~~~~~~~~~~~~~~~~~~~~~~~~~~~~~~~~~~~~~~~~~~~~~~~~~~~~~~~~~~~~~~~~~~~~~~~~~~~~~~~~~~~~~~~~~~~~<1407     1379<~~~~~~~~~~~~~~~~~~~~~~~~~~~~~~~~~~~~~~~~~~~~~~~~~~~~~~~~~~~~~~~~~~~~~~~~~~~~~~~~~~~~~~~~~~~~~~~~~~~~<1379     1410<~~~~~~~~~~~~~~~~~~~~~~~~~~~~~~~~~~~~~~~~~~~~~~~~~~~~~~~~~~~~~~~~~~~~~~~~~~~~~~~~~~~~~~~~~~~~~~~~~~~~<1410                   *         *         *         *         *         *         *         *         *         *  3901>aactattgttggaatcagcgatttggatccgttgagatggcctggttctaagtggcgtaaccttcaggtagaatgggatgagcctggatgtaatgataaa>4000     1438<~~~~~~~~~~~~~~~~~~~~~~~~~~~~~~~~~~~~~~~~~~~~~~~~~~~~~~~~~~~~~~~~~~~~~~~~~~~~~~~~~~~~~~~~~~~~~~~~~~~~<1438     1468<~~~~~~~~~~~~~~~~~~~~~~~~~~~~~~~~~~~~~~~~~~~~~~~~~~~~~~~~~~~~~~~~~~~~~~~~~~~~~~~~~~~~~~~~~~~~~~~~~~~~<1468     1464<~~~~~~~~~~~~~~~~~~~~~~~~~~~~~~~~~~~~~~~~~~~~~~~~~~~~~~~~~~~~~~~~~~~~~~~~~~~~~~~~~~~~~~~~~~~~~~~~~~~~<1464     1407<~~~~~~~~~~~~~~~~~~~~~~~~~~~~~~~~~~~~~~~~~~~~~~~~~~~~~~~~~~~~~~~~~~~~~~~~~~~~~~~~~~~~~~~~~~~~~~~~~~~~<1407     1379<~~~~~~~~~~~~~~~~~~~~~~~~~~~~~~~~~~~~~~~~~~~~~~~~~~~~~~~~~~~~~~~~~~~~~~~~~~~~~~~~~~~~~~~~~~~~~~~~~~~~<1379     1410<~~~~~~~~~~~~~~~~~~~~~~~~~~~~~~~~~~~~~~~~~~~~~~~~~~~~~~~~~~~~~~~~~~~~~~~~~~~~~~~~~~~~~~~~~~~~~~~~~~~~<1410                   *         *         *         *         *         *         *         *         *         *  4001>cctactcgggtcagtccatgggatatcgaaacacctgaaagtctcttcatttttccttctctgacctcaggactcaaacgtcagctccatccatcttact>4100     1438<~~~~~~~~~~~~~~~~~~~~~~~~~~~~~~~~~~~~~~~~~~~~~~~~~~~~~~~~~~~~~~~~~~~~~~~~~~~~~~~~~~~~~~~~~~~~~~~~~~~~<1438     1468<~~~~~~~~~~~~~~~~~~~~~~~~~~~~~~~~~~~~~~~~~~~~~~~~~~~~~~~~~~~~~~~~~~~~~~~~~~~~~~~~~~~~~~~~~~~~~~~~~~~~<1468     1464<~~~~~~~~~~~~~~~~~~~~~~~~~~~~~~~~~~~~~~~~~~~~~~~~~~~~~~~~~~~~~~~~~~~~~~~~~~~~~~~~~~~~~~~~~~~~~~~~~~~~<1464     1407<~~~~~~~~~~~~~~~~~~~~~~~~~~~~~~~~~~~~~~~~~~~~~~~~~~~~~~~~~~~~~~~~~~~~~~~~~~~~~~~~~~~~~~~~~~~~~~~~~~~~<1407     1379<~~~~~~~~~~~~~~~~~~~~~~~~~~~~~~~~~~~~~~~~~~~~~~~~~~~~~~~~~~~~~~~~~~~~~~~~~~~~~~~~~~~~~~~~~~~~~~~~~~~~<1379     1410<~~~~~~~~~~~~~~~~~~~~~~~~~~~~~~~~~~~~~~~~~~~~~~~~~~~~~~~~~~~~~~~~~~~~~~~~~~~~~~~~~~~~~~~~~~~~~~~~~~~~<1410                   *         *         *         *         *         *         *         *         *         *  4101>ttgctggtgaaactgaatggggtagcttgataaaacggccacttatacgtgttcctgattccgcgaatgggattatgccatatgcatctttccctagtat>4200     1438<~~~~~~~~~~~~~~~~~~~~~~~~~~~~~~~~~~~~~~~~~~~~~~~~~~~~~~~~~~~~~~~~~~~~~~~~~~~~~~~~~~~~~~~~~~~~~~~~~~~~<1438     1468<~~~~~~~~~~~~~~~~~~~~~~~~~~~~~~~~~~~~~~~~~~~~~~~~~~~~~~~~~~~~~~~~~~~~~~~~~~~~~~~~~~~~~~~~~~~~~~~~~~~~<1468     1464<~~~~~~~~~~~~~~~~~~~~~~~~~~~~~~~~~~~~~~~~~~~~~~~~~~~~~~~~~~~~~~~~~~~~~~~~~~~~~~~~~~~~~~~~~~~~~~~~~~~~<1464     1407<~~~~~~~~~~~~~~~~~~~~~~~~~~~~~~~~~~~~~~~~~~~~~~~~~~~~~~~~~~~~~~~~~~~~~~~~~~~~~~~~~~~~~~~~~~~~~~~~~~~~<1407     1379<~~~~~~~~~~~~~~~~~~~~~~~~~~~~~~~~~~~~~~~~~~~~~~~~~~~~~~~~~~~~~~~~~~~~~~~~~~~~~~~~~~~~~~~~~~~~~~~~~~~~<1379     1410<~~~~~~~~~~~~~~~~~~~~~~~~~~~~~~~~~~~~~~~~~~~~~~~~~~~~~~~~~~~~~~~~~~~~~~~~~~~~~~~~~~~~~~~~~~~~~~~~~~~~<1410                   *         *         *         *         *         *         *         *         *         *  4201>ggcttcggagcagcttatgaaaatgatgatgaggcctcacaacaaccaaaatgtaccatctttcatgtctgagatgcagcagaatattgtaatggggaat>4300     1438<~~~~~~~~~~~~~~~~~~~~~~~~~~~~~~~~~~~~~~~~~~~~~~~~~~~~~~~~~~~~~~~~~~~~~~~~~~~~~~~~~~~~~~~~~~~~~~~~~~~~<1438     1468<~~~~~~~~~~~~~~~~~~~~~~~~~~~~~~~~~~~~~~~~~~~~~~~~~~~~~~~~~~~~~~~~~~~~~~~~~~~~~~~~~~~~~~~~~~~~~~~~~~~~<1468     1464<~~~~~~~~~~~~~~~~~~~~~~~~~~~~~~~~~~~~~~~~~~~~~~~~~~~~~~~~~~~~~~~~~~~~~~~~~~~~~~~~~~~~~~~~~~~~~~~~~~~~<1464     1407<~~~~~~~~~~~~~~~~~~~~~~~~~~~~~~~~~~~~~~~~~~~~~~~~~~~~~~~~~~~~~~~~~~~~~~~~~~~~~~~~~~~~~~~~~~~~~~~~~~~~<1407     1379<~~~~~~~~~~~~~~~~~~~~~~~~~~~~~~~~~~~~~~~~~~~~~~~~~~~~~~~~~~~~~~~~~~~~~~~~~~~~~~~~~~~~~~~~~~~~~~~~~~~~<1379     1410<~~~~~~~~~~~~~~~~~~~~~~~~~~~~~~~~~~~~~~~~~~~~~~~~~~~~~~~~~~~~~~~~~~~~~~~~~~~~~~~~~~~~~~~~~~~~~~~~~~~~<1410                   *         *         *         *         *         *         *         *         *         *  4301>ggaggtttgctaggagatatgaagatgcagcaacccctgatgatgaaccagaaatctgagatggtgcagccacaaaacaagctaacagtgaacccatctg>4400     1438<~~~~~~~~~~~~~~~~~~~~~~~~~~~~~~~~~~~~~~~~~~~~~~~~~~~~~~~~~~~~~~~~~~~~~~~~~~~~~~~~~~~~~~~~~~~~~~~~~~~~<1438     1468<~~~~~~~~~~~~~~~~~~~~~~~~~~~~~~~~~~~~~~~~~~~~~~~~~~~~~~~~~~~~~~~~~~~~~~~~~~~~~~~~~~~~~~~~~~~~~~~~~~~~<1468     1464<~~~~~~~~~~~~~~~~~~~~~~~~~~~~~~~~~~~~~~~~~~~~~~~~~~~~~~~~~~~~~~~~~~~~~~~~~~~~~~~~~~~~~~~~~~~~~~~~~~~~<1464     1407<~~~~~~~~~~~~~~~~~~~~~~~~~~~~~~~~~~~~~~~~~~~~~~~~~~~~~~~~~~~~~~~~~~~~~~~~~~~~~~~~~~~~~~~~~~~~~~~~~~~~<1407     1379<~~~~~~~~~~~~~~~~~~~~~~~~~~~~~~~~~~~~~~~~~~~~~~~~~~~~~~~~~~~~~~~~~~~~~~~~~~~~~~~~~~~~~~~~~~~~~~~~~~~~<1379     1410<~~~~~~~~~~~~~~~~~~~~~~~~~~~~~~~~~~~~~~~~~~~~~~~~~~~~~~~~~~~~~~~~~~~~~~~~~~~~~~~~~~~~~~~~~~~~~~~~~~~~<1410                   *         *         *         *         *         *         *         *         *         *  4401>cttctaatacgagtggccaagaacagaatctttcacagagtatgagtgctcctgctaaacctgagaactctacactctctggttgcagctctggtagagt>4500     1438<~~~~~~~~~~~~~~~~~~~~~~~~~~~~~~~~~~~~~~~~~~~~~~~~~~~~~~~~~~~~~~~~~~~~~~~~~~~~~~~~~~~~~~~~~~~~~~~~~~~~<1438     1467<~~~~~~~~~~~~~~~~~~~~~~~~~~~~~~~~~~~~~~~~~~~~~~~~~~~~~~~~~~~~~~~~~~~~~~~~~~~~~~~~~~~~~~~~~~~~~~~~~~CT<1466     1464<~~~~~~~~~~~~~~~~~~~~~~~~~~~~~~~~~~~~~~~~~~~~~~~~~~~~~~~~~~~~~~~~~~~~~~~~~~~~~~~~~~~~~~~~~~~~~~~~~~~~<1464     1407<~~~~~~~~~~~~~~~~~~~~~~~~~~~~~~~~~~~~~~~~~~~~~~~~~~~~~~~~~~~~~~~~~~~~~~~~~~~~~~~~~~~~~~~~~~~~~~~~~~~~<1407     1379<~~~~~~~~~~~~~~~~~~~~~~~~~~~~~~~~~~~~~~~~~~~~~~~~~~~~~~~~~~~~~~~~~~~~~~~~~~~~~~~~~~~~~~~~~~~~~~~~~~~~<1379     1410<~~~~~~~~~~~~~~~~~~~~~~~~~~~~~~~~~~~~~~~~~~~~~~~~~~~~~~~~~~~~~~~~~~~~~~~~~~~~~~~~~~~~~~~~~~~~~~~~~~~~<1410                   *         *         *         *             *         *           *         *          *     4501>ccaacatggacttgagcagtcaatggaacaggcaagccaggttact-ac-a--tccacagtgtgta-atgagga-aaaggttaatcagctactt-cagaa>4593     1437<~~~~~~~~~~~~~~~~~~~~~~~~~~~~~~GGCAAGCCAGGTTACTCACTATCTCCACAGTGTGTATATGA-GAGAAAGGTTATTCAGCTACTTCCAG-A<1370     1465<TGAGAGCACGTTCAATATGGAAACACGGCCAAGCCCAGTTATACTA-CA-T--CCCACAGTGTGTA-TATGAAG-AAAGGTTATTCAGCTATCT-CAGAA<1373     1463<~~~~~CAAATCATATGGACTTGAGTGACGTCTAATGGAAACAGCAG-CC-A--GTATACACTCCAC-GTGGTAA-TGAGGAATAGTTATTTCAG-TACTC<1376     1406<~~~~~~~~~~~~~~~~~~~~~~~~~~~~~~~~~~~~~~~~~~~~~~~~~~~~~~~~~~~~~~~~~C-AAGTGGT-GTAATGAGGAAAGTATCAC-GTACT<1375     1379<~~~~~~~~~~~~~~~~~~~~~~~~~~~~~~~~~~~~~~~~~~~~~~~~~~~~~~~~~~~~~~~~~~~~~~~~~~~~~~~~~~~~~~~~~~~~~~~~~~~~<1379     1410<~~~~~~~~~~~~~~~~~~~~~~~~~~~~~~~~~~~~~~~~~~~~~~~~~~~~~~~~~~~~~~~~~~~~~~~~~~~~~~~~~~~~~~~~~~~~~~~~~~~~<1410                *         *         *          *         *         *         *          *         *         *   4594>accgggtgcttcgtcgcctgtacaagctgat-caatgtcttgacattactcatcagatttaccaaccacag-tctgatccaataaatggattctctttcc>4691     1369<ACC-GGTGC-TCGTCGCCTGTACAAGCTGATTC-ATGTCTTGACA-TACTCATCAGATTTACC-ACCACAGTTCTG-TTCAATAAAT-GATTCTCTTT-C<1278     1372<CCCGGTTGCTTCTGTTCGCCTTACAAGCTGA-TTCAATTCTTGACATTATCATCAGATTTACCACCACACA-GTTCTGTTCCAATAATGATTCTCCTTCC<1275     1375<AGAACCCGGTGCTCTCGTTCGCCTGTACAAG-CTGATCATGTCTTGACAATACTTCATCAGATTTACACAC-AGTCTGTTCAATAATGGATTCTCTCTTT<1278     1374<CAGAAACGGTTGGCTTTCGTGCCTGTAACAA-GGTGGATTCAATGTCTTGACATACTCATCAGATTACACA-CAGTCTGATCAATAATGGATCTTCTTTG<1277     1378<~~~~~~~~~~~~~~~~~~~~~~~~~~~~~~~~~~~~~~~~~~~~~~~~~~~~~~CATGTGTCTGTGACATA-TTCATCAGCTACACACAGTCGATTCATA<1334     1409<~~~~~~~~~~~~~~~~~~~~~~~~~~~ACGA-TCTACAGTGACCTGTCAGCTGTACAGTCCTGAACATATC-CTCAGGTACATCCAGGTTCGATCCAATA<1339                  *         *         *          *          *         *           *         *         *         4692>tggaaactgatgagctgacatcacaagtct-cttccttccagtc-tcttgccggatcatacaa-gcaac-cattcattctatcctcccaggattcttcag>4787     1277<TGGAAACTGATGAGCTGACATCACAAGTCT-CTTC--TGCAGTC-TCTTGCC-GATCATACAA-GCA--GCATTCATTCT-TTCTCCCAGGATTCTTCAG<1187     1274<TGGAAACTGATGAGCTGACATCACAAGTCT-CT-CCT--CAGTC-TC-TGCCGGATCATACAA-GCAAC-CATTCATTCTATC-TCCCAGGATTC-TCAG<1185     1277<CTGAAACTGATGAGCTGACATCACAAGTCTTCTTCC-TCCAGTC-TC-TGC--GATCATACAA-GCAAC-CATTCATTCTATCCTCC-AGGA-TC-TCAG<1188     1276<CTGAACTGATGAGCTGAACATCACAAGTCT-CTTC--TGCAGTCTTCTTGCGGGATCATACAAGGCA--GCA-TCATTCTAT-CTCCCAGGA-TC-TCAG<1186     1333<TGATTTCTCTTACCTTGACTGATGAGCTGA-CATCCCAAGTTCT-CTTCTGCAGTCTCTTGCG-ATCAT-ACCAGCATCAATCATCTATTCCTCCAGATT<1238     1338<ATTGATTCTCTTGCTGACTGATGAGGCTGA-CCATCACAAGTCT-CTCCTGCAGTCTCTTGCG-ATCAT-ACAAGCACCATCATTCTTATCCTCCCAGAT<1243            *         *          *         *         *         *         *         *         *         *        4788>ctgttgtgttaccggattc-cacaaactcaccgctgtttcatgatgtgtgggacactcagttgaacggtctcaagtttgaccagttcagtcccttgatgc>4886     1186<CTGTTGTGTTACCGGATTC-CACAAACTCACCGCTGTTTCATGATGTGTGGGACACTCAGTTGAACGGTCTCAAGTTTGACCAGTTCAGTCCCTTGATGC<1088     1184<CTG-TGTG-TACCGGA-TC-CACAAACTCACCGCTGTTTCATGATGTGTGGGACACTCAGTTGAACGGTCTCAAGTTTGACCAGTTCAGTCCCTTGATGC<1089     1187<CTGTTGTGTTACCGGATTC-CACAAACTCACCGCTGTTTCATGATGTGTGGGACACTCAGTTGAACGGTCTCAAGTTTGACCAGTTCAGTCCCTTGATGC<1089     1185<CTGTTGTGTTA-CGGAT--GCACAAACTCACCGCTGTTTCATGATGTGTGGGACACTCAGTTGAACGGTCTCAAGTTTGACCAGTTCAGTCCCTTGATGC<1089     1237<CTTCAGCTGTGGTACTGAT-CCACAACTCACCGCTGTTTCATGATGTGTGGGACA-TCAGTTGAAC-GTCTCAAGTTTGA-CAG-TCAGTCCCTTGATGC<1143     1242<CTCAGCTGTGTGTACGATC-CACCAACTCCACCGCTGTTCATGATGTGTGGGACACTCAGT-GAACGGTCTCAAGTTTGATCAGTTCAGTCCCTTGATGC<1145             *         *         *         *         *         *         *         *         *         *        4887>agcaggacctttatgctagtcagaatatctgtatgagtaatagcacaaccagtaacattctagatcctccactctcaaacacagtccttgatgacttctg>4986     1087<AGCAGGACCTTTATGCTAGTCAGAATATCTGTATGAGTAATAGCACAACCAGTAACATTCTAGATCCTCCACTCTCAAACACAGTCCTTGATGACTTCTG<988      1088<AGCAGGACCTTTATGCTAGTCAGAATATCTGTATGAGTAATAGCACAACCAGTAACATTCTAGATCCTCCACTCTCAAACACAGTCCTTGATGACTTCTG<989      1088<AGCAGGACCTTTATGCTAGTCAGAATATCTGTATGAGTAATAGCACAACCAGTAACATTCTAGATCCTCCACTCTCAAACACAGTCCTTGATGACTTCTG<989      1088<AGCAGGACCTT-ATGCTAGTCAGAATATCTGTATGAGTAATAGCACAACCAGTAACATTCTAGATCCTCCACTCTCAAACACAGTCCTTGATGACTTCTG<990      1142<AGCAGGACCTTTATGCTAGTCAGA-TATCTGTATGAGT-ATAGCACAACCAGTAACATTCTAGATCCTCCACTCTCAAACACAGTCCTTGATGACTTCTG<1045     1144<AGCAGGACCTTTATGCTAGTCAGA-TATCTGTATGAGTAATAGCAC-ACCAGTAACATTCTAGATCCTCCACTCTCAAACACAGTCCTTGATGACTTCTG<1047             *         *         *         *          *         *         *         *         *         *       4987>tgccatcaaagacactgatttccagaaccaccctt-ctggttgtttggttggaaacaacaacactagctttgctcaagatgtccagtcgcagatcacatc>5085      987<TGCCATCAAAGACACTGATTTCCAGAACCACCCTT-CTGGTTGTTTGGTTGGAAACAACAACACTAGCTTTGCTCAAGATGTCCAGTCGCAGATCACATC<889       988<TGCCATCAAAGACACTGATTTCCAGAACCACCCTTTCTGGTTGTTTGGTTGGAAACAACAACACTAGCTTTGCTCAAGATGTCCAGTCGCAGATCACATC<889       988<TGCCATCAAAGACACTGATTTCCAGAACCACCCTT-CTGGTTGTTTGGTTGGAAACAACAACACTAGCTTTGCTCAAGATGTCCAGTCGCAGATCACATC<890       989<TGCCATCAAAGACACTGATTTCCAGAACCACCCTTTCTGGTTGTTTGGTTGGAAACAACAACACTAGCTTTGCTCAAGATGTCCAGTCGCAGATCACATC<890      1044<TGCCATCAAAGACACTGATTTCCAGAACCACCCTT-CTGGTTGTTTGGTTGGAAACAACAACACTAGCTTTGCTCAAGATGTCCAGTCGCAGATCACATC<946      1046<TGCCATCAAAGACACTGATTTCCAGAACCACCTTT-CTGGTTGTTTGGTTGGAAACAACAACACTAGCTTTGCTCAAGATGTCCAGTCGCAGATCACATC<948               *         *         *         *         *         *         *         *         *         *       5086>agctagctttgcagactcacaggccttctctcgccaagattttccagataattctggaggcactggtacatcttcaagcaatgttgattttgatgattgt>5185      888<AGCTAGCTTTGCAGACTCACAGGCCTTCTCTCGCCAAGATTTTCCAGATAATTCTGGAGGCACTGGTACATCTTCAAGCAATGTTGATTTTGATGATTGT<789       888<AGCTAGCTTTGCAGACTCACAGGCCTTCTCTCGCCAAGATTTTCCAGATAATTCTGGAGGCACTGGTACATCTTCAAGCAATGTTGATTTTGATGATTGT<789       889<AGCTAGCTTTGCAGACTCACAGGCCTTCTCTCGCCAAGATTTTCCAGATAATTCTGGAGGCACTGGTACATCTTCAAGCAATGTTGATTTTGATGATTGT<790       889<AGCTAGCTTTGCAGACTCACAGGCCTTCTCTCGCCAAGATTTTCCAGATAATTCTGGAGGCACTGGTACATCTTCAAGCAATGTTGATTTTGATGATTGT<790       945<AGCTAGCTTTGCAGACTCACAGGCCTTCTCTCGCCAAGATTTTCCAGATAATTCTGGAGGCACTGGTACATCTTCAAGCAATGTTGATTTTGATGATTGT<846       947<AGCTAGCTTTGCATACTCACAGGCCTTCTCTCGCCAAGATTTTCCAGATAATTCTGGAGGCACTGGTACATCTTCAAGCAATGTTGATTTTGATGATTGT<848               *         *         *         *         *         *         *         *         *         *       5186>agtctgcggcaaaatagtaaaggctcatcatggcagaaaattgcgacaccccgcgtccgaaccggcagcgatctgggtaaaaagctgctggaagcagccg>5285      788<AGTCTGCGGCAAAATAGTAAAGGCTCATCATGGCAGAAAATTGCGACACCCCGCGTCCGAACCGGCAGCGATCTGGGTAAAAAGCTGCTGGAAGCAGCCG<689       788<AGTCTGCGGCAAAATAGTAAAGGCTCATCATGGCAGAAAATTGCGACACCCCGCGTCCGAACCGGCAGCGATCTGGGTAAAAAGCTGCTGGAAGCAGCCG<689       789<AGTCTGCGGCAAAATAGTAAAGGCTCATCATGGCAGAAAATTGCGACACCCCGCGTCCGAACCGGCAGCGATCTGGGTAAAAAGCTGCTGGAAGCAGCCG<690       789<AGTCTGCGGCAAAATAGTAAAGGCTCATCATGGCAGAAAATTGCGACACCCCGCGTCCGAACCGGCAGCGATCTGGGTAAAAAGCTGCTGGAAGCAGCCG<690       845<AGTCTGCGGCAAAATAGTAAAGGCTCATCATGGCAGAAAATTGCGACACCCCGCGTCCGAACCGGCAGCGATCTGGGTAAAAAGCTGCTGGAAGCAGCCG<746       847<AGTCTGCGGCAAAATAGTAAAGGCTCATCATGGCAGAAAATTGCGACACCCCGCGTCCGAACCGGCAGCGATCTGGGTAAAAAGCTGCTGGAAGCAGCCG<748               *         *         *         *         *         *         *         *         *                 5286>cggccggccaagatgatgaggtgcgtattctgatggcgaatggggccgatgttaacgcaaccgacgacgatggcctgactccgct--------------->5370      688<CGGCCGGCCAAGATGATGAGGTGCGTATTCTGATGGCGAATGGGGCCGATGTTAACGCAACCGACGACGATGGCCTGACTCCGCT---------------<604       688<CGGCCGGCCAAGATGATGAGGTGCGTATTCTGATGGCGAATGGGGCCGATGTTAACGCAACCGACGACAATGGCCTGACTCCGCT---------------<604       689<CGGCCGGCCAAGATGATGAGGTGCGTATTCTGATGGCGAATGGGGCCGATGTTAACGCAACCGACGACAATGGCCTGACTCCGCT---------------<605       689<CGGCCGGCCAAGATGATGAGGTGCGTATTCTGATGGCGAATGGGGCCGATGTTAACGCAACCGACGACGATGGCCTGACTCCGCT---------------<605       745<CGGCCGGCCAAGATGATGAGGTGCGTATTCTGATGGCGAATGGGGCCGATGTTAACGCAACCGACGACGATGGCCTGACTCCGCTGCACCTGGCGAATGG<646       747<CGGCCGGCCAAGATGATGAGGTGCGTATTCTGATGGCGAATGGGGCCGATGTTAACGCAACCGACGACGATGGCCTGACTCCGCTGCACCTGGCGGAATG<648                                                               *         *         *         *         *         5371>-------------------------------------------gcacctggcggctgcaaacgggcaactggaaatcgtagaggtactgctgaaaaatgg>5427      603<-------------------------------------------GCACCTGGCGGCTGCAAACGGGCAACTGGAAATCGTAGAGGTACTGCTGAAAAATGG<547       603<-------------------------------------------GCACCTGGCGGCTGCAAACGGGCAACTGGAAATCGTAGAGGTACTGCTGAAAAATGG<547       604<-------------------------------------------GCACCTGGCGGCTGCAAACGGGCAACTGGAAATCGTAGAGGTACTGCTGAAAAATGG<548       604<-------------------------------------------GCACCTGGCGGCTGCAAACGGGCAACTGGAAATCGTAGAGGTACTGCTGAAAAATGG<548       645<GGCCGATGTTAACGCAACCGACGACGATGGCCTGACTCCGCT-GCACCTGGCGGCTGCAAACGGGCAACTGGAAATCGTAGAGGTACTGCTGAAAAATGG<547       647<GGGCCGATGTTAACGCAACCGACGACGATGGCCTGACTCCGCTGCACCTGGCGGCTGCAAACGGGCAACTGGAAATCGTAGAGGTACTGCTGAAAAATGG<548             *         *         *         *         *         *         *         *         *         *         5428>cgccgatgtgaacgcttctgatagtgcgggtattactccgctgcacctggccgcttatgacggccatctggagattgtcgaagtcctgctgaagcacggg>5527      546<CGCCGATGTGAACGCTTCTGATAGTGCGGGTATTACTCCGCTGCACCTGGCCGCTTATGACGGCCATCTGGAGATTGTCGAAGTCCTGCTGAAGCACGGG<447       546<CGCCGATGTGAACGCTTCTGATAGTGCGGGTATTACTCCGCTGCACCTGGCCGCTTATGACGGCCATCTGGAGATTGTCGAAGTCCTGCTGAAGCACGGG<447       547<CGCCGATGTGAACGCTTCTGATAGTGCGGGTATTACTCCGCTGCACCTGGCCGCTTATGACGGCCATCTGGAGATTGTCGAAGTCCTGCTGAAGCACGGG<448       547<CGCCGATGTGAACGCTTCTGATAGTGCGGGTATTACTCCGCTGCACCTGGCCGCTTATGACGGCCATCTGGAGATTGTCGAAGTCCTGCTGAAGCACGGG<448       546<CGCCGATGTGAACGCTTCTGATAGTGCGGGTATTACTCCGCTGCACCTGGCCGCTTATGACGGCCATCTGGAGATTGTCGAAGTCCTGCTGAAGCACGGG<447       547<CGCCGATGTGAACGCTTCTGATAGTGCGGGTATTACTCCGCTGCACCTGGCCGCTTATGACGGCCATCTGGAGATTGTCGAAGTCCTGCTGAAGCACGGG<448             *         *         *         *         *         *         *         *         *         *         5528>gctgacgttaatgcgtacgaccgcgccgggtggacaccgctgcacctagcagcgctgagtggccaactggagattgtggaagttctgctgaaacacggcg>5627      446<GCTGACGTTAATGCGTACGACCGCGCCGGGTGGACACCGCTGCACCTAGCAGCGCTGAGTGGCCAACTGGAGATTGTGGAAGTTCTGCTGAAACACGGCG<347       446<GCTGACGTTAATGCGTACGACCGCGCCGGGTGGACACCGCTGCACCTAGCAGCGCTGAGTGGCCAACTGGAGATTGTGGAAGTTCTGCTGAAACACGGCG<347       447<GCTGACGTTAATGCGTACGACCGCGCCGGGTGGACACCGCTGCACCTAGCAGCGCTGAGTGGCCAACTGGAGATTGTGGAAGTTCTGCTGAAACACGGCG<348       447<GCTGACGTTAATGCGTACGACCGCGCCGGGTGGACACCGCTGCACCTAGCAGCGCTGAGTGGCCAACTGGAGATTGTGGAAGTTCTGCTGAAACACGGCG<348       446<GCTGACGTTAATGCGTACGACCGCGCCGGGTGGACACCGCTGCACCTAGCAGCGCTGAGTGGCCAACTGGAGATTGTGGAAGTTCTGCTGAAACACGGCG<347       447<GCTGACGTTAATGCGTACGACCGCGCCGGGTGGACACCGCTGCACCTAGCAGCGCTGAGTGGCCAACTGGAGATTGTGGAAGTTCTGCTGAAACACGGCG<348             *         *         *         *         *         *         *         *         *         *                                                                                                              H  H                                                                                                      CACca  5628>cagatgtcaacgcccaagacgcactgggcctgaccgcgtttgatatctcgattaatcaaggtcaggaagatctggcagagatcctgcaactcgagcacca>5727      346<CAGATGTCAACGCCCAAGACGCACTGGGCCTGACCGCGTTTGATATCTCGATTAATCAAGGTCAGGAAGATCTGGCAGAGATCCTGCAACTCGAGCACCA<247       346<CAGATGTCAACGCCCAAGACGCACTGGGCCTGACCGCGTTTGATATCTCGATTAATCAAGGTCAGGAAGATCTGGCAGAGATCCTGCAACTCGAGCACCA<247       347<CAGATGTCAACGCCCAAGACGCACTGGGCCTGACCGCGTTTGATATCTCGATTAATCAAGGTCAGGAAGATCTGGCAGAGATCCTGCAACTCGAGCACCA<248       347<CAGATGTCAACGCCCAAGACGCACTGGGCCTGACCGCGTTTGATATCTCGATTAATCAAGGTCAGGAAGATCTGGCAGAGATCCTGCAACTCGAGCACCA<248       346<CAGATGTCAACGCCCAAGACGCACTGGGCCTGACCGCGTTTGATATCTCGATTAATCAAGGTCAGGAAGATCTGGCAGAGATCCTGCAACTCGAGCACCA<247       347<CAGATGTCAACGCCCAAGACGCACTGGGCCTGACCGCGTTTGATATCTCGATTAATCAAGGTCAGGAAGATCTGGCAGAGATCCTGCAACTCGAGCACCA<248             *         *                                                                                                H  H  H  H      ----------------------------------------------------------------------------------       cCACcacCACcac     ----------------------------------------------------------------------------------  5728>ccaccaccaccactgaca---------------------------------------------------------------------------------->5745      246<CCACCACCACCACTGACACCCAGCTTTCTTGTCCACCACCACTGACACCCAGCTTTCTTGTCCACCACCACTGACACCCAGCTTTCTTGTCCACCACCAC<147       246<CCACCACCACCACTGACACCCAGCTTTCTTGTCCACCACCACTGACACCCAGCTTTCTTGTCCACCACCACTGACACCCAGCTTTCTTGTCCACCACCAC<147       247<CCACCACCACCACTGACACCCAGCTTTCTTGTCCACCACCACTGACACCCAGCTTTCTTGTCCACCACCACTGACACCCAGCTTTCTTGTCCACCACCAC<148       247<CCACCACCACCACTGACACCCAGCTTTCTTGTCCACCACCACTGACACCCAGCTTTCTTGTCCACCACCACTGACACCCAGCTTTCTTGTCCACCACCAC<148       246<CCACCACCACCACTGACACCCAGCTTTCTTGTCCACCACCACTGACACCCAGCTTTCTTGTCCACCACCACTGACACCCAGCTTTCTTGTCCACCACCAC<147       247<CCACCACCACCACTGACACCCAGCTTTCTTGTCCACCACCACTGACACCCAGCTTTCTTGTCCACCACCACTGACACCCAGCTTTCTTGTCCACCACCAC<148                                                                              *         *         *         *    5746>---------------------------------------------------------------cccagctttcttgtacaaagtggttcttgtacaaagt>5782      146<TGACACCCAGCTTTCTTGTCCACCACCACTGACACCCAGCTTTCTTGTCCACCACCACTGACACCCAGCTTTCTTGT----------------ACAAAGT<63        146<TGACACCCAGCTTTCTTGTCCACCACCACTGACACCCAGCTTTCTTGTCCACCACCACTGACACCCAGCTTTCTTGT----------------ACAAAGT<63        147<TGACACCCAGCTTTCTTGTCCACCACCACTGACACCCAGCTTTCTTGTCCACCACCACTGACACCCAGCTTTCTTGT----------------ACAAAGT<64        147<TGACACCCAGCTTTCTTGTCCACCACCACTGACACCCAGCTTTCTTGTCCACCACCACTGACACCCAGCTTTCTTGT----------------ACAAAGT<64        146<TGACACCCAGCTTTCTTGTCCACCACCACTGACACCCAGCTTTCTTGTCCACCACCACTGACACCCAGCTTTCTTGT----------------ACAAAGT<63        147<TGACACCCAGCTTTCTTGTCCACCACCACTGACACCCAGCTTTCTTGTCCACCACCACTGACACCCAGCTTTCTTGT----------------ACAAAGT<64                   *         *         *         *         *         *         *         *         *         *    5783>ggtgatgggctgcaggaattcgatatcaagcttatcgataccgtcgacctcgagtcatgtaattagttatgtcacgcttacattcacgccctccccccac>5882       62<GGTGATGGGCTGCAGGAATTCGATTTCAAGCTTATCGATACCGTCGACCTCGAGTCA-------------------------------------------<6          62<GGTGATGGGCTGCAGGAATTCGATTTCAAGCTTATCGATACCGTCGACCTCGAGTCA-------------------------------------------<6          63<GGTGATGGGCTGCAGGAATTCGATTTCAAGCTTATCGATACCGTCGACCTCGAGTCA-------------------------------------------<7          63<GGTGATGGGCTGCAGGAATTCGATTTCAAGCTTATCGATACCGTCGACCTCGAGTCA-------------------------------------------<7          62<GGTGATGGGCTGCAGGAATTCGAT-TCAAGCTTATCGATACCGTCGACCTCGAGTCA-------------------------------------------<7          63<GGTGATGGGCTGCAGGAATTCGATTTCAAGCTTATCGATACCGTCGACCTCGAGTCA-------------------------------------------<7                    *         *         *         *         *         *         *         *         *         *    5883>atccgctctaaccgaaaaggaaggagttagacaacctgaagtctaggtccctatttatttttttatagttatgttagtattaagaacgttatttatattt>5982        6<----------------------------------------------------------------------------------------------------<6           6<----------------------------------------------------------------------------------------------------<6           7<----------------------------------------------------------------------------------------------------<7           7<----------------------------------------------------------------------------------------------------<7           7<----------------------------------------------------------------------------------------------------<7           7<----------------------------------------------------------------------------------------------------<7                    *         *         *         *         *         *         *         *         *         *    5983>caaatttttcttttttttctgtacagacgcgtgtacgcatgtaacattatactgaaaaccttgcttgagaaggttttgggacgctcgaaggctttaattt>6082        6<----------------------------------------------------------------------------------------------------<6           6<----------------------------------------------------------------------------------------------------<6           7<----------------------------------------------------------------------------------------------------<7           7<----------------------------------------------------------------------------------------------------<7           7<----------------------------------------------------------------------------------------------------<7           7<----------------------------------------------------------------------------------------------------<7                    *         *         *         *         *         *         *         *         *         *    6083>gtgacaccgattatttaaagctgcagcatacgatatatatacatgtgtatatatgtatacctatgaatgtcagtaagtatgtatacgaacagtatgatac>6182        5<-------------------------------------------------------------------------------------------GTATG~~~~<1           6<----------------------------------------------------------------------------------------------------<6           7<----------------------------------------------------------------------------------------------------<7           7<----------------------------------------------------------------------------------------------------<7           6<------------------------------------------------------------------------GTAAGT~~~~~~~~~~~~~~~~~~~~~~<1           7<----------------------------------------------------------------------------------------------------<7                    *         *         *         *         *         *         *         *         *         *    6183>tgaagatgacaaggtaatgcatcattctatacgtgtcattctgaacgaggcgcgctttccttttttctttttgctttttctttttttttctcttgaactc>6282        1<~~~~~~~~~~~~~~~~~~~~~~~~~~~~~~~~~~~~~~~~~~~~~~~~~~~~~~~~~~~~~~~~~~~~~~~~~~~~~~~~~~~~~~~~~~~~~~~~~~~~<1           6<----------------------------------------------------------------------------------------------------<6           7<----------------------------------------------------------------------------------------------------<7           7<----------------------------------------------------------------------------------------------------<7           1<~~~~~~~~~~~~~~~~~~~~~~~~~~~~~~~~~~~~~~~~~~~~~~~~~~~~~~~~~~~~~~~~~~~~~~~~~~~~~~~~~~~~~~~~~~~~~~~~~~~~<1           7<----------------------------------------------------------------------------------------------------<7                    *         *         *         *         *         *         *         *         *         *    6283>gagaaaaaaaatataaaagagatggaggaacgggaaaaagttagttgtggtgataggtggcaagtggtattccgtaagaacaacaagaaaagcatttcat>6382        1<~~~~~~~~~~~~~~~~~~~~~~~~~~~~~~~~~~~~~~~~~~~~~~~~~~~~~~~~~~~~~~~~~~~~~~~~~~~~~~~~~~~~~~~~~~~~~~~~~~~~<1           5<-------------------------------------------------------------------------GTAAG~~~~~~~~~~~~~~~~~~~~~~<1           7<----------------------------------------------------------------------------------------------------<7           7<----------------------------------------------------------------------------------------------------<7           1<~~~~~~~~~~~~~~~~~~~~~~~~~~~~~~~~~~~~~~~~~~~~~~~~~~~~~~~~~~~~~~~~~~~~~~~~~~~~~~~~~~~~~~~~~~~~~~~~~~~~<1           7<----------------------------------------------------------------------------------------------------<7                    *         *         *         *         *         *         *         *         *         *    6383>attatggctgaactgagcgaacaagtgcaaaatttaagcatcaacgacaacaacgagaatggttatgttcctcctcacttaagaggaaaaccaagaagtg>6482        1<~~~~~~~~~~~~~~~~~~~~~~~~~~~~~~~~~~~~~~~~~~~~~~~~~~~~~~~~~~~~~~~~~~~~~~~~~~~~~~~~~~~~~~~~~~~~~~~~~~~~<1           1<~~~~~~~~~~~~~~~~~~~~~~~~~~~~~~~~~~~~~~~~~~~~~~~~~~~~~~~~~~~~~~~~~~~~~~~~~~~~~~~~~~~~~~~~~~~~~~~~~~~~<1           7<----------------------------------------------------------------------------------------------------<7           7<----------------------------------------------------------------------------------------------------<7           1<~~~~~~~~~~~~~~~~~~~~~~~~~~~~~~~~~~~~~~~~~~~~~~~~~~~~~~~~~~~~~~~~~~~~~~~~~~~~~~~~~~~~~~~~~~~~~~~~~~~~<1           7<----------------------------------------------------------------------------------------------------<7                    *         *         *         *         *         *         *         *         *         *    6483>ccagaaataacagtagcaactacaataacaacaacggcggctacaacggtggccgtggcggtggcagcttctttagcaacaaccgtcgtggtggttacgg>6582        1<~~~~~~~~~~~~~~~~~~~~~~~~~~~~~~~~~~~~~~~~~~~~~~~~~~~~~~~~~~~~~~~~~~~~~~~~~~~~~~~~~~~~~~~~~~~~~~~~~~~~<1           1<~~~~~~~~~~~~~~~~~~~~~~~~~~~~~~~~~~~~~~~~~~~~~~~~~~~~~~~~~~~~~~~~~~~~~~~~~~~~~~~~~~~~~~~~~~~~~~~~~~~~<1           7<----------------------------------------------------------------------------------------------------<7           7<----------------------------------------------------------------------------------------------------<7           1<~~~~~~~~~~~~~~~~~~~~~~~~~~~~~~~~~~~~~~~~~~~~~~~~~~~~~~~~~~~~~~~~~~~~~~~~~~~~~~~~~~~~~~~~~~~~~~~~~~~~<1           7<----------------------------------------------------------------------------------------------------<7                    *         *         *         *         *         *         *         *         *         *    6583>caacggtggtttcttcggtggaaacaacggtggcagcagatctaacggccgttctggtggtagatggatcgatggcaaacatgtcccagctccaagaaac>6682        1<~~~~~~~~~~~~~~~~~~~~~~~~~~~~~~~~~~~~~~~~~~~~~~~~~~~~~~~~~~~~~~~~~~~~~~~~~~~~~~~~~~~~~~~~~~~~~~~~~~~~<1           1<~~~~~~~~~~~~~~~~~~~~~~~~~~~~~~~~~~~~~~~~~~~~~~~~~~~~~~~~~~~~~~~~~~~~~~~~~~~~~~~~~~~~~~~~~~~~~~~~~~~~<1           7<----------------------------------------------------------------------------------------------------<7           7<----------------------------------------------------------------------------------------------------<7           1<~~~~~~~~~~~~~~~~~~~~~~~~~~~~~~~~~~~~~~~~~~~~~~~~~~~~~~~~~~~~~~~~~~~~~~~~~~~~~~~~~~~~~~~~~~~~~~~~~~~~<1           7<----------------------------------------------------------------------------------------------------<7                    *         *         *         *         *         *         *         *         *         *    6683>gaaaaggccgagatcgccatatttggtgtccccgaggatccaaatttccaatcttctggtattaacttcgataactacgatgatattccagtggacgcct>6782        1<~~~~~~~~~~~~~~~~~~~~~~~~~~~~~~~~~~~~~~~~~~~~~~~~~~~~~~~~~~~~~~~~~~~~~~~~~~~~~~~~~~~~~~~~~~~~~~~~~~~~<1           1<~~~~~~~~~~~~~~~~~~~~~~~~~~~~~~~~~~~~~~~~~~~~~~~~~~~~~~~~~~~~~~~~~~~~~~~~~~~~~~~~~~~~~~~~~~~~~~~~~~~~<1           7<----------------------------------------------------------------------------------------------------<7           7<----------------------------------------------------------------------------------------------------<7           1<~~~~~~~~~~~~~~~~~~~~~~~~~~~~~~~~~~~~~~~~~~~~~~~~~~~~~~~~~~~~~~~~~~~~~~~~~~~~~~~~~~~~~~~~~~~~~~~~~~~~<1           7<----------------------------------------------------------------------------------------------------<7                    *         *         *         *         *         *         *         *         *         *    6783>ctggtaaggatgttcctgaaccaatcacagaatttacctcacctccattggacggattgttattggaaaacatcaaattggcccgtttcaccaagccaac>6882        1<~~~~~~~~~~~~~~~~~~~~~~~~~~~~~~~~~~~~~~~~~~~~~~~~~~~~~~~~~~~~~~~~~~~~~~~~~~~~~~~~~~~~~~~~~~~~~~~~~~~~<1           1<~~~~~~~~~~~~~~~~~~~~~~~~~~~~~~~~~~~~~~~~~~~~~~~~~~~~~~~~~~~~~~~~~~~~~~~~~~~~~~~~~~~~~~~~~~~~~~~~~~~~<1           6<---GTAAGG~~~~~~~~~~~~~~~~~~~~~~~~~~~~~~~~~~~~~~~~~~~~~~~~~~~~~~~~~~~~~~~~~~~~~~~~~~~~~~~~~~~~~~~~~~~<1           7<----------------------------------------------------------------------------------------------------<7           1<~~~~~~~~~~~~~~~~~~~~~~~~~~~~~~~~~~~~~~~~~~~~~~~~~~~~~~~~~~~~~~~~~~~~~~~~~~~~~~~~~~~~~~~~~~~~~~~~~~~~<1           7<----------------------------------------------------------------------------------------------------<7                    *         *         *         *         *         *         *         *         *         *    6883>acctgtgcaaaaatactccgtccctatcgttgccaacggcagagatttgatggcctgtgcgcagaccggttctggtaagactggtgggtttttattccca>6982        1<~~~~~~~~~~~~~~~~~~~~~~~~~~~~~~~~~~~~~~~~~~~~~~~~~~~~~~~~~~~~~~~~~~~~~~~~~~~~~~~~~~~~~~~~~~~~~~~~~~~~<1           1<~~~~~~~~~~~~~~~~~~~~~~~~~~~~~~~~~~~~~~~~~~~~~~~~~~~~~~~~~~~~~~~~~~~~~~~~~~~~~~~~~~~~~~~~~~~~~~~~~~~~<1           1<~~~~~~~~~~~~~~~~~~~~~~~~~~~~~~~~~~~~~~~~~~~~~~~~~~~~~~~~~~~~~~~~~~~~~~~~~~~~~~~~~~~~~~~~~~~~~~~~~~~~<1           7<----------------------------------------------------------------------------------------------------<7           1<~~~~~~~~~~~~~~~~~~~~~~~~~~~~~~~~~~~~~~~~~~~~~~~~~~~~~~~~~~~~~~~~~~~~~~~~~~~~~~~~~~~~~~~~~~~~~~~~~~~~<1           7<----------------------------------------------------------------------------------------------------<7                    *         *         *         *         *         *         *         *         *         *    6983>gtgttgtccgaatcatttaagactggaccatctcctcaaccagagtctcaaggctccttttaccaaagaaaggcctacccaactgctgtcattatggctc>7082        1<~~~~~~~~~~~~~~~~~~~~~~~~~~~~~~~~~~~~~~~~~~~~~~~~~~~~~~~~~~~~~~~~~~~~~~~~~~~~~~~~~~~~~~~~~~~~~~~~~~~~<1           1<~~~~~~~~~~~~~~~~~~~~~~~~~~~~~~~~~~~~~~~~~~~~~~~~~~~~~~~~~~~~~~~~~~~~~~~~~~~~~~~~~~~~~~~~~~~~~~~~~~~~<1           1<~~~~~~~~~~~~~~~~~~~~~~~~~~~~~~~~~~~~~~~~~~~~~~~~~~~~~~~~~~~~~~~~~~~~~~~~~~~~~~~~~~~~~~~~~~~~~~~~~~~~<1           7<----------------------------------------------------------------------------------------------------<7           1<~~~~~~~~~~~~~~~~~~~~~~~~~~~~~~~~~~~~~~~~~~~~~~~~~~~~~~~~~~~~~~~~~~~~~~~~~~~~~~~~~~~~~~~~~~~~~~~~~~~~<1           7<----------------------------------------------------------------------------------------------------<7                    *         *         *         *         *         *         *         *         *         *    7083>cagtttaaaccatggtcatagctgtttcctgtgtgaaattgttatccgctcacaattccacacaacataggagccggaagcataaagtgtaaagcctggg>7182        1<~~~~~~~~~~~~~~~~~~~~~~~~~~~~~~~~~~~~~~~~~~~~~~~~~~~~~~~~~~~~~~~~~~~~~~~~~~~~~~~~~~~~~~~~~~~~~~~~~~~~<1           1<~~~~~~~~~~~~~~~~~~~~~~~~~~~~~~~~~~~~~~~~~~~~~~~~~~~~~~~~~~~~~~~~~~~~~~~~~~~~~~~~~~~~~~~~~~~~~~~~~~~~<1           1<~~~~~~~~~~~~~~~~~~~~~~~~~~~~~~~~~~~~~~~~~~~~~~~~~~~~~~~~~~~~~~~~~~~~~~~~~~~~~~~~~~~~~~~~~~~~~~~~~~~~<1           7<----------------------------------------------------------------------------------------------------<7           1<~~~~~~~~~~~~~~~~~~~~~~~~~~~~~~~~~~~~~~~~~~~~~~~~~~~~~~~~~~~~~~~~~~~~~~~~~~~~~~~~~~~~~~~~~~~~~~~~~~~~<1           7<----------------------------------------------------------------------------------------------------<7                    *         *         *         *         *         *         *         *         *         *    7183>gtgcctaatgagtgaggtaactcacattaattgcgttgcgctcactgcccgctttccagtcgggaaacctgtcgtgccagctgcattaatgaatcggcca>7282        1<~~~~~~~~~~~~~~~~~~~~~~~~~~~~~~~~~~~~~~~~~~~~~~~~~~~~~~~~~~~~~~~~~~~~~~~~~~~~~~~~~~~~~~~~~~~~~~~~~~~~<1           1<~~~~~~~~~~~~~~~~~~~~~~~~~~~~~~~~~~~~~~~~~~~~~~~~~~~~~~~~~~~~~~~~~~~~~~~~~~~~~~~~~~~~~~~~~~~~~~~~~~~~<1           1<~~~~~~~~~~~~~~~~~~~~~~~~~~~~~~~~~~~~~~~~~~~~~~~~~~~~~~~~~~~~~~~~~~~~~~~~~~~~~~~~~~~~~~~~~~~~~~~~~~~~<1           7<----------------------------------------------------------------------------------------------------<7           1<~~~~~~~~~~~~~~~~~~~~~~~~~~~~~~~~~~~~~~~~~~~~~~~~~~~~~~~~~~~~~~~~~~~~~~~~~~~~~~~~~~~~~~~~~~~~~~~~~~~~<1           7<----------------------------------------------------------------------------------------------------<7                    *         *         *         *         *         *         *         *         *         *    7283>acgcgcggggagaggcggtttgcgtattgggcgctcttccgcttcctcgctcactgactcgctgcgctcggtcgttcggctgcggcgagcggtatcagct>7382        1<~~~~~~~~~~~~~~~~~~~~~~~~~~~~~~~~~~~~~~~~~~~~~~~~~~~~~~~~~~~~~~~~~~~~~~~~~~~~~~~~~~~~~~~~~~~~~~~~~~~~<1           1<~~~~~~~~~~~~~~~~~~~~~~~~~~~~~~~~~~~~~~~~~~~~~~~~~~~~~~~~~~~~~~~~~~~~~~~~~~~~~~~~~~~~~~~~~~~~~~~~~~~~<1           1<~~~~~~~~~~~~~~~~~~~~~~~~~~~~~~~~~~~~~~~~~~~~~~~~~~~~~~~~~~~~~~~~~~~~~~~~~~~~~~~~~~~~~~~~~~~~~~~~~~~~<1           7<----------------------------------------------------------------------------------------------------<7           1<~~~~~~~~~~~~~~~~~~~~~~~~~~~~~~~~~~~~~~~~~~~~~~~~~~~~~~~~~~~~~~~~~~~~~~~~~~~~~~~~~~~~~~~~~~~~~~~~~~~~<1           7<----------------------------------------------------------------------------------------------------<7                    *         *         *         *         *         *         *         *         *         *    7383>cactcaaaggcggtaatacggttatccacagaatcaggggataacgcaggaaagaacatgtgagcaaaaggccagcaaaaggccaggaaccgtaaaaagg>7482        1<~~~~~~~~~~~~~~~~~~~~~~~~~~~~~~~~~~~~~~~~~~~~~~~~~~~~~~~~~~~~~~~~~~~~~~~~~~~~~~~~~~~~~~~~~~~~~~~~~~~~<1           1<~~~~~~~~~~~~~~~~~~~~~~~~~~~~~~~~~~~~~~~~~~~~~~~~~~~~~~~~~~~~~~~~~~~~~~~~~~~~~~~~~~~~~~~~~~~~~~~~~~~~<1           1<~~~~~~~~~~~~~~~~~~~~~~~~~~~~~~~~~~~~~~~~~~~~~~~~~~~~~~~~~~~~~~~~~~~~~~~~~~~~~~~~~~~~~~~~~~~~~~~~~~~~<1           7<----------------------------------------------------------------------------------------------------<7           1<~~~~~~~~~~~~~~~~~~~~~~~~~~~~~~~~~~~~~~~~~~~~~~~~~~~~~~~~~~~~~~~~~~~~~~~~~~~~~~~~~~~~~~~~~~~~~~~~~~~~<1           7<----------------------------------------------------------------------------------------------------<7                    *         *         *         *         *         *         *         *         *         *    7483>ccgcgttgctggcgtttttccataggctcggcccccctgacgagcatcacaaaaatcgacgctcaagtcagaggtggcgaaacccgacaggactataaag>7582        1<~~~~~~~~~~~~~~~~~~~~~~~~~~~~~~~~~~~~~~~~~~~~~~~~~~~~~~~~~~~~~~~~~~~~~~~~~~~~~~~~~~~~~~~~~~~~~~~~~~~~<1           1<~~~~~~~~~~~~~~~~~~~~~~~~~~~~~~~~~~~~~~~~~~~~~~~~~~~~~~~~~~~~~~~~~~~~~~~~~~~~~~~~~~~~~~~~~~~~~~~~~~~~<1           1<~~~~~~~~~~~~~~~~~~~~~~~~~~~~~~~~~~~~~~~~~~~~~~~~~~~~~~~~~~~~~~~~~~~~~~~~~~~~~~~~~~~~~~~~~~~~~~~~~~~~<1           7<----------------------------------------------------------------------------------------------------<7           1<~~~~~~~~~~~~~~~~~~~~~~~~~~~~~~~~~~~~~~~~~~~~~~~~~~~~~~~~~~~~~~~~~~~~~~~~~~~~~~~~~~~~~~~~~~~~~~~~~~~~<1           7<----------------------------------------------------------------------------------------------------<7                    *         *         *         *         *         *         *         *         *         *    7583>ataccaggcgttcccccctggaagctccctcgtgcgctctcctgttccgaccctgccgcttaccggatacctgtccgcctttctcccttcgggaagcgtg>7682        1<~~~~~~~~~~~~~~~~~~~~~~~~~~~~~~~~~~~~~~~~~~~~~~~~~~~~~~~~~~~~~~~~~~~~~~~~~~~~~~~~~~~~~~~~~~~~~~~~~~~~<1           1<~~~~~~~~~~~~~~~~~~~~~~~~~~~~~~~~~~~~~~~~~~~~~~~~~~~~~~~~~~~~~~~~~~~~~~~~~~~~~~~~~~~~~~~~~~~~~~~~~~~~<1           1<~~~~~~~~~~~~~~~~~~~~~~~~~~~~~~~~~~~~~~~~~~~~~~~~~~~~~~~~~~~~~~~~~~~~~~~~~~~~~~~~~~~~~~~~~~~~~~~~~~~~<1           7<----------------------------------------------------------------------------------------------------<7           1<~~~~~~~~~~~~~~~~~~~~~~~~~~~~~~~~~~~~~~~~~~~~~~~~~~~~~~~~~~~~~~~~~~~~~~~~~~~~~~~~~~~~~~~~~~~~~~~~~~~~<1           7<----------------------------------------------------------------------------------------------------<7                    *         *         *         *         *         *         *         *         *         *    7683>gcgctttctcaatgctcacgctgtaggtatctcagttcggtgtaggtcgttcgctccaagctgggctgtgtgcacgaaccccccgttcagcccgaccgct>7782        1<~~~~~~~~~~~~~~~~~~~~~~~~~~~~~~~~~~~~~~~~~~~~~~~~~~~~~~~~~~~~~~~~~~~~~~~~~~~~~~~~~~~~~~~~~~~~~~~~~~~~<1           1<~~~~~~~~~~~~~~~~~~~~~~~~~~~~~~~~~~~~~~~~~~~~~~~~~~~~~~~~~~~~~~~~~~~~~~~~~~~~~~~~~~~~~~~~~~~~~~~~~~~~<1           1<~~~~~~~~~~~~~~~~~~~~~~~~~~~~~~~~~~~~~~~~~~~~~~~~~~~~~~~~~~~~~~~~~~~~~~~~~~~~~~~~~~~~~~~~~~~~~~~~~~~~<1           7<----------------------------------------------------------------------------------------------------<7           1<~~~~~~~~~~~~~~~~~~~~~~~~~~~~~~~~~~~~~~~~~~~~~~~~~~~~~~~~~~~~~~~~~~~~~~~~~~~~~~~~~~~~~~~~~~~~~~~~~~~~<1           7<----------------------------------------------------------------------------------------------------<7                    *         *         *         *         *         *         *         *         *         *    7783>gcgccttatccggtaactatcgtcttgagtccaacccggtaagacacgacttatcgccactggcagcagccactggtaacaggattagcagagcgaggta>7882        1<~~~~~~~~~~~~~~~~~~~~~~~~~~~~~~~~~~~~~~~~~~~~~~~~~~~~~~~~~~~~~~~~~~~~~~~~~~~~~~~~~~~~~~~~~~~~~~~~~~~~<1           1<~~~~~~~~~~~~~~~~~~~~~~~~~~~~~~~~~~~~~~~~~~~~~~~~~~~~~~~~~~~~~~~~~~~~~~~~~~~~~~~~~~~~~~~~~~~~~~~~~~~~<1           1<~~~~~~~~~~~~~~~~~~~~~~~~~~~~~~~~~~~~~~~~~~~~~~~~~~~~~~~~~~~~~~~~~~~~~~~~~~~~~~~~~~~~~~~~~~~~~~~~~~~~<1           7<----------------------------------------------------------------------------------------------------<7           1<~~~~~~~~~~~~~~~~~~~~~~~~~~~~~~~~~~~~~~~~~~~~~~~~~~~~~~~~~~~~~~~~~~~~~~~~~~~~~~~~~~~~~~~~~~~~~~~~~~~~<1           7<----------------------------------------------------------------------------------------------------<7                    *         *         *         *         *         *         *         *         *         *    7883>tgtaggcggtgctacagagttcttgaagtggtggcctaactacggctacactagaaggacagtatttggtatctgcgctctgctgaagccagttaccttc>7982        1<~~~~~~~~~~~~~~~~~~~~~~~~~~~~~~~~~~~~~~~~~~~~~~~~~~~~~~~~~~~~~~~~~~~~~~~~~~~~~~~~~~~~~~~~~~~~~~~~~~~~<1           1<~~~~~~~~~~~~~~~~~~~~~~~~~~~~~~~~~~~~~~~~~~~~~~~~~~~~~~~~~~~~~~~~~~~~~~~~~~~~~~~~~~~~~~~~~~~~~~~~~~~~<1           1<~~~~~~~~~~~~~~~~~~~~~~~~~~~~~~~~~~~~~~~~~~~~~~~~~~~~~~~~~~~~~~~~~~~~~~~~~~~~~~~~~~~~~~~~~~~~~~~~~~~~<1           7<----------------------------------------------------------------------------------------------------<7           1<~~~~~~~~~~~~~~~~~~~~~~~~~~~~~~~~~~~~~~~~~~~~~~~~~~~~~~~~~~~~~~~~~~~~~~~~~~~~~~~~~~~~~~~~~~~~~~~~~~~~<1           7<----------------------------------------------------------------------------------------------------<7                    *         *         *         *         *         *         *         *         *         *    7983>ggaaaaagagttggtagctcttgatccggcaaacaaaccaccgctggtagcggtggtttttttgtttgcaagcagcagattacgcgcagaaaaaaaggat>8082        1<~~~~~~~~~~~~~~~~~~~~~~~~~~~~~~~~~~~~~~~~~~~~~~~~~~~~~~~~~~~~~~~~~~~~~~~~~~~~~~~~~~~~~~~~~~~~~~~~~~~~<1           1<~~~~~~~~~~~~~~~~~~~~~~~~~~~~~~~~~~~~~~~~~~~~~~~~~~~~~~~~~~~~~~~~~~~~~~~~~~~~~~~~~~~~~~~~~~~~~~~~~~~~<1           1<~~~~~~~~~~~~~~~~~~~~~~~~~~~~~~~~~~~~~~~~~~~~~~~~~~~~~~~~~~~~~~~~~~~~~~~~~~~~~~~~~~~~~~~~~~~~~~~~~~~~<1           7<----------------------------------------------------------------------------------------------------<7           1<~~~~~~~~~~~~~~~~~~~~~~~~~~~~~~~~~~~~~~~~~~~~~~~~~~~~~~~~~~~~~~~~~~~~~~~~~~~~~~~~~~~~~~~~~~~~~~~~~~~~<1           7<----------------------------------------------------------------------------------------------------<7                    *         *         *         *         *         *         *         *         *         *    8083>ctcaagaagatcctttgatcttttctacggggtctgacgctcagtggaacgaaaactcacgttaagggattttggtcatgagattatcaaaaaggatctt>8182        1<~~~~~~~~~~~~~~~~~~~~~~~~~~~~~~~~~~~~~~~~~~~~~~~~~~~~~~~~~~~~~~~~~~~~~~~~~~~~~~~~~~~~~~~~~~~~~~~~~~~~<1           1<~~~~~~~~~~~~~~~~~~~~~~~~~~~~~~~~~~~~~~~~~~~~~~~~~~~~~~~~~~~~~~~~~~~~~~~~~~~~~~~~~~~~~~~~~~~~~~~~~~~~<1           1<~~~~~~~~~~~~~~~~~~~~~~~~~~~~~~~~~~~~~~~~~~~~~~~~~~~~~~~~~~~~~~~~~~~~~~~~~~~~~~~~~~~~~~~~~~~~~~~~~~~~<1           7<----------------------------------------------------------------------------------------------------<7           1<~~~~~~~~~~~~~~~~~~~~~~~~~~~~~~~~~~~~~~~~~~~~~~~~~~~~~~~~~~~~~~~~~~~~~~~~~~~~~~~~~~~~~~~~~~~~~~~~~~~~<1           7<----------------------------------------------------------------------------------------------------<7                    *         *         *         *         *         *         *         *         *         *                                                                                     *  W  H  K  I  L  S  A                                                                                    TTAccaATGcttAATcagTGAggcA  8183>cacctagatccttttaaattaaaaatgaagttttaaatcaatctaaagtatatatgagtaaacttggtctgacagttaccaatgcttaatcagtgaggca>8282        1<~~~~~~~~~~~~~~~~~~~~~~~~~~~~~~~~~~~~~~~~~~~~~~~~~~~~~~~~~~~~~~~~~~~~~~~~~~~~~~~~~~~~~~~~~~~~~~~~~~~~<1           1<~~~~~~~~~~~~~~~~~~~~~~~~~~~~~~~~~~~~~~~~~~~~~~~~~~~~~~~~~~~~~~~~~~~~~~~~~~~~~~~~~~~~~~~~~~~~~~~~~~~~<1           1<~~~~~~~~~~~~~~~~~~~~~~~~~~~~~~~~~~~~~~~~~~~~~~~~~~~~~~~~~~~~~~~~~~~~~~~~~~~~~~~~~~~~~~~~~~~~~~~~~~~~<1           7<----------------------------------------------------------------------------------------------------<7           1<~~~~~~~~~~~~~~~~~~~~~~~~~~~~~~~~~~~~~~~~~~~~~~~~~~~~~~~~~~~~~~~~~~~~~~~~~~~~~~~~~~~~~~~~~~~~~~~~~~~~<1           7<----------------------------------------------------------------------------------------------------<7                    *         *         *         *         *         *         *         *         *         *         G  I  E  A  I  Q  R  N  R  E  D  M  T  A  Q  S  G  T  T  Y  I  V  V  I  R  S  P  K  G  D  P  G  L  A       CCtatCTCagcGATctgTCTattTCGttcATCcatAGTtgcCTGactGCCcgtCGTgtaGATaacTACgatACGggaGGGcttACCatcTGGcccCAGtg  8283>cctatctcagcgatctgtctatttcgttcatccatagttgcctgactgcccgtcgtgtagataactacgatacgggagggcttaccatctggccccagtg>8382        1<~~~~~~~~~~~~~~~~~~~~~~~~~~~~~~~~~~~~~~~~~~~~~~~~~~~~~~~~~~~~~~~~~~~~~~~~~~~~~~~~~~~~~~~~~~~~~~~~~~~~<1           1<~~~~~~~~~~~~~~~~~~~~~~~~~~~~~~~~~~~~~~~~~~~~~~~~~~~~~~~~~~~~~~~~~~~~~~~~~~~~~~~~~~~~~~~~~~~~~~~~~~~~<1           1<~~~~~~~~~~~~~~~~~~~~~~~~~~~~~~~~~~~~~~~~~~~~~~~~~~~~~~~~~~~~~~~~~~~~~~~~~~~~~~~~~~~~~~~~~~~~~~~~~~~~<1           7<----------------------------------------------------------------------------------------------------<7           1<~~~~~~~~~~~~~~~~~~~~~~~~~~~~~~~~~~~~~~~~~~~~~~~~~~~~~~~~~~~~~~~~~~~~~~~~~~~~~~~~~~~~~~~~~~~~~~~~~~~~<1           7<----------------------------------------------------------------------------------------------------<7                    *         *         *         *         *         *         *         *         *         *           A  I  I  G  R  S  G  R  E  G  A  G  S  K  D  A  I  F  W  G  A  P  L  A  S  R  L  L  P  G  A  V  K        cTGCaatGATaccGCGagaCCCacgCTCaccGGCtccAGAtttATCagcAATaaaCCAgccAGCcggAAGggcCGAgcgCAGaagTGGtccTGCaacTTT  8383>ctgcaatgataccgcgagacccacgctcaccggctccagatttatcagcaataaaccagccagccggaagggccgagcgcagaagtggtcctgcaacttt>8482        1<~~~~~~~~~~~~~~~~~~~~~~~~~~~~~~~~~~~~~~~~~~~~~~~~~~~~~~~~~~~~~~~~~~~~~~~~~~~~~~~~~~~~~~~~~~~~~~~~~~~~<1           1<~~~~~~~~~~~~~~~~~~~~~~~~~~~~~~~~~~~~~~~~~~~~~~~~~~~~~~~~~~~~~~~~~~~~~~~~~~~~~~~~~~~~~~~~~~~~~~~~~~~~<1           1<~~~~~~~~~~~~~~~~~~~~~~~~~~~~~~~~~~~~~~~~~~~~~~~~~~~~~~~~~~~~~~~~~~~~~~~~~~~~~~~~~~~~~~~~~~~~~~~~~~~~<1           7<----------------------------------------------------------------------------------------------------<7           1<~~~~~~~~~~~~~~~~~~~~~~~~~~~~~~~~~~~~~~~~~~~~~~~~~~~~~~~~~~~~~~~~~~~~~~~~~~~~~~~~~~~~~~~~~~~~~~~~~~~~<1           7<----------------------------------------------------------------------------------------------------<7                    *         *         *         *         *         *         *         *         *         *          D  A  E  M  W  D  I  L  Q  Q  R  S  A  L  T  L  L  E  G  T  L  L  K  R  L  T  T  A  M  A  V  P  M         atcCGCctcCATccaGTCtatTAAttgTTGccgGGAagcTAGagtAAGtagTTCgccAGTtaaTAGtttGCGcaaCGTtgtTGCcatTGCtacAGGcatC  8483>atccgcctccatccagtctattaattgttgccgggaagctagagtaagtagttcgccagttaatagtttgcgcaacgttgttgccattgctacaggcatc>8582        1<~~~~~~~~~~~~~~~~~~~~~~~~~~~~~~~~~~~~~~~~~~~~~~~~~~~~~~~~~~~~~~~~~~~~~~~~~~~~~~~~~~~~~~~~~~~~~~~~~~~~<1           1<~~~~~~~~~~~~~~~~~~~~~~~~~~~~~~~~~~~~~~~~~~~~~~~~~~~~~~~~~~~~~~~~~~~~~~~~~~~~~~~~~~~~~~~~~~~~~~~~~~~~<1           1<~~~~~~~~~~~~~~~~~~~~~~~~~~~~~~~~~~~~~~~~~~~~~~~~~~~~~~~~~~~~~~~~~~~~~~~~~~~~~~~~~~~~~~~~~~~~~~~~~~~~<1           7<----------------------------------------------------------------------------------------------------<7           1<~~~~~~~~~~~~~~~~~~~~~~~~~~~~~~~~~~~~~~~~~~~~~~~~~~~~~~~~~~~~~~~~~~~~~~~~~~~~~~~~~~~~~~~~~~~~~~~~~~~~<1           7<----------------------------------------------------------------------------------------------------<7                    *         *         *         *         *         *         *         *         *         *         T  T  D  R  E  D  N  P  I  A  E  N  L  E  P  E  W  R  D  L  R  T  V  H  D  G  M  N  H  F  F  A  T  L       GTggtGTCacgCTCgtcGTTtggTATggcTTCattCAGctcCGGttcCCAacgATCaagGCGagtTACatgATCcccCATgttGTGaaaAAAagcGGTta  8583>gtggtgtcacgctcgtcgtttggtatggcttcattcagctccggttcccaacgatcaaggcgagttacatgatcccccatgttgtgaaaaaaagcggtta>8682        1<~~~~~~~~~~~~~~~~~~~~~~~~~~~~~~~~~~~~~~~~~~~~~~~~~~~~~~~~~~~~~~~~~~~~~~~~~~~~~~~~~~~~~~~~~~~~~~~~~~~~<1           1<~~~~~~~~~~~~~~~~~~~~~~~~~~~~~~~~~~~~~~~~~~~~~~~~~~~~~~~~~~~~~~~~~~~~~~~~~~~~~~~~~~~~~~~~~~~~~~~~~~~~<1           1<~~~~~~~~~~~~~~~~~~~~~~~~~~~~~~~~~~~~~~~~~~~~~~~~~~~~~~~~~~~~~~~~~~~~~~~~~~~~~~~~~~~~~~~~~~~~~~~~~~~~<1           7<----------------------------------------------------------------------------------------------------<7           1<~~~~~~~~~~~~~~~~~~~~~~~~~~~~~~~~~~~~~~~~~~~~~~~~~~~~~~~~~~~~~~~~~~~~~~~~~~~~~~~~~~~~~~~~~~~~~~~~~~~~<1           7<----------------------------------------------------------------------------------------------------<7                    *         *         *         *         *         *         *         *         *         *           E  K  P  G  G  I  T  T  L  L  L  N  A  A  T  N  D  S  M  T  I  A  A  S  C  L  E  R  V  T  M  G  D        gCTCcttCGGtccTCCgatCGTtgtCAGaagTAAgttGGCcgcAGTgttATCactCATggtTATggcAGCactGCAtaaTTCtctTACtgtCATgccATC  8683>gctccttcggtcctccgatcgttgtcagaagtaagttggccgcagtgttatcactcatggttatggcagcactgcataattctcttactgtcatgccatc>8782        1<~~~~~~~~~~~~~~~~~~~~~~~~~~~~~~~~~~~~~~~~~~~~~~~~~~~~~~~~~~~~~~~~~~~~~~~~~~~~~~~~~~~~~~~~~~~~~~~~~~~~<1           1<~~~~~~~~~~~~~~~~~~~~~~~~~~~~~~~~~~~~~~~~~~~~~~~~~~~~~~~~~~~~~~~~~~~~~~~~~~~~~~~~~~~~~~~~~~~~~~~~~~~~<1           1<~~~~~~~~~~~~~~~~~~~~~~~~~~~~~~~~~~~~~~~~~~~~~~~~~~~~~~~~~~~~~~~~~~~~~~~~~~~~~~~~~~~~~~~~~~~~~~~~~~~~<1           7<----------------------------------------------------------------------------------------------------<7           1<~~~~~~~~~~~~~~~~~~~~~~~~~~~~~~~~~~~~~~~~~~~~~~~~~~~~~~~~~~~~~~~~~~~~~~~~~~~~~~~~~~~~~~~~~~~~~~~~~~~~<1           7<----------------------------------------------------------------------------------------------------<7                    *         *         *         *         *         *         *         *         *         *          T  L  H  K  E  T  V  P  S  Y  E  V  L  D  N  Q  S  Y  H  I  R  R  G  L  Q  E  Q  G  A  D  I  R  S         cgtAAGatgCTTttcTGTgacTGGtgaGTActcAACcaaGTCattCTGagaATAgtgTATgcgGCGaccGAGttgCTCttgCCCggcGTCaatACGggaT  8783>cgtaagatgcttttctgtgactggtgagtactcaaccaagtcattctgagaatagtgtatgcggcgaccgagttgctcttgcccggcgtcaatacgggat>8882        1<~~~~~~~~~~~~~~~~~~~~~~~~~~~~~~~~~~~~~~~~~~~~~~~~~~~~~~~~~~~~~~~~~~~~~~~~~~~~~~~~~~~~~~~~~~~~~~~~~~~~<1           1<~~~~~~~~~~~~~~~~~~~~~~~~~~~~~~~~~~~~~~~~~~~~~~~~~~~~~~~~~~~~~~~~~~~~~~~~~~~~~~~~~~~~~~~~~~~~~~~~~~~~<1           1<~~~~~~~~~~~~~~~~~~~~~~~~~~~~~~~~~~~~~~~~~~~~~~~~~~~~~~~~~~~~~~~~~~~~~~~~~~~~~~~~~~~~~~~~~~~~~~~~~~~~<1           7<----------------------------------------------------------------------------------------------------<7           1<~~~~~~~~~~~~~~~~~~~~~~~~~~~~~~~~~~~~~~~~~~~~~~~~~~~~~~~~~~~~~~~~~~~~~~~~~~~~~~~~~~~~~~~~~~~~~~~~~~~~<1           7<----------------------------------------------------------------------------------------------------<7                    *         *         *         *         *         *         *         *         *         *         L  V  A  G  C  L  L  V  K  F  T  S  M  M  P  F  R  E  E  P  R  F  S  E  L  I  K  G  S  N  L  D  L  E       AAtacCGCgccACAtagCAGaacTTTaaaAGTgctCATcatTGGaaaACGttcTTCgggGCGaaaACTctcAAGgatCTTaccGCTgttGAGatcCAGtt  8883>aataccgcgccacatagcagaactttaaaagtgctcatcattggaaaacgttcttcggggcgaaaactctcaaggatcttaccgctgttgagatccagtt>8982        1<~~~~~~~~~~~~~~~~~~~~~~~~~~~~~~~~~~~~~~~~~~~~~~~~~~~~~~~~~~~~~~~~~~~~~~~~~~~~~~~~~~~~~~~~~~~~~~~~~~~~<1           1<~~~~~~~~~~~~~~~~~~~~~~~~~~~~~~~~~~~~~~~~~~~~~~~~~~~~~~~~~~~~~~~~~~~~~~~~~~~~~~~~~~~~~~~~~~~~~~~~~~~~<1           1<~~~~~~~~~~~~~~~~~~~~~~~~~~~~~~~~~~~~~~~~~~~~~~~~~~~~~~~~~~~~~~~~~~~~~~~~~~~~~~~~~~~~~~~~~~~~~~~~~~~~<1           7<----------------------------------------------------------------------------------------------------<7           1<~~~~~~~~~~~~~~~~~~~~~~~~~~~~~~~~~~~~~~~~~~~~~~~~~~~~~~~~~~~~~~~~~~~~~~~~~~~~~~~~~~~~~~~~~~~~~~~~~~~~<1           7<----------------------------------------------------------------------------------------------------<7                    *         *         *         *         *         *         *         *         *         *           I  Y  G  V  R  A  G  L  Q  D  E  A  D  K  V  K  V  L  T  E  P  H  A  F  V  P  L  C  F  A  A  F  F        cGATgtaACCcacTCGtgcACCcaaCTGatcTTCagcATCtttTACtttCACcagCGTttcTGGgtgAGCaaaAACaggAAGgcaAAAtgcCGCaaaAAA  8983>cgatgtaacccactcgtgcacccaactgatcttcagcatcttttactttcaccagcgtttctgggtgagcaaaaacaggaaggcaaaatgccgcaaaaaa>9082        1<~~~~~~~~~~~~~~~~~~~~~~~~~~~~~~~~~~~~~~~~~~~~~~~~~~~~~~~~~~~~~~~~~~~~~~~~~~~~~~~~~~~~~~~~~~~~~~~~~~~~<1           1<~~~~~~~~~~~~~~~~~~~~~~~~~~~~~~~~~~~~~~~~~~~~~~~~~~~~~~~~~~~~~~~~~~~~~~~~~~~~~~~~~~~~~~~~~~~~~~~~~~~~<1           1<~~~~~~~~~~~~~~~~~~~~~~~~~~~~~~~~~~~~~~~~~~~~~~~~~~~~~~~~~~~~~~~~~~~~~~~~~~~~~~~~~~~~~~~~~~~~~~~~~~~~<1           7<----------------------------------------------------------------------------------------------------<7           1<~~~~~~~~~~~~~~~~~~~~~~~~~~~~~~~~~~~~~~~~~~~~~~~~~~~~~~~~~~~~~~~~~~~~~~~~~~~~~~~~~~~~~~~~~~~~~~~~~~~~<1           7<----------------------------------------------------------------------------------------------------<7                    *         *         *         *         *         *         *         *         *         *          P  I  L  A  V  R  F  H  Q  I  S  M                                                                        gggAATaagGGCgacACGgaaATGttgAATactCAT                                                                  9083>gggaataagggcgacacggaaatgttgaatactcatactcttcctttttcaatattattgaagcatttatcagggttattgtctcatgagcggatacata>9182        1<~~~~~~~~~~~~~~~~~~~~~~~~~~~~~~~~~~~~~~~~~~~~~~~~~~~~~~~~~~~~~~~~~~~~~~~~~~~~~~~~~~~~~~~~~~~~~~~~~~~~<1           1<~~~~~~~~~~~~~~~~~~~~~~~~~~~~~~~~~~~~~~~~~~~~~~~~~~~~~~~~~~~~~~~~~~~~~~~~~~~~~~~~~~~~~~~~~~~~~~~~~~~~<1           1<~~~~~~~~~~~~~~~~~~~~~~~~~~~~~~~~~~~~~~~~~~~~~~~~~~~~~~~~~~~~~~~~~~~~~~~~~~~~~~~~~~~~~~~~~~~~~~~~~~~~<1           6<-------------------------------------------------------------------------------------------GGAT-----<3           1<~~~~~~~~~~~~~~~~~~~~~~~~~~~~~~~~~~~~~~~~~~~~~~~~~~~~~~~~~~~~~~~~~~~~~~~~~~~~~~~~~~~~~~~~~~~~~~~~~~~~<1           7<----------------------------------------------------------------------------------------------------<7                    *         *         *         *         *         *         *         *         *         *    9183>tttgaatgtatttagaaaaataaacaaataggggttccgcgcacatttccccgaaaagtgccacctgacgtcttattatcatgacattaacctataaaaa>9282        1<~~~~~~~~~~~~~~~~~~~~~~~~~~~~~~~~~~~~~~~~~~~~~~~~~~~~~~~~~~~~~~~~~~~~~~~~~~~~~~~~~~~~~~~~~~~~~~~~~~~~<1           1<~~~~~~~~~~~~~~~~~~~~~~~~~~~~~~~~~~~~~~~~~~~~~~~~~~~~~~~~~~~~~~~~~~~~~~~~~~~~~~~~~~~~~~~~~~~~~~~~~~~~<1           1<~~~~~~~~~~~~~~~~~~~~~~~~~~~~~~~~~~~~~~~~~~~~~~~~~~~~~~~~~~~~~~~~~~~~~~~~~~~~~~~~~~~~~~~~~~~~~~~~~~~~<1           3<----------------------------------------------------------------------------------------------------<3           1<~~~~~~~~~~~~~~~~~~~~~~~~~~~~~~~~~~~~~~~~~~~~~~~~~~~~~~~~~~~~~~~~~~~~~~~~~~~~~~~~~~~~~~~~~~~~~~~~~~~~<1           6<---------------------------------------------------------GTG----------------------------------------<4                    *         *  9283>taggcgtatcacgaggccctttcgtc>9308        1<~~~~~~~~~~~~~~~~~~~~~~~~~~<1           1<~~~~~~~~~~~~~~~~~~~~~~~~~~<1           1<~~~~~~~~~~~~~~~~~~~~~~~~~~<1           2<------------------------TC<1           1<~~~~~~~~~~~~~~~~~~~~~~~~~~<1           3<-------------------TTT~~~~<1      
