# Supplementary material for: AssemblyTron: flexible automation of DNA assembly with Opentrons OT-2 lab robots
Source: Synth Biol (Oxf). 2022 Dec 22;8(1):ysac032. doi: 10.1093/synbio/ysac032 (PMC9832943; doi:10.1093/synbio/ysac032)
Supplement: ysac032_Supp [file ysac032_supp.zip › suppl_data/File6_rxn2.rtf]

Mon Sep 12, 2022 11:11 EDT-ARF7-pdar-pmas00002.gb from 1 to 10043to_F10_IVA_rxn2_C6+206_TCYC1_Seq_R.ab1--  Matches:1130; Mismatches:0; Gaps:3632; Unattempted:5427_E10_IVA_rxn2_C5+206_TCYC1_Seq_R.ab1--  Matches:1300; Mismatches:2; Gaps:3487; Unattempted:5256_D10_IVA_rxn2_C4+206_TCYC1_Seq_R.ab1--  Matches:996; Mismatches:1; Gaps:3777; Unattempted:5562_H03_IVA_rxn2_C3+206_TCYC1_Seq_R.ab1--  Matches:579; Mismatches:2; Gaps:3624; Unattempted:5983_F03_IVA_rxn2_C1+206_TCYC1_Seq_R.ab1--  Matches:1190; Mismatches:0; Gaps:3486; Unattempted:5367_G03_IVA_rxn2_C2+206_TCYC1_Seq_R.ab1--  Matches:579; Mismatches:2; Gaps:3624; Unattempted:5983                *         *         *         *         *         *         *         *         *         *     1>tcgcgcgtttcggtgatgacggtgaaaacctctgacacatgcagctcccggagacggtcacagcttgtctgtaagcggatgccgggagcagacaagcccg>100      1434<T---------------------------------------------------------------------------------------------------<1434     1450<T---------------------------------------------------------------------------------------------------<1450     1479<~~~~~~~~~~~~~~~~~~~~~~~~~~~~~~~~~~~~~~~~~~~~~~~~~~~~~~~~~~~~~~~~~~~~~~~~~~~~~~~~~~~~~~~~~~~~~~~~~~~~<1479     1395<~~~~~~~~~~~~~~~~~~~~~~~~~~~~~~~~~~~~~~~~~~~~~~~~~~~~~~~~~~~~~~~~~~~~~~~~~~~~~~~~~~~~~~~~~~~~~~~~~~~~<1395     1388<~~~~~~~~~~~~~~~~~~~~~~~~~~~~~~~~~~~~~~~~~~~~~~~~~~~~~~~~~~~~~~~~~~~~~~~~~~~~~~~~~~~~~~~~~~~~~~~~~~~~<1388     1395<~~~~~~~~~~~~~~~~~~~~~~~~~~~~~~~~~~~~~~~~~~~~~~~~~~~~~~~~~~~~~~~~~~~~~~~~~~~~~~~~~~~~~~~~~~~~~~~~~~~~<1395                   *         *         *         *         *         *         *         *         *         *   101>tcagggcgcgtcagcgggtgttggcgggtgtcggggctggcttaactatgcggcatcagagcagattgtactgagagtgcaccataattcgtttaaaccg>200      1434<----------------------------------------------------------------------------------------------------<1434     1450<----------------------------------------------------------------------------------------------------<1450     1479<~~~~~~~~~~~~~~~~~~~~~~~~~~~~~~~~~~~~~~~~~~~~~~~~~~~~~~~~~~~~~~~~~~~~~~~~~~~~~~~~~~~~~~~~~~~~~~~~~~~~<1479     1395<~~~~~~~~~~~~~~~~~~~~~~~~~~~~~~~~~~~~~~~~~~~~~~~~~~~~~~~~~~~~~~~~~~~~~~~~~~~~~~~~~~~~~~~~~~~~~~~~~~~~<1395     1388<~~~~~~~~~~~~~~~~~~~~~~~~~~~~~~~~~~~~~~~~~~~~~~~~~~~~~~~~~~~~~~~~~~~~~~~~~~~~~~~~~~~~~~~~~~~~~~~~~~~~<1388     1395<~~~~~~~~~~~~~~~~~~~~~~~~~~~~~~~~~~~~~~~~~~~~~~~~~~~~~~~~~~~~~~~~~~~~~~~~~~~~~~~~~~~~~~~~~~~~~~~~~~~~<1395                   *         *         *         *         *         *         *         *         *         *   201>ttttaagagcttggtgagcgctaggagtcactgccaggtatcgtttgaacacggcattagtcagggaagtcataacacagtcctttcccgcaattttctt>300      1434<----------------------------------------------------------------------------------------------------<1434     1450<----------------------------------------------------------------------------------------------------<1450     1479<~~~~~~~~~~~~~~~~~~~~~~~~~~~~~~~~~~~~~~~~~~~~~~~~~~~~~~~~~~~~~~~~~~~~~~~~~~~~~~~~~~~~~~~~~~~~~~~~~~~~<1479     1395<~~~~~~~~~~~~~~~~~~~~~~~~~~~~~~~~~~~~~~~~~~~~~~~~~~~~~~~~~~~~~~~~~~~~~~~~~~~~~~~~~~~~~~~~~~~~~~~~~~~~<1395     1388<~~~~~~~~~~~~~~~~~~~~~~~~~~~~~~~~~~~~~~~~~~~~~~~~~~~~~~~~~~~~~~~~~~~~~~~~~~~~~~~~~~~~~~~~~~~~~~~~~~~~<1388     1395<~~~~~~~~~~~~~~~~~~~~~~~~~~~~~~~~~~~~~~~~~~~~~~~~~~~~~~~~~~~~~~~~~~~~~~~~~~~~~~~~~~~~~~~~~~~~~~~~~~~~<1395                   *         *         *         *         *         *         *         *         *         *   301>tttctattactcttggcctcctctagtacactctatatttttttatgcctcggtaatgattttcatttttttttttccacctagcggatgactctttttt>400      1434<----------------------------------------------------------------------------------------------------<1434     1450<----------------------------------------------------------------------------------------------------<1450     1479<~~~~~~~~~~~~~~~~~~~~~~~~~~~~~~~~~~~~~~~~~~~~~~~~~~~~~~~~~~~~~~~~~~~~~~~~~~~~~~~~~~~~~~~~~~~~~~~~~~~~<1479     1395<~~~~~~~~~~~~~~~~~~~~~~~~~~~~~~~~~~~~~~~~~~~~~~~~~~~~~~~~~~~~~~~~~~~~~~~~~~~~~~~~~~~~~~~~~~~~~~~~~~~~<1395     1388<~~~~~~~~~~~~~~~~~~~~~~~~~~~~~~~~~~~~~~~~~~~~~~~~~~~~~~~~~~~~~~~~~~~~~~~~~~~~~~~~~~~~~~~~~~~~~~~~~~~~<1388     1395<~~~~~~~~~~~~~~~~~~~~~~~~~~~~~~~~~~~~~~~~~~~~~~~~~~~~~~~~~~~~~~~~~~~~~~~~~~~~~~~~~~~~~~~~~~~~~~~~~~~~<1395                   *         *         *         *         *         *         *         *         *         *   401>tttcttagcgattggcattatcacataatgaattatacattatataaagtaatgtgatttcttcgaagaatatactaaaaaatgagcaggcaagataaac>500      1434<----------------------------------------------------------------------------------------------------<1434     1450<----------------------------------------------------------------------------------------------------<1450     1479<~~~~~~~~~~~~~~~~~~~~~~~~~~~~~~~~~~~~~~~~~~~~~~~~~~~~~~~~~~~~~~~~~~~~~~~~~~~~~~~~~~~~~~~~~~~~~~~~~~~~<1479     1395<~~~~~~~~~~~~~~~~~~~~~~~~~~~~~~~~~~~~~~~~~~~~~~~~~~~~~~~~~~~~~~~~~~~~~~~~~~~~~~~~~~~~~~~~~~~~~~~~~~~~<1395     1388<~~~~~~~~~~~~~~~~~~~~~~~~~~~~~~~~~~~~~~~~~~~~~~~~~~~~~~~~~~~~~~~~~~~~~~~~~~~~~~~~~~~~~~~~~~~~~~~~~~~~<1388     1395<~~~~~~~~~~~~~~~~~~~~~~~~~~~~~~~~~~~~~~~~~~~~~~~~~~~~~~~~~~~~~~~~~~~~~~~~~~~~~~~~~~~~~~~~~~~~~~~~~~~~<1395                   *         *         *         *         *         *         *         *         *         *   501>gaaggcaaagatgacagagcagaaagccctagtaaagcgtattacaaatgaaaccaagattcagattgcgatctctttaaagggtggtcccctagcgata>600      1434<----------------------------------------------------------------------------------------------------<1434     1450<----------------------------------------------------------------------------------------------------<1450     1479<~~~~~~~~~~~~~~~~~~~~~~~~~~~~~~~~~~~~~~~~~~~~~~~~~~~~~~~~~~~~~~~~~~~~~~~~~~~~~~~~~~~~~~~~~~~~~~~~~~~~<1479     1395<~~~~~~~~~~~~~~~~~~~~~~~~~~~~~~~~~~~~~~~~~~~~~~~~~~~~~~~~~~~~~~~~~~~~~~~~~~~~~~~~~~~~~~~~~~~~~~~~~~~~<1395     1388<~~~~~~~~~~~~~~~~~~~~~~~~~~~~~~~~~~~~~~~~~~~~~~~~~~~~~~~~~~~~~~~~~~~~~~~~~~~~~~~~~~~~~~~~~~~~~~~~~~~~<1388     1395<~~~~~~~~~~~~~~~~~~~~~~~~~~~~~~~~~~~~~~~~~~~~~~~~~~~~~~~~~~~~~~~~~~~~~~~~~~~~~~~~~~~~~~~~~~~~~~~~~~~~<1395                   *         *         *         *         *         *         *         *         *         *   601>gagcactcgatcttcccagaaaaagaggcagaagcagtagcagaacaggccacacaatcgcaagtgattaacgtccacacaggtatagggtttctggacc>700      1434<----------------------------------------------------------------------------------------------------<1434     1450<----------------------------------------------------------------------------------------------------<1450     1479<~~~~~~~~~~~~~~~~~~~~~~~~~~~~~~~~~~~~~~~~~~~~~~~~~~~~~~~~~~~~~~~~~~~~~~~~~~~~~~~~~~~~~~~~~~~~~~~~~~~~<1479     1395<~~~~~~~~~~~~~~~~~~~~~~~~~~~~~~~~~~~~~~~~~~~~~~~~~~~~~~~~~~~~~~~~~~~~~~~~~~~~~~~~~~~~~~~~~~~~~~~~~~~~<1395     1388<~~~~~~~~~~~~~~~~~~~~~~~~~~~~~~~~~~~~~~~~~~~~~~~~~~~~~~~~~~~~~~~~~~~~~~~~~~~~~~~~~~~~~~~~~~~~~~~~~~~~<1388     1395<~~~~~~~~~~~~~~~~~~~~~~~~~~~~~~~~~~~~~~~~~~~~~~~~~~~~~~~~~~~~~~~~~~~~~~~~~~~~~~~~~~~~~~~~~~~~~~~~~~~~<1395                   *         *         *         *         *         *         *         *         *         *   701>atatgatacatgctctggccaagcattccggctggtcgctaatcgttgagtgcattggtgacttacacatagacgaccatcacaccactgaagactgcgg>800      1434<----------------------------------------------------------------------------------------------------<1434     1450<----------------------------------------------------------------------------------------------------<1450     1479<~~~~~~~~~~~~~~~~~~~~~~~~~~~~~~~~~~~~~~~~~~~~~~~~~~~~~~~~~~~~~~~~~~~~~~~~~~~~~~~~~~~~~~~~~~~~~~~~~~~~<1479     1395<~~~~~~~~~~~~~~~~~~~~~~~~~~~~~~~~~~~~~~~~~~~~~~~~~~~~~~~~~~~~~~~~~~~~~~~~~~~~~~~~~~~~~~~~~~~~~~~~~~~~<1395     1388<~~~~~~~~~~~~~~~~~~~~~~~~~~~~~~~~~~~~~~~~~~~~~~~~~~~~~~~~~~~~~~~~~~~~~~~~~~~~~~~~~~~~~~~~~~~~~~~~~~~~<1388     1395<~~~~~~~~~~~~~~~~~~~~~~~~~~~~~~~~~~~~~~~~~~~~~~~~~~~~~~~~~~~~~~~~~~~~~~~~~~~~~~~~~~~~~~~~~~~~~~~~~~~~<1395                   *         *         *         *         *         *         *         *         *         *   801>gattgctctcggtcaagcttttaaagaggccctaggggccgtgcgtggagtaaaaaggtttggatcaggatttgcgcctttggatgaggcactttccaga>900      1434<----------------------------------------------------------------------------------------------------<1434     1450<----------------------------------------------------------------------------------------------------<1450     1479<~~~~~~~~~~~~~~~~~~~~~~~~~~~~~~~~~~~~~~~~~~~~~~~~~~~~~~~~~~~~~~~~~~~~~~~~~~~~~~~~~~~~~~~~~~~~~~~~~~~~<1479     1395<~~~~~~~~~~~~~~~~~~~~~~~~~~~~~~~~~~~~~~~~~~~~~~~~~~~~~~~~~~~~~~~~~~~~~~~~~~~~~~~~~~~~~~~~~~~~~~~~~~~~<1395     1388<~~~~~~~~~~~~~~~~~~~~~~~~~~~~~~~~~~~~~~~~~~~~~~~~~~~~~~~~~~~~~~~~~~~~~~~~~~~~~~~~~~~~~~~~~~~~~~~~~~~~<1388     1395<~~~~~~~~~~~~~~~~~~~~~~~~~~~~~~~~~~~~~~~~~~~~~~~~~~~~~~~~~~~~~~~~~~~~~~~~~~~~~~~~~~~~~~~~~~~~~~~~~~~~<1395                   *         *         *         *         *         *         *         *         *         *   901>gcggtggtagatctttcgaacaggccgtacgcagttgtcgaacttggtttgcaaagggagaaagtaggagatctctcttgcgagatgatcccgcattttc>1000     1434<----------------------------------------------------------------------------------------------------<1434     1450<----------------------------------------------------------------------------------------------------<1450     1479<~~~~~~~~~~~~~~~~~~~~~~~~~~~~~~~~~~~~~~~~~~~~~~~~~~~~~~~~~~~~~~~~~~~~~~~~~~~~~~~~~~~~~~~~~~~~~~~~~~~~<1479     1395<~~~~~~~~~~~~~~~~~~~~~~~~~~~~~~~~~~~~~~~~~~~~~~~~~~~~~~~~~~~~~~~~~~~~~~~~~~~~~~~~~~~~~~~~~~~~~~~~~~~~<1395     1388<~~~~~~~~~~~~~~~~~~~~~~~~~~~~~~~~~~~~~~~~~~~~~~~~~~~~~~~~~~~~~~~~~~~~~~~~~~~~~~~~~~~~~~~~~~~~~~~~~~~~<1388     1395<~~~~~~~~~~~~~~~~~~~~~~~~~~~~~~~~~~~~~~~~~~~~~~~~~~~~~~~~~~~~~~~~~~~~~~~~~~~~~~~~~~~~~~~~~~~~~~~~~~~~<1395                   *         *         *         *         *         *         *         *         *         *  1001>ttgaaagctttgcagaggctagcagaattaccctccacgttgattgtctgcgaggcaagaatgatcatcaccgtagtgagagtgcgttcaaggctcttgc>1100     1434<----------------------------------------------------------------------------------------------------<1434     1450<----------------------------------------------------------------------------------------------------<1450     1479<~~~~~~~~~~~~~~~~~~~~~~~~~~~~~~~~~~~~~~~~~~~~~~~~~~~~~~~~~~~~~~~~~~~~~~~~~~~~~~~~~~~~~~~~~~~~~~~~~~~~<1479     1395<~~~~~~~~~~~~~~~~~~~~~~~~~~~~~~~~~~~~~~~~~~~~~~~~~~~~~~~~~~~~~~~~~~~~~~~~~~~~~~~~~~~~~~~~~~~~~~~~~~~~<1395     1388<~~~~~~~~~~~~~~~~~~~~~~~~~~~~~~~~~~~~~~~~~~~~~~~~~~~~~~~~~~~~~~~~~~~~~~~~~~~~~~~~~~~~~~~~~~~~~~~~~~~~<1388     1395<~~~~~~~~~~~~~~~~~~~~~~~~~~~~~~~~~~~~~~~~~~~~~~~~~~~~~~~~~~~~~~~~~~~~~~~~~~~~~~~~~~~~~~~~~~~~~~~~~~~~<1395                   *         *         *         *         *         *         *         *         *         *  1101>ggttgccataagagaagccacctcgcccaatggtaccaacgatgttccctccaccaaaggtgttcttatgtaggcgaatttcttatgatttatgattttt>1200     1434<----------------------------------------------------------------------------------------------------<1434     1450<----------------------------------------------------------------------------------------------------<1450     1479<~~~~~~~~~~~~~~~~~~~~~~~~~~~~~~~~~~~~~~~~~~~~~~~~~~~~~~~~~~~~~~~~~~~~~~~~~~~~~~~~~~~~~~~~~~~~~~~~~~~~<1479     1395<~~~~~~~~~~~~~~~~~~~~~~~~~~~~~~~~~~~~~~~~~~~~~~~~~~~~~~~~~~~~~~~~~~~~~~~~~~~~~~~~~~~~~~~~~~~~~~~~~~~~<1395     1388<~~~~~~~~~~~~~~~~~~~~~~~~~~~~~~~~~~~~~~~~~~~~~~~~~~~~~~~~~~~~~~~~~~~~~~~~~~~~~~~~~~~~~~~~~~~~~~~~~~~~<1388     1395<~~~~~~~~~~~~~~~~~~~~~~~~~~~~~~~~~~~~~~~~~~~~~~~~~~~~~~~~~~~~~~~~~~~~~~~~~~~~~~~~~~~~~~~~~~~~~~~~~~~~<1395                   *         *         *         *         *         *         *         *         *         *  1201>attattaaataagttataaaaaaaataagtgtatacaaattttaaagtgactcttaggttttaaaacgaaaattcttattcttgagtaactctttcctgt>1300     1434<----------------------------------------------------------------------------------------------------<1434     1450<----------------------------------------------------------------------------------------------------<1450     1479<~~~~~~~~~~~~~~~~~~~~~~~~~~~~~~~~~~~~~~~~~~~~~~~~~~~~~~~~~~~~~~~~~~~~~~~~~~~~~~~~~~~~~~~~~~~~~~~~~~~~<1479     1395<~~~~~~~~~~~~~~~~~~~~~~~~~~~~~~~~~~~~~~~~~~~~~~~~~~~~~~~~~~~~~~~~~~~~~~~~~~~~~~~~~~~~~~~~~~~~~~~~~~~~<1395     1388<~~~~~~~~~~~~~~~~~~~~~~~~~~~~~~~~~~~~~~~~~~~~~~~~~~~~~~~~~~~~~~~~~~~~~~~~~~~~~~~~~~~~~~~~~~~~~~~~~~~~<1388     1395<~~~~~~~~~~~~~~~~~~~~~~~~~~~~~~~~~~~~~~~~~~~~~~~~~~~~~~~~~~~~~~~~~~~~~~~~~~~~~~~~~~~~~~~~~~~~~~~~~~~~<1395                   *         *         *         *         *         *         *         *         *         *  1301>aggtcaggttgctttctcaggtatagcatgaggtcgctcttattgaccacacctcaagaaatgatggtaaatgaaataggaaatcaaggagcatgaaggc>1400     1434<----------------------------------------------------------------------------------------------------<1434     1450<----------------------------------------------------------------------------------------------------<1450     1479<~~~~~~~~~~~~~~~~~~~~~~~~~~~~~~~~~~~~~~~~~~~~~~~~~~~~~~~~~~~~~~~~~~~~~~~~~~~~~~~~~~~~~~~~~~~~~~~~~~~~<1479     1395<~~~~~~~~~~~~~~~~~~~~~~~~~~~~~~~~~~~~~~~~~~~~~~~~~~~~~~~~~~~~~~~~~~~~~~~~~~~~~~~~~~~~~~~~~~~~~~~~~~~~<1395     1388<~~~~~~~~~~~~~~~~~~~~~~~~~~~~~~~~~~~~~~~~~~~~~~~~~~~~~~~~~~~~~~~~~~~~~~~~~~~~~~~~~~~~~~~~~~~~~~~~~~~~<1388     1395<~~~~~~~~~~~~~~~~~~~~~~~~~~~~~~~~~~~~~~~~~~~~~~~~~~~~~~~~~~~~~~~~~~~~~~~~~~~~~~~~~~~~~~~~~~~~~~~~~~~~<1395                   *         *         *         *         *         *         *         *         *         *  1401>aaaagacaaatataagggtcgaacgaaaaataaagtgaaaagtgttgatatgatgtatttggctttgcggcgccgaaaaaacgagtttacgcaattgcac>1500     1434<----------------------------------------------------------------------------------------------------<1434     1450<----------------------------------------------------------------------------------------------------<1450     1479<~~~~~~~~~~~~~~~~~~~~~~~~~~~~~~~~~~~~~~~~~~~~~~~~~~~~~~~~~~~~~~~~~~~~~~~~~~~~~~~~~~~~~~~~~~~~~~~~~~~~<1479     1395<~~~~~~~~~~~~~~~~~~~~~~~~~~~~~~~~~~~~~~~~~~~~~~~~~~~~~~~~~~~~~~~~~~~~~~~~~~~~~~~~~~~~~~~~~~~~~~~~~~~~<1395     1388<~~~~~~~~~~~~~~~~~~~~~~~~~~~~~~~~~~~~~~~~~~~~~~~~~~~~~~~~~~~~~~~~~~~~~~~~~~~~~~~~~~~~~~~~~~~~~~~~~~~~<1388     1395<~~~~~~~~~~~~~~~~~~~~~~~~~~~~~~~~~~~~~~~~~~~~~~~~~~~~~~~~~~~~~~~~~~~~~~~~~~~~~~~~~~~~~~~~~~~~~~~~~~~~<1395                   *         *         *         *         *         *         *         *         *         *  1501>aatcatgctgactctgtggcggacccgcgctcttgccggcccggcgataacgctgggcgtgaggctgtgcccggcggagttttttgcgcctgcattttcc>1600     1434<----------------------------------------------------------------------------------------------------<1434     1450<----------------------------------------------------------------------------------------------------<1450     1479<~~~~~~~~~~~~~~~~~~~~~~~~~~~~~~~~~~~~~~~~~~~~~~~~~~~~~~~~~~~~~~~~~~~~~~~~~~~~~~~~~~~~~~~~~~~~~~~~~~~~<1479     1395<~~~~~~~~~~~~~~~~~~~~~~~~~~~~~~~~~~~~~~~~~~~~~~~~~~~~~~~~~~~~~~~~~~~~~~~~~~~~~~~~~~~~~~~~~~~~~~~~~~~~<1395     1388<~~~~~~~~~~~~~~~~~~~~~~~~~~~~~~~~~~~~~~~~~~~~~~~~~~~~~~~~~~~~~~~~~~~~~~~~~~~~~~~~~~~~~~~~~~~~~~~~~~~~<1388     1395<~~~~~~~~~~~~~~~~~~~~~~~~~~~~~~~~~~~~~~~~~~~~~~~~~~~~~~~~~~~~~~~~~~~~~~~~~~~~~~~~~~~~~~~~~~~~~~~~~~~~<1395                   *         *         *         *         *         *         *         *         *         *  1601>aaggtttaccctgcgctaaggggcgagattggagaagcaataagaatgccggttggggttgcgatgatgacgaccacgacaactggtgtcattatttaag>1700     1434<----------------------------------------------------------------------------------------------------<1434     1450<----------------------------------------------------------------------------------------------------<1450     1479<~~~~~~~~~~~~~~~~~~~~~~~~~~~~~~~~~~~~~~~~~~~~~~~~~~~~~~~~~~~~~~~~~~~~~~~~~~~~~~~~~~~~~~~~~~~~~~~~~~~~<1479     1395<~~~~~~~~~~~~~~~~~~~~~~~~~~~~~~~~~~~~~~~~~~~~~~~~~~~~~~~~~~~~~~~~~~~~~~~~~~~~~~~~~~~~~~~~~~~~~~~~~~~~<1395     1388<~~~~~~~~~~~~~~~~~~~~~~~~~~~~~~~~~~~~~~~~~~~~~~~~~~~~~~~~~~~~~~~~~~~~~~~~~~~~~~~~~~~~~~~~~~~~~~~~~~~~<1388     1395<~~~~~~~~~~~~~~~~~~~~~~~~~~~~~~~~~~~~~~~~~~~~~~~~~~~~~~~~~~~~~~~~~~~~~~~~~~~~~~~~~~~~~~~~~~~~~~~~~~~~<1395                   *         *         *         *         *         *         *         *         *         *  1701>ttgccgaaagaacctgagtgcatttgcaacatgagtatactagaagaatgagccaagacttgcgagacgcgagtttgccggtggtgcgaacaatagagcg>1800     1434<----------------------------------------------------------------------------------------------------<1434     1450<----------------------------------------------------------------------------------------------------<1450     1479<~~~~~~~~~~~~~~~~~~~~~~~~~~~~~~~~~~~~~~~~~~~~~~~~~~~~~~~~~~~~~~~~~~~~~~~~~~~~~~~~~~~~~~~~~~~~~~~~~~~~<1479     1395<~~~~~~~~~~~~~~~~~~~~~~~~~~~~~~~~~~~~~~~~~~~~~~~~~~~~~~~~~~~~~~~~~~~~~~~~~~~~~~~~~~~~~~~~~~~~~~~~~~~~<1395     1388<~~~~~~~~~~~~~~~~~~~~~~~~~~~~~~~~~~~~~~~~~~~~~~~~~~~~~~~~~~~~~~~~~~~~~~~~~~~~~~~~~~~~~~~~~~~~~~~~~~~~<1388     1395<~~~~~~~~~~~~~~~~~~~~~~~~~~~~~~~~~~~~~~~~~~~~~~~~~~~~~~~~~~~~~~~~~~~~~~~~~~~~~~~~~~~~~~~~~~~~~~~~~~~~<1395                   *         *         *         *         *         *         *         *         *         *  1801>accatgaccttgaaggtgagacgcgcataaccgctagagtactttgaagaggaaacagcaatagggttgctaccagtataaatagacaggtacatacaac>1900     1434<----------------------------------------------------------------------------------------------------<1434     1450<----------------------------------------------------------------------------------------------------<1450     1479<~~~~~~~~~~~~~~~~~~~~~~~~~~~~~~~~~~~~~~~~~~~~~~~~~~~~~~~~~~~~~~~~~~~~~~~~~~~~~~~~~~~~~~~~~~~~~~~~~~~~<1479     1395<~~~~~~~~~~~~~~~~~~~~~~~~~~~~~~~~~~~~~~~~~~~~~~~~~~~~~~~~~~~~~~~~~~~~~~~~~~~~~~~~~~~~~~~~~~~~~~~~~~~~<1395     1388<~~~~~~~~~~~~~~~~~~~~~~~~~~~~~~~~~~~~~~~~~~~~~~~~~~~~~~~~~~~~~~~~~~~~~~~~~~~~~~~~~~~~~~~~~~~~~~~~~~~~<1388     1395<~~~~~~~~~~~~~~~~~~~~~~~~~~~~~~~~~~~~~~~~~~~~~~~~~~~~~~~~~~~~~~~~~~~~~~~~~~~~~~~~~~~~~~~~~~~~~~~~~~~~<1395                   *         *         *         *         *         *         *         *         *         *  1901>actggaaatggttgtctgtttgagtacgctttcaattcatttgggtgtgcactttattatgttacaatatggaagggaactttacacttctcctatgcac>2000     1434<----------------------------------------------------------------------------------------------------<1434     1450<----------------------------------------------------------------------------------------------------<1450     1479<~~~~~~~~~~~~~~~~~~~~~~~~~~~~~~~~~~~~~~~~~~~~~~~~~~~~~~~~~~~~~~~~~~~~~~~~~~~~~~~~~~~~~~~~~~~~~~~~~~~~<1479     1395<~~~~~~~~~~~~~~~~~~~~~~~~~~~~~~~~~~~~~~~~~~~~~~~~~~~~~~~~~~~~~~~~~~~~~~~~~~~~~~~~~~~~~~~~~~~~~~~~~~~~<1395     1388<~~~~~~~~~~~~~~~~~~~~~~~~~~~~~~~~~~~~~~~~~~~~~~~~~~~~~~~~~~~~~~~~~~~~~~~~~~~~~~~~~~~~~~~~~~~~~~~~~~~~<1388     1395<~~~~~~~~~~~~~~~~~~~~~~~~~~~~~~~~~~~~~~~~~~~~~~~~~~~~~~~~~~~~~~~~~~~~~~~~~~~~~~~~~~~~~~~~~~~~~~~~~~~~<1395                   *         *         *         *         *         *         *         *         *         *  2001>atatattaattaaagtccaatgctagtagagaaggggggtaacacccctccgcgctcttttccgatttttttctaaaccgtggaatatttcggatatcct>2100     1434<----------------------------------------------------------------------------------------------------<1434     1450<----------------------------------------------------------------------------------------------------<1450     1479<~~~~~~~~~~~~~~~~~~~~~~~~~~~~~~~~~~~~~~~~~~~~~~~~~~~~~~~~~~~~~~~~~~~~~~~~~~~~~~~~~~~~~~~~~~~~~~~~~~~~<1479     1395<~~~~~~~~~~~~~~~~~~~~~~~~~~~~~~~~~~~~~~~~~~~~~~~~~~~~~~~~~~~~~~~~~~~~~~~~~~~~~~~~~~~~~~~~~~~~~~~~~~~~<1395     1388<~~~~~~~~~~~~~~~~~~~~~~~~~~~~~~~~~~~~~~~~~~~~~~~~~~~~~~~~~~~~~~~~~~~~~~~~~~~~~~~~~~~~~~~~~~~~~~~~~~~~<1388     1395<~~~~~~~~~~~~~~~~~~~~~~~~~~~~~~~~~~~~~~~~~~~~~~~~~~~~~~~~~~~~~~~~~~~~~~~~~~~~~~~~~~~~~~~~~~~~~~~~~~~~<1395                   *         *         *         *         *         *         *         *         *         *  2101>tttgttgtttccgggtgtacaatatggacttcctcttttctggcaaccaaacccatacatcgggattcctataataccttcgttggtctccctaacatgt>2200     1434<----------------------------------------------------------------------------------------------------<1434     1450<----------------------------------------------------------------------------------------------------<1450     1479<~~~~~~~~~~~~~~~~~~~~~~~~~~~~~~~~~~~~~~~~~~~~~~~~~~~~~~~~~~~~~~~~~~~~~~~~~~~~~~~~~~~~~~~~~~~~~~~~~~~~<1479     1395<~~~~~~~~~~~~~~~~~~~~~~~~~~~~~~~~~~~~~~~~~~~~~~~~~~~~~~~~~~~~~~~~~~~~~~~~~~~~~~~~~~~~~~~~~~~~~~~~~~~~<1395     1388<~~~~~~~~~~~~~~~~~~~~~~~~~~~~~~~~~~~~~~~~~~~~~~~~~~~~~~~~~~~~~~~~~~~~~~~~~~~~~~~~~~~~~~~~~~~~~~~~~~~~<1388     1395<~~~~~~~~~~~~~~~~~~~~~~~~~~~~~~~~~~~~~~~~~~~~~~~~~~~~~~~~~~~~~~~~~~~~~~~~~~~~~~~~~~~~~~~~~~~~~~~~~~~~<1395                   *         *         *         *         *         *         *         *         *         *  2201>aggtggcggaggggagatatacaatagaacagataccagacaagacataatgggctaaacaagactacaccaattacactgcctcattgatggtggtaca>2300     1434<----------------------------------------------------------------------------------------------------<1434     1450<----------------------------------------------------------------------------------------------------<1450     1479<~~~~~~~~~~~~~~~~~~~~~~~~~~~~~~~~~~~~~~~~~~~~~~~~~~~~~~~~~~~~~~~~~~~~~~~~~~~~~~~~~~~~~~~~~~~~~~~~~~~~<1479     1395<~~~~~~~~~~~~~~~~~~~~~~~~~~~~~~~~~~~~~~~~~~~~~~~~~~~~~~~~~~~~~~~~~~~~~~~~~~~~~~~~~~~~~~~~~~~~~~~~~~~~<1395     1388<~~~~~~~~~~~~~~~~~~~~~~~~~~~~~~~~~~~~~~~~~~~~~~~~~~~~~~~~~~~~~~~~~~~~~~~~~~~~~~~~~~~~~~~~~~~~~~~~~~~~<1388     1395<~~~~~~~~~~~~~~~~~~~~~~~~~~~~~~~~~~~~~~~~~~~~~~~~~~~~~~~~~~~~~~~~~~~~~~~~~~~~~~~~~~~~~~~~~~~~~~~~~~~~<1395                   *         *         *         *         *         *         *         *         *         *  2301>taacgaactaatactgtagccctagacttgatagccatcatcatatcgaagtttcactaccctttttccatttgccatctattgaagtaataataggcgc>2400     1434<----------------------------------------------------------------------------------------------------<1434     1450<----------------------------------------------------------------------------------------------------<1450     1479<~~~~~~~~~~~~~~~~~~~~~~~~~~~~~~~~~~~~~~~~~~~~~~~~~~~~~~~~~~~~~~~~~~~~~~~~~~~~~~~~~~~~~~~~~~~~~~~~~~~~<1479     1395<~~~~~~~~~~~~~~~~~~~~~~~~~~~~~~~~~~~~~~~~~~~~~~~~~~~~~~~~~~~~~~~~~~~~~~~~~~~~~~~~~~~~~~~~~~~~~~~~~~~~<1395     1388<~~~~~~~~~~~~~~~~~~~~~~~~~~~~~~~~~~~~~~~~~~~~~~~~~~~~~~~~~~~~~~~~~~~~~~~~~~~~~~~~~~~~~~~~~~~~~~~~~~~~<1388     1395<~~~~~~~~~~~~~~~~~~~~~~~~~~~~~~~~~~~~~~~~~~~~~~~~~~~~~~~~~~~~~~~~~~~~~~~~~~~~~~~~~~~~~~~~~~~~~~~~~~~~<1395                   *         *         *         *         *         *         *         *         *         *  2401>atgcaacttcttttctttttttttcttttctctctcccccgttgttgtctcaccatatccgcaatgacaaaaaaatgatggaagacactaaaggaaaaaa>2500     1434<----------------------------------------------------------------------------------------------------<1434     1450<----------------------------------------------------------------------------------------------------<1450     1479<~~~~~~~~~~~~~~~~~~~~~~~~~~~~~~~~~~~~~~~~~~~~~~~~~~~~~~~~~~~~~~~~~~~~~~~~~~~~~~~~~~~~~~~~~~~~~~~~~~~~<1479     1395<~~~~~~~~~~~~~~~~~~~~~~~~~~~~~~~~~~~~~~~~~~~~~~~~~~~~~~~~~~~~~~~~~~~~~~~~~~~~~~~~~~~~~~~~~~~~~~~~~~~~<1395     1388<~~~~~~~~~~~~~~~~~~~~~~~~~~~~~~~~~~~~~~~~~~~~~~~~~~~~~~~~~~~~~~~~~~~~~~~~~~~~~~~~~~~~~~~~~~~~~~~~~~~~<1388     1395<~~~~~~~~~~~~~~~~~~~~~~~~~~~~~~~~~~~~~~~~~~~~~~~~~~~~~~~~~~~~~~~~~~~~~~~~~~~~~~~~~~~~~~~~~~~~~~~~~~~~<1395                   *         *         *         *         *         *         *         *         *         *  2501>ttaacgacaaagacagcaccaacagatgtcgttgttccagagctgatgaggggtatctcgaagcacacgaaactttttccttccttcattcacgcacact>2600     1434<----------------------------------------------------------------------------------------------------<1434     1450<----------------------------------------------------------------------------------------------------<1450     1479<~~~~~~~~~~~~~~~~~~~~~~~~~~~~~~~~~~~~~~~~~~~~~~~~~~~~~~~~~~~~~~~~~~~~~~~~~~~~~~~~~~~~~~~~~~~~~~~~~~~~<1479     1395<~~~~~~~~~~~~~~~~~~~~~~~~~~~~~~~~~~~~~~~~~~~~~~~~~~~~~~~~~~~~~~~~~~~~~~~~~~~~~~~~~~~~~~~~~~~~~~~~~~~~<1395     1388<~~~~~~~~~~~~~~~~~~~~~~~~~~~~~~~~~~~~~~~~~~~~~~~~~~~~~~~~~~~~~~~~~~~~~~~~~~~~~~~~~~~~~~~~~~~~~~~~~~~~<1388     1395<~~~~~~~~~~~~~~~~~~~~~~~~~~~~~~~~~~~~~~~~~~~~~~~~~~~~~~~~~~~~~~~~~~~~~~~~~~~~~~~~~~~~~~~~~~~~~~~~~~~~<1395                   *         *         *         *         *         *         *         *         *         *  2601>actctctaatgagcaacggtatacggccttccttccagttacttgaatttgaaataaaaaaaagtttgctgtcttgctatcaagtataaatagacctgca>2700     1434<----------------------------------------------------------------------------------------------------<1434     1450<----------------------------------------------------------------------------------------------------<1450     1479<~~~~~~~~~~~~~~~~~~~~~~~~~~~~~~~~~~~~~~~~~~~~~~~~~~~~~~~~~~~~~~~~~~~~~~~~~~~~~~~~~~~~~~~~~~~~~~~~~~~~<1479     1395<~~~~~~~~~~~~~~~~~~~~~~~~~~~~~~~~~~~~~~~~~~~~~~~~~~~~~~~~~~~~~~~~~~~~~~~~~~~~~~~~~~~~~~~~~~~~~~~~~~~~<1395     1388<~~~~~~~~~~~~~~~~~~~~~~~~~~~~~~~~~~~~~~~~~~~~~~~~~~~~~~~~~~~~~~~~~~~~~~~~~~~~~~~~~~~~~~~~~~~~~~~~~~~~<1388     1395<~~~~~~~~~~~~~~~~~~~~~~~~~~~~~~~~~~~~~~~~~~~~~~~~~~~~~~~~~~~~~~~~~~~~~~~~~~~~~~~~~~~~~~~~~~~~~~~~~~~~<1395                   *         *         *         *         *         *         *         *         *         *  2701>attattaatcttttgtttcctcgtcattgttctcgttccctttcttccttgtttctttttctgcacaatatttcaagctataccaagcatacaatcaact>2800     1434<----------------------------------------------------------------------------------------------------<1434     1450<----------------------------------------------------------------------------------------------------<1450     1479<~~~~~~~~~~~~~~~~~~~~~~~~~~~~~~~~~~~~~~~~~~~~~~~~~~~~~~~~~~~~~~~~~~~~~~~~~~~~~~~~~~~~~~~~~~~~~~~~~~~~<1479     1395<~~~~~~~~~~~~~~~~~~~~~~~~~~~~~~~~~~~~~~~~~~~~~~~~~~~~~~~~~~~~~~~~~~~~~~~~~~~~~~~~~~~~~~~~~~~~~~~~~~~~<1395     1388<~~~~~~~~~~~~~~~~~~~~~~~~~~~~~~~~~~~~~~~~~~~~~~~~~~~~~~~~~~~~~~~~~~~~~~~~~~~~~~~~~~~~~~~~~~~~~~~~~~~~<1388     1395<~~~~~~~~~~~~~~~~~~~~~~~~~~~~~~~~~~~~~~~~~~~~~~~~~~~~~~~~~~~~~~~~~~~~~~~~~~~~~~~~~~~~~~~~~~~~~~~~~~~~<1395                   *         *         *         *         *         *         *         *         *         *  2801>atctcatatacatctagaactagtggatcccccatcacaagtttgtacaaaaaagcaggcttcaaaatgaaagctccttcatcaaatggagtttctccta>2900     1434<----------------------------------------------------------------------------------------------------<1434     1450<----------------------------------------------------------------------------------------------------<1450     1479<~~~~~~~~~~~~~~~~~~~~~~~~~~~~~~~~~~~~~~~~~~~~~~~~~~~~~~~~~~~~~~~~~~~~~~~~~~~~~~~~~~~~~~~~~~~~~~~~~~~~<1479     1395<~~~~~~~~~~~~~~~~~~~~~~~~~~~~~~~~~~~~~~~~~~~~~~~~~~~~~~~~~~~~~~~~~~~~~~~~~~~~~~~~~~~~~~~~~~~~~~~~~~~~<1395     1388<~~~~~~~~~~~~~~~~~~~~~~~~~~~~~~~~~~~~~~~~~~~~~~~~~~~~~~~~~~~~~~~~~~~~~~~~~~~~~~~~~~~~~~~~~~~~~~~~~~~~<1388     1395<~~~~~~~~~~~~~~~~~~~~~~~~~~~~~~~~~~~~~~~~~~~~~~~~~~~~~~~~~~~~~~~~~~~~~~~~~~~~~~~~~~~~~~~~~~~~~~~~~~~~<1395                   *         *         *         *         *         *         *         *         *         *  2901>atcctgttgaaggagaaaggagaaatataaactcagagctatggcacgcttgtgctgggccattgatttcgttgcctccagcaggaagtcttgttgttta>3000     1434<----------------------------------------------------------------------------------------------------<1434     1450<----------------------------------------------------------------------------------------------------<1450     1479<~~~~~~~~~~~~~~~~~~~~~~~~~~~~~~~~~~~~~~~~~~~~~~~~~~~~~~~~~~~~~~~~~~~~~~~~~~~~~~~~~~~~~~~~~~~~~~~~~~~~<1479     1395<~~~~~~~~~~~~~~~~~~~~~~~~~~~~~~~~~~~~~~~~~~~~~~~~~~~~~~~~~~~~~~~~~~~~~~~~~~~~~~~~~~~~~~~~~~~~~~~~~~~~<1395     1388<~~~~~~~~~~~~~~~~~~~~~~~~~~~~~~~~~~~~~~~~~~~~~~~~~~~~~~~~~~~~~~~~~~~~~~~~~~~~~~~~~~~~~~~~~~~~~~~~~~~~<1388     1395<~~~~~~~~~~~~~~~~~~~~~~~~~~~~~~~~~~~~~~~~~~~~~~~~~~~~~~~~~~~~~~~~~~~~~~~~~~~~~~~~~~~~~~~~~~~~~~~~~~~~<1395                   *         *         *         *         *         *         *         *         *         *  3001>cttccctcaaggtcacagtgagcaagtcgcggcttcaatgcagaagcagactgatttcataccaagttacccgaatcttccttccaagctcatatgcatg>3100     1434<----------------------------------------------------------------------------------------------------<1434     1450<----------------------------------------------------------------------------------------------------<1450     1479<~~~~~~~~~~~~~~~~~~~~~~~~~~~~~~~~~~~~~~~~~~~~~~~~~~~~~~~~~~~~~~~~~~~~~~~~~~~~~~~~~~~~~~~~~~~~~~~~~~~~<1479     1395<~~~~~~~~~~~~~~~~~~~~~~~~~~~~~~~~~~~~~~~~~~~~~~~~~~~~~~~~~~~~~~~~~~~~~~~~~~~~~~~~~~~~~~~~~~~~~~~~~~~~<1395     1388<~~~~~~~~~~~~~~~~~~~~~~~~~~~~~~~~~~~~~~~~~~~~~~~~~~~~~~~~~~~~~~~~~~~~~~~~~~~~~~~~~~~~~~~~~~~~~~~~~~~~<1388     1395<~~~~~~~~~~~~~~~~~~~~~~~~~~~~~~~~~~~~~~~~~~~~~~~~~~~~~~~~~~~~~~~~~~~~~~~~~~~~~~~~~~~~~~~~~~~~~~~~~~~~<1395                   *         *         *         *         *         *         *         *         *         *  3101>ctccacaatgttacactgaatgctgatcctgagacggatgaggtctatgcgcagatgactcttcagccagtaaacaaatatgacagagatgcattgcttg>3200     1434<----------------------------------------------------------------------------------------------------<1434     1450<----------------------------------------------------------------------------------------------------<1450     1479<~~~~~~~~~~~~~~~~~~~~~~~~~~~~~~~~~~~~~~~~~~~~~~~~~~~~~~~~~~~~~~~~~~~~~~~~~~~~~~~~~~~~~~~~~~~~~~~~~~~~<1479     1395<~~~~~~~~~~~~~~~~~~~~~~~~~~~~~~~~~~~~~~~~~~~~~~~~~~~~~~~~~~~~~~~~~~~~~~~~~~~~~~~~~~~~~~~~~~~~~~~~~~~~<1395     1388<~~~~~~~~~~~~~~~~~~~~~~~~~~~~~~~~~~~~~~~~~~~~~~~~~~~~~~~~~~~~~~~~~~~~~~~~~~~~~~~~~~~~~~~~~~~~~~~~~~~~<1388     1395<~~~~~~~~~~~~~~~~~~~~~~~~~~~~~~~~~~~~~~~~~~~~~~~~~~~~~~~~~~~~~~~~~~~~~~~~~~~~~~~~~~~~~~~~~~~~~~~~~~~~<1395                   *         *         *         *         *         *         *         *         *         *  3201>cttctgacatgggtcttaagctaaacagacaacctaatgaatttttctgcaaaaccctcacggcgagtgacacaagtactcacggtggattttctgtacc>3300     1434<----------------------------------------------------------------------------------------------------<1434     1450<----------------------------------------------------------------------------------------------------<1450     1479<~~~~~~~~~~~~~~~~~~~~~~~~~~~~~~~~~~~~~~~~~~~~~~~~~~~~~~~~~~~~~~~~~~~~~~~~~~~~~~~~~~~~~~~~~~~~~~~~~~~~<1479     1395<~~~~~~~~~~~~~~~~~~~~~~~~~~~~~~~~~~~~~~~~~~~~~~~~~~~~~~~~~~~~~~~~~~~~~~~~~~~~~~~~~~~~~~~~~~~~~~~~~~~~<1395     1388<~~~~~~~~~~~~~~~~~~~~~~~~~~~~~~~~~~~~~~~~~~~~~~~~~~~~~~~~~~~~~~~~~~~~~~~~~~~~~~~~~~~~~~~~~~~~~~~~~~~~<1388     1395<~~~~~~~~~~~~~~~~~~~~~~~~~~~~~~~~~~~~~~~~~~~~~~~~~~~~~~~~~~~~~~~~~~~~~~~~~~~~~~~~~~~~~~~~~~~~~~~~~~~~<1395                   *         *         *         *         *         *         *         *         *         *  3301>ccgacgagctgctgagaaaatctttcctgctctggatttctcgatgcaaccaccttgtcaggagcttgttgctaaggatattcatgacaacacatggact>3400     1434<----------------------------------------------------------------------------------------------------<1434     1450<----------------------------------------------------------------------------------------------------<1450     1479<~~~~~~~~~~~~~~~~~~~~~~~~~~~~~~~~~~~~~~~~~~~~~~~~~~~~~~~~~~~~~~~~~~~~~~~~~~~~~~~~~~~~~~~~~~~~~~~~~~~~<1479     1395<~~~~~~~~~~~~~~~~~~~~~~~~~~~~~~~~~~~~~~~~~~~~~~~~~~~~~~~~~~~~~~~~~~~~~~~~~~~~~~~~~~~~~~~~~~~~~~~~~~~~<1395     1388<~~~~~~~~~~~~~~~~~~~~~~~~~~~~~~~~~~~~~~~~~~~~~~~~~~~~~~~~~~~~~~~~~~~~~~~~~~~~~~~~~~~~~~~~~~~~~~~~~~~~<1388     1395<~~~~~~~~~~~~~~~~~~~~~~~~~~~~~~~~~~~~~~~~~~~~~~~~~~~~~~~~~~~~~~~~~~~~~~~~~~~~~~~~~~~~~~~~~~~~~~~~~~~~<1395                   *         *         *         *         *         *         *         *         *         *  3401>ttcagacatatttatcgaggtcaaccaaaaaggcacttgctaactacaggctggagtgtgtttgtcagcacgaaaaggctctttgctggagactctgttc>3500     1434<----------------------------------------------------------------------------------------------------<1434     1450<----------------------------------------------------------------------------------------------------<1450     1479<~~~~~~~~~~~~~~~~~~~~~~~~~~~~~~~~~~~~~~~~~~~~~~~~~~~~~~~~~~~~~~~~~~~~~~~~~~~~~~~~~~~~~~~~~~~~~~~~~~~~<1479     1395<~~~~~~~~~~~~~~~~~~~~~~~~~~~~~~~~~~~~~~~~~~~~~~~~~~~~~~~~~~~~~~~~~~~~~~~~~~~~~~~~~~~~~~~~~~~~~~~~~~~~<1395     1388<~~~~~~~~~~~~~~~~~~~~~~~~~~~~~~~~~~~~~~~~~~~~~~~~~~~~~~~~~~~~~~~~~~~~~~~~~~~~~~~~~~~~~~~~~~~~~~~~~~~~<1388     1395<~~~~~~~~~~~~~~~~~~~~~~~~~~~~~~~~~~~~~~~~~~~~~~~~~~~~~~~~~~~~~~~~~~~~~~~~~~~~~~~~~~~~~~~~~~~~~~~~~~~~<1395                   *         *         *         *         *         *         *         *         *         *  3501>tttttataagagatggaaaggcgcaacttctgttggggataagacgtgcaaatagacaacagcctgcactttcttcatctgtaatatcaagtgatagcat>3600     1434<----------------------------------------------------------------------------------------------------<1434     1450<----------------------------------------------------------------------------------------------------<1450     1479<~~~~~~~~~~~~~~~~~~~~~~~~~~~~~~~~~~~~~~~~~~~~~~~~~~~~~~~~~~~~~~~~~~~~~~~~~~~~~~~~~~~~~~~~~~~~~~~~~~~~<1479     1395<~~~~~~~~~~~~~~~~~~~~~~~~~~~~~~~~~~~~~~~~~~~~~~~~~~~~~~~~~~~~~~~~~~~~~~~~~~~~~~~~~~~~~~~~~~~~~~~~~~~~<1395     1388<~~~~~~~~~~~~~~~~~~~~~~~~~~~~~~~~~~~~~~~~~~~~~~~~~~~~~~~~~~~~~~~~~~~~~~~~~~~~~~~~~~~~~~~~~~~~~~~~~~~~<1388     1395<~~~~~~~~~~~~~~~~~~~~~~~~~~~~~~~~~~~~~~~~~~~~~~~~~~~~~~~~~~~~~~~~~~~~~~~~~~~~~~~~~~~~~~~~~~~~~~~~~~~~<1395                   *         *         *         *         *         *         *         *         *         *  3601>gcacatcggagttcttgcagctgcagctcatgctaatgctaataacagtcctttcaccattttctacaacccgaggtgggctgctcctgctgagtttgtg>3700     1434<----------------------------------------------------------------------------------------------------<1434     1450<----------------------------------------------------------------------------------------------------<1450     1479<~~~~~~~~~~~~~~~~~~~~~~~~~~~~~~~~~~~~~~~~~~~~~~~~~~~~~~~~~~~~~~~~~~~~~~~~~~~~~~~~~~~~~~~~~~~~~~~~~~~~<1479     1395<~~~~~~~~~~~~~~~~~~~~~~~~~~~~~~~~~~~~~~~~~~~~~~~~~~~~~~~~~~~~~~~~~~~~~~~~~~~~~~~~~~~~~~~~~~~~~~~~~~~~<1395     1388<~~~~~~~~~~~~~~~~~~~~~~~~~~~~~~~~~~~~~~~~~~~~~~~~~~~~~~~~~~~~~~~~~~~~~~~~~~~~~~~~~~~~~~~~~~~~~~~~~~~~<1388     1395<~~~~~~~~~~~~~~~~~~~~~~~~~~~~~~~~~~~~~~~~~~~~~~~~~~~~~~~~~~~~~~~~~~~~~~~~~~~~~~~~~~~~~~~~~~~~~~~~~~~~<1395                   *         *         *         *         *         *         *         *         *         *  3701>gttcctttagccaagtataccaaagcgatgtacgctcaagtttccctcggtatgcggtttagaatgatatttgagactgaagaatgtggagttcgtcggt>3800     1434<----------------------------------------------------------------------------------------------------<1434     1450<----------------------------------------------------------------------------------------------------<1450     1479<~~~~~~~~~~~~~~~~~~~~~~~~~~~~~~~~~~~~~~~~~~~~~~~~~~~~~~~~~~~~~~~~~~~~~~~~~~~~~~~~~~~~~~~~~~~~~~~~~~~~<1479     1395<~~~~~~~~~~~~~~~~~~~~~~~~~~~~~~~~~~~~~~~~~~~~~~~~~~~~~~~~~~~~~~~~~~~~~~~~~~~~~~~~~~~~~~~~~~~~~~~~~~~~<1395     1388<~~~~~~~~~~~~~~~~~~~~~~~~~~~~~~~~~~~~~~~~~~~~~~~~~~~~~~~~~~~~~~~~~~~~~~~~~~~~~~~~~~~~~~~~~~~~~~~~~~~~<1388     1395<~~~~~~~~~~~~~~~~~~~~~~~~~~~~~~~~~~~~~~~~~~~~~~~~~~~~~~~~~~~~~~~~~~~~~~~~~~~~~~~~~~~~~~~~~~~~~~~~~~~~<1395                   *         *         *         *         *         *         *         *         *         *  3801>atatgggtacagttaccggtatcagtgatcttgatccagtgagatggaaaaactctcagtggcggaatcttcagattggatgggatgagtcagctgctgg>3900     1434<----------------------------------------------------------------------------------------------------<1434     1450<----------------------------------------------------------------------------------------------------<1450     1479<~~~~~~~~~~~~~~~~~~~~~~~~~~~~~~~~~~~~~~~~~~~~~~~~~~~~~~~~~~~~~~~~~~~~~~~~~~~~~~~~~~~~~~~~~~~~~~~~~~~~<1479     1395<~~~~~~~~~~~~~~~~~~~~~~~~~~~~~~~~~~~~~~~~~~~~~~~~~~~~~~~~~~~~~~~~~~~~~~~~~~~~~~~~~~~~~~~~~~~~~~~~~~~~<1395     1388<~~~~~~~~~~~~~~~~~~~~~~~~~~~~~~~~~~~~~~~~~~~~~~~~~~~~~~~~~~~~~~~~~~~~~~~~~~~~~~~~~~~~~~~~~~~~~~~~~~~~<1388     1395<~~~~~~~~~~~~~~~~~~~~~~~~~~~~~~~~~~~~~~~~~~~~~~~~~~~~~~~~~~~~~~~~~~~~~~~~~~~~~~~~~~~~~~~~~~~~~~~~~~~~<1395                   *         *         *         *         *         *         *         *         *         *  3901>tgataggcccagtcgagtttcagtttgggacattgaaccggttttaactcctttctacatatgtcctcctccatttttccgacctcgcttttctggacaa>4000     1434<----------------------------------------------------------------------------------------------------<1434     1450<----------------------------------------------------------------------------------------------------<1450     1479<~~~~~~~~~~~~~~~~~~~~~~~~~~~~~~~~~~~~~~~~~~~~~~~~~~~~~~~~~~~~~~~~~~~~~~~~~~~~~~~~~~~~~~~~~~~~~~~~~~~~<1479     1395<~~~~~~~~~~~~~~~~~~~~~~~~~~~~~~~~~~~~~~~~~~~~~~~~~~~~~~~~~~~~~~~~~~~~~~~~~~~~~~~~~~~~~~~~~~~~~~~~~~~~<1395     1388<~~~~~~~~~~~~~~~~~~~~~~~~~~~~~~~~~~~~~~~~~~~~~~~~~~~~~~~~~~~~~~~~~~~~~~~~~~~~~~~~~~~~~~~~~~~~~~~~~~~~<1388     1395<~~~~~~~~~~~~~~~~~~~~~~~~~~~~~~~~~~~~~~~~~~~~~~~~~~~~~~~~~~~~~~~~~~~~~~~~~~~~~~~~~~~~~~~~~~~~~~~~~~~~<1395                   *         *         *         *         *         *         *         *         *         *  4001>cctggaatgccagatgatgagactgacatggagtctgcactgaagagagcaatgccatggcttgataatagcttagagatgaaagacccttcgagtacta>4100     1434<----------------------------------------------------------------------------------------------------<1434     1450<----------------------------------------------------------------------------------------------------<1450     1479<~~~~~~~~~~~~~~~~~~~~~~~~~~~~~~~~~~~~~~~~~~~~~~~~~~~~~~~~~~~~~~~~~~~~~~~~~~~~~~~~~~~~~~~~~~~~~~~~~~~~<1479     1395<~~~~~~~~~~~~~~~~~~~~~~~~~~~~~~~~~~~~~~~~~~~~~~~~~~~~~~~~~~~~~~~~~~~~~~~~~~~~~~~~~~~~~~~~~~~~~~~~~~~~<1395     1388<~~~~~~~~~~~~~~~~~~~~~~~~~~~~~~~~~~~~~~~~~~~~~~~~~~~~~~~~~~~~~~~~~~~~~~~~~~~~~~~~~~~~~~~~~~~~~~~~~~~~<1388     1395<~~~~~~~~~~~~~~~~~~~~~~~~~~~~~~~~~~~~~~~~~~~~~~~~~~~~~~~~~~~~~~~~~~~~~~~~~~~~~~~~~~~~~~~~~~~~~~~~~~~~<1395                   *         *         *         *         *         *         *         *         *         *  4101>tctttcctggtctgagtttagttcagtggatgaatatgcagcagcagaacggccagctaccctctgctgctgcacagccaggtttcttcccatcaatgct>4200     1434<----------------------------------------------------------------------------------------------------<1434     1450<----------------------------------------------------------------------------------------------------<1450     1479<~~~~~~~~~~~~~~~~~~~~~~~~~~~~~~~~~~~~~~~~~~~~~~~~~~~~~~~~~~~~~~~~~~~~~~~~~~~~~~~~~~~~~~~~~~~~~~~~~~~~<1479     1395<~~~~~~~~~~~~~~~~~~~~~~~~~~~~~~~~~~~~~~~~~~~~~~~~~~~~~~~~~~~~~~~~~~~~~~~~~~~~~~~~~~~~~~~~~~~~~~~~~~~~<1395     1388<~~~~~~~~~~~~~~~~~~~~~~~~~~~~~~~~~~~~~~~~~~~~~~~~~~~~~~~~~~~~~~~~~~~~~~~~~~~~~~~~~~~~~~~~~~~~~~~~~~~~<1388     1395<~~~~~~~~~~~~~~~~~~~~~~~~~~~~~~~~~~~~~~~~~~~~~~~~~~~~~~~~~~~~~~~~~~~~~~~~~~~~~~~~~~~~~~~~~~~~~~~~~~~~<1395                   *         *         *         *         *         *         *         *         *         *  4201>ttcgccaaccgcggcgctgcacaacaatcttggcggcactgatgatccctccaagttactgagctttcagacgccgcacggggggatttcctcctcaaat>4300     1434<----------------------------------------------------------------------------------------------------<1434     1450<----------------------------------------------------------------------------------------------------<1450     1479<~~~~~~~~~~~~~~~~~~~~~~~~~~~~~~~~~~~~~~~~~~~~~~~~~~~~~~~~~~~~~~~~~~~~~~~~~~~~~~~~~~~~~~~~~~~~~~~~~~~~<1479     1395<~~~~~~~~~~~~~~~~~~~~~~~~~~~~~~~~~~~~~~~~~~~~~~~~~~~~~~~~~~~~~~~~~~~~~~~~~~~~~~~~~~~~~~~~~~~~~~~~~~~~<1395     1388<~~~~~~~~~~~~~~~~~~~~~~~~~~~~~~~~~~~~~~~~~~~~~~~~~~~~~~~~~~~~~~~~~~~~~~~~~~~~~~~~~~~~~~~~~~~~~~~~~~~~<1388     1395<~~~~~~~~~~~~~~~~~~~~~~~~~~~~~~~~~~~~~~~~~~~~~~~~~~~~~~~~~~~~~~~~~~~~~~~~~~~~~~~~~~~~~~~~~~~~~~~~~~~~<1395                   *         *         *         *         *         *         *         *         *         *  4301>ctccaatttaacaaacagaatcagcaagccccaatgtctcagttgcctcagccaccaactacgttgtcccaacaacagcagctgcagcaattgttgcact>4400     1434<----------------------------------------------------------------------------------------------------<1434     1450<----------------------------------------------------------------------------------------------------<1450     1479<~~~~~~~~~~~~~~~~~~~~~~~~~~~~~~~~~~~~~~~~~~~~~~~~~~~~~~~~~~~~~~~~~~~~~~~~~~~~~~~~~~~~~~~~~~~~~~~~~~~~<1479     1395<~~~~~~~~~~~~~~~~~~~~~~~~~~~~~~~~~~~~~~~~~~~~~~~~~~~~~~~~~~~~~~~~~~~~~~~~~~~~~~~~~~~~~~~~~~~~~~~~~~~~<1395     1388<~~~~~~~~~~~~~~~~~~~~~~~~~~~~~~~~~~~~~~~~~~~~~~~~~~~~~~~~~~~~~~~~~~~~~~~~~~~~~~~~~~~~~~~~~~~~~~~~~~~~<1388     1395<~~~~~~~~~~~~~~~~~~~~~~~~~~~~~~~~~~~~~~~~~~~~~~~~~~~~~~~~~~~~~~~~~~~~~~~~~~~~~~~~~~~~~~~~~~~~~~~~~~~~<1395                   *         *         *         *         *         *         *         *         *         *  4401>cctctttgaaccatcaacaacagcaatcgcagtctcaacaacagcaacaacaacaacagttgctgcagcagcaacaacaattgcagtctcaacaacacag>4500     1434<----------------------------------------------------------------------------------------------------<1434     1450<----------------------------------------------------------------------------------------------------<1450     1479<~~~~~~~~~~~~~~~~~~~~~~~~~~~~~~~~~~~~~~~~~~~~~~~~~~~~~~~~~~~~~~~~~~~~~~~~~~~~~~~~~~~~~~~~~~~~~~~~~~~~<1479     1395<~~~~~~~~~~~~~~~~~~~~~~~~~~~~~~~~~~~~~~~~~~~~~~~~~~~~~~~~~~~~~~~~~~~~~~~~~~~~~~~~~~~~~~~~~~~~~~~~~~~~<1395     1388<~~~~~~~~~~~~~~~~~~~~~~~~~~~~~~~~~~~~~~~~~~~~~~~~~~~~~~~~~~~~~~~~~~~~~~~~~~~~~~~~~~~~~~~~~~~~~~~~~~~~<1388     1395<~~~~~~~~~~~~~~~~~~~~~~~~~~~~~~~~~~~~~~~~~~~~~~~~~~~~~~~~~~~~~~~~~~~~~~~~~~~~~~~~~~~~~~~~~~~~~~~~~~~~<1395                   *         *         *         *         *         *         *         *         *         *  4501>caacaacaatcaatcgcagtctcagcaacaacaacaattgctccagcagcaacaacaacaacaactgcagcaacaacatcaacaaccgttacagcaacag>4600     1434<----------------------------------------------------------------------------------------------------<1434     1450<----------------------------------------------------------------------------------------------------<1450     1479<~~~~~~~~~~~~~~~~~~~~~~~~~~~~~~~~~~~~~~~~~~~~~~~~~~~~~~~~~~~~~~~~~~~~~~~~~~~~~~~~~~~~~~~~~~~~~~~~~~~~<1479     1395<~~~~~~~~~~~~~~~~~~~~~~~~~~~~~~~~~~~~~~~~~~~~~~~~~~~~~~~~~~~~~~~~~~~~~~~~~~~~~~~~~~~~~~~~~~~~~~~~~~~~<1395     1388<~~~~~~~~~~~~~~~~~~~~~~~~~~~~~~~~~~~~~~~~~~~~~~~~~~~~~~~~~~~~~~~~~~~~~~~~~~~~~~~~~~~~~~~~~~~~~~~~~~~~<1388     1395<~~~~~~~~~~~~~~~~~~~~~~~~~~~~~~~~~~~~~~~~~~~~~~~~~~~~~~~~~~~~~~~~~~~~~~~~~~~~~~~~~~~~~~~~~~~~~~~~~~~~<1395                   *         *         *         *         *         *         *         *         *         *  4601>actcagcagcagcagctaagaacacagccattgcaatctcactcgcatccacagccacaacagttacaacaacataagttgcagcaacttcaggttccac>4700     1434<----------------------------------------------------------------------------------------------------<1434     1450<----------------------------------------------------------------------------------------------------<1450     1479<~~~~~~~~~~~~~~~~~~~~~~~~~~~~~~~~~~~~~~~~~~~~~~~~~~~~~~~~~~~~~~~~~~~~~~~~~~~~~~~~~~~~~~~~~~~~~~~~~~~~<1479     1395<~~~~~~~~~~~~~~~~~~~~~~~~~~~~~~~~~~~~~~~~~~~~~~~~~~~~~~~~~~~~~~~~~~~~~~~~~~~~~~~~~~~~~~~~~~~~~~~~~~~~<1395     1388<~~~~~~~~~~~~~~~~~~~~~~~~~~~~~~~~~~~~~~~~~~~~~~~~~~~~~~~~~~~~~~~~~~~~~~~~~~~~~~~~~~~~~~~~~~~~~~~~~~~~<1388     1395<~~~~~~~~~~~~~~~~~~~~~~~~~~~~~~~~~~~~~~~~~~~~~~~~~~~~~~~~~~~~~~~~~~~~~~~~~~~~~~~~~~~~~~~~~~~~~~~~~~~~<1395                   *         *         *         *         *         *         *         *         *         *  4701>agaatcagctttacaatggtcaacaagcagcgcagcagcatcagtcgcaacaagcatctacacatcatttgcaaccacaattagtttcgggatcaatggc>4800     1434<----------------------------------------------------------------------------------------------------<1434     1450<----------------------------------------------------------------------------------------------------<1450     1479<~~~~~~~~~~~~~~~~~~~~~~~~~~~~~~~~~~~~~~~~~~~~~~~~~~~~~~~~~~~~~~~~~~~~~~~~~~~~~~~~~~~~~~~~~~~~~~~~~~~~<1479     1395<~~~~~~~~~~~~~~~~~~~~~~~~~~~~~~~~~~~~~~~~~~~~~~~~~~~~~~~~~~~~~~~~~~~~~~~~~~~~~~~~~~~~~~~~~~~~~~~~~~~~<1395     1388<~~~~~~~~~~~~~~~~~~~~~~~~~~~~~~~~~~~~~~~~~~~~~~~~~~~~~~~~~~~~~~~~~~~~~~~~~~~~~~~~~~~~~~~~~~~~~~~~~~~~<1388     1395<~~~~~~~~~~~~~~~~~~~~~~~~~~~~~~~~~~~~~~~~~~~~~~~~~~~~~~~~~~~~~~~~~~~~~~~~~~~~~~~~~~~~~~~~~~~~~~~~~~~~<1395                   *         *         *         *         *         *         *         *         *         *  4801>aagcagtgtcatcacgcctccgtccagctcccttaatcaaagctttcaacagcaacaacaacagtctaagcaacttcaacaagcacatcaccatttaggt>4900     1434<----------------------------------------------------------------------------------------------------<1434     1450<----------------------------------------------------------------------------------------------------<1450     1479<~~~~~~~~~~~~~~~~~~~~~~~~~~~~~~~~~~~~~~~~~~~~~~~~~~~~~~~~~~~~~~~~~~~~~~~~~~~~~~~~~~~~~~~~~~~~~~~~~~~~<1479     1395<~~~~~~~~~~~~~~~~~~~~~~~~~~~~~~~~~~~~~~~~~~~~~~~~~~~~~~~~~~~~~~~~~~~~~~~~~~~~~~~~~~~~~~~~~~~~~~~~~~~~<1395     1388<~~~~~~~~~~~~~~~~~~~~~~~~~~~~~~~~~~~~~~~~~~~~~~~~~~~~~~~~~~~~~~~~~~~~~~~~~~~~~~~~~~~~~~~~~~~~~~~~~~~~<1388     1395<~~~~~~~~~~~~~~~~~~~~~~~~~~~~~~~~~~~~~~~~~~~~~~~~~~~~~~~~~~~~~~~~~~~~~~~~~~~~~~~~~~~~~~~~~~~~~~~~~~~~<1395                   *         *         *         *         *         *         *         *         *         *  4901>gctagcactagccagagtagtgtaattgaaaccagcaagtcttcatccaatctgatgtccgcaccgccgcaagagacacagttttcacgacaagtagaac>5000     1434<----------------------------------------------------------------------------------------------------<1434     1450<----------------------------------------------------------------------------------------------------<1450     1479<~~~~~~~~~~~~~~~~~~~~~~~~~~~~~~~~~~~~~~~~~~~~~~~~~~~~~~~~~~~~~~~~~~~~~~~~~~~~~~~~~~~~~~~~~~~~~~~~~~~~<1479     1395<~~~~~~~~~~~~~~~~~~~~~~~~~~~~~~~~~~~~~~~~~~~~~~~~~~~~~~~~~~~~~~~~~~~~~~~~~~~~~~~~~~~~~~~~~~~~~~~~~~~~<1395     1388<~~~~~~~~~~~~~~~~~~~~~~~~~~~~~~~~~~~~~~~~~~~~~~~~~~~~~~~~~~~~~~~~~~~~~~~~~~~~~~~~~~~~~~~~~~~~~~~~~~~~<1388     1395<~~~~~~~~~~~~~~~~~~~~~~~~~~~~~~~~~~~~~~~~~~~~~~~~~~~~~~~~~~~~~~~~~~~~~~~~~~~~~~~~~~~~~~~~~~~~~~~~~~~~<1395                   *         *         *         *         *         *         *         *         *         *  5001>agcagcagcctcctggtctcaacgggcagaatcagcaaacacttttgcagcagaaagctcaccaggcacaggcccaacagatattccagcagagtctctt>5100     1434<----------------------------------------------------------------------------------------------------<1434     1450<----------------------------------------------------------------------------------------------------<1450     1479<~~~~~~~~~~~~~~~~~~~~~~~~~~~~~~~~~~~~~~~~~~~~~~~~~~~~~~~~~~~~~~~~~~~~~~~~~~~~~~~~~~~~~~~~~~~~~~~~~~~~<1479     1395<~~~~~~~~~~~~~~~~~~~~~~~~~~~~~~~~~~~~~~~~~~~~~~~~~~~~~~~~~~~~~~~~~~~~~~~~~~~~~~~~~~~~~~~~~~~~~~~~~~~~<1395     1388<~~~~~~~~~~~~~~~~~~~~~~~~~~~~~~~~~~~~~~~~~~~~~~~~~~~~~~~~~~~~~~~~~~~~~~~~~~~~~~~~~~~~~~~~~~~~~~~~~~~~<1388     1395<~~~~~~~~~~~~~~~~~~~~~~~~~~~~~~~~~~~~~~~~~~~~~~~~~~~~~~~~~~~~~~~~~~~~~~~~~~~~~~~~~~~~~~~~~~~~~~~~~~~~<1395                   *         *         *         *         *         *         *         *         *         *  5101>ggaacagccgcatatacagtttcagctgttacagagattacaacagcaacagcagcagcaatttctttcgccgcagtctcagttaccacaccatcaattg>5200     1434<----------------------------------------------------------------------------------------------------<1434     1449<-----------ACACGCAGTCTGTTACCAGAGAGATTTCAACGGCAAACGGCAGGCAGCATTCTCGCCGCCAGTTTCAAGTTTAAGCACCCATCAATTGC<1361     1479<~~~~~~~~~~~~~~~~~~~~~~~~~~~~~~~~~~~~~~~~~~~~~~~~~~~~~~~~~~~~~~~~~~~~~~~~~~~~~~~~~~~~~~~~~~~~~~~~~~~~<1479     1395<~~~~~~~~~~~~~~~~~~~~~~~~~~~~~~~~~~~~~~~~~~~~~~~~~~~~~~~~~~~~~~~~~~~~~~~~~~~~~~~~~~~~~~~~~~~~~~~~~~~~<1395     1387<~~~~~~~~~~~~~~~~~~~~~~~~~~~~~~~~~~~~~~~~~~~~~~~~~~~~~~~~~~~~~~~~~~~~~~GGCAGCAATTTCTTGACCGCAAGTTCCAGT<1358     1395<~~~~~~~~~~~~~~~~~~~~~~~~~~~~~~~~~~~~~~~~~~~~~~~~~~~~~~~~~~~~~~~~~~~~~~~~~~~~~~~~~~~~~~~~~~~~~~~~~~~~<1395                   *         *         *         *         *         *         *         *         *            5201>caaagccagcagttgcaacagctgcctactctctctcaaggtcatcagtttccgtcatcttgcactaacaatggcttatcgacgttgcaaccacct-caa>5299     1433<----------------------------------------------------------------------GCCACACCACCTCTCAAATGTCGTGT-TGT<1405     1360<CAAGCCAGCAGTTGCAACCAGCTGCTACTTTTCTCTCAAAGGTCATCAGTTCGTCATTCTTGCACTAACAATGGCTTATCGACGT-GCAGCCACCTCCAA<1262     1479<~~~~~~~~~~~~~~~~~~~~~~~~~~~~~~~~~~~~~~~~~~~~~~~~~~~~~~~~~~~~~~~~~~~~~~~~~~~~~~~~~~~~~~~~~~~~~~~~~~~~<1479     1395<~~~~~~~~~~~~~~~~~~~~~~~~~~~~~~~~~~~~~~~~~~~~~~~~~~~~~~~~~~~~~~~~~~~~~~~~~~~~~~~~~~~~~~~~~~~~~~~~~~~~<1395     1357<TACCAACCATTCATTGCAAGGCAGCAGTGCAACAGGTGCCTATCTTTCTTCCAGGTCATCAGTTCGTCATCTGCACTAACAATGCTATCGACGTGC-AAT<1259     1395<~~~~~~~~~~~~~~~~~~~~~~~~~~~~~~~~~~~~~~~~~~~~~~~~~~~~~~~~~~~~~~~~~~~~~~~~~~~~~~~~~~~~~~~~~~~~~~~~~~~~<1395          *          *         *         *         *         *         *         *         *         *          5300>atgctggt-gagccgacctcaggaaaaacaaaacccaccggttgggggaggggtcaaagcttattcaggcatcacagatggaggagatgcaccttcctct>5398     1404<AGAGCGCG-ACCTCTCAGGAAAAAACAAAACCCACCCGGTTGGGGGAGGGTCAAGCTTATTCAGCATCACCAGAATTGAGGGAGAGATGGCACCTCCTCT<1306     1261<ATGCTGGTGGAGCCGA-CTCA-GAAAAACAAAACCCACCGGTTGGGGGAGGGGTCAAAGCTTATTCAGGCATCACAGATG-AGGAGATGCACCTTCCTC-<1166     1478<~~~~~~~~~~~~~~~~~~~~~~~~~~~~~~~~~~~~~~~~~~~~~~~~~~~~~~~~~~~~~~~~~~~~~~~~~~~~AACAAATAGTTGGCGCCTCTTGCA<1455     1394<~~~~~~~~~~~~~~~~~CGAAAAACGGTAATTCAGTATCGAAGCGGGTGTCGTGGCTGTCAACAAGGCTGATCAAGTCTTGACATATCTCATCAGATAGC<1312     1258<CACTCAAT-GCTGTGAGCGACTCAGAAAAAACAAAATCCACCGTTGGGGGAGGGGGTCAAGCTATCAGCCATCACAGATGGAGGAGATGCAC--T-CTC-<1164     1394<~~~~~~~~~~~~~~~~~GTATCAACATTCCACAGTGTGTTATTGAGGGAAGTATCAGTACTCGAAACCGTGCTCGTGCCTTACAGCTGATCAGTTCTGAC<1312           *         *         *         *         *         *         *         *         *         *          5399>tcaacgtcgccttccaccaacaactgtcagatctcttcttcaggctttctcaacagaagccaaagcgggccagcgatcttgatacctgatgcagcgattg>5498     1305<TCACGTCGCCTTCCACCAACAACTGTCAGAATCTCTTCTTCAGGCTTTCTCAACAGAAGCCAAAGCGG-CCAGCGATC-TGATA-CTGATGCAGCGATTG<1209     1165<TCAACGTCGCCTTCCACCAACAACTGTCAGATCTCTTCTTCAGGCTTTCTCAACAGAAGCCAAAGCGGGCCAGCGATCTTGATACCTGATGCAGCGATTG<1066     1454<CACACAACAACCGTCAGAATTCTCTTTTCAAGCTTTCACAAACAGGAGGCCAAAGCGGCAAGCGATTCTTGAATACCTGAGTGGCAGGCGGATGATATGT<1355     1311<CAGCACAGTCTGATGCAATTAATTGAATTCTCTTTTGCTGGAACTGATGAGGCTGACATCACCAAGTTCTCTTCTGCAGTCTTCTGCGATCATACAAGCA<1212     1163<TCAACGTCGCCTTCCACCAACAACTGTCAGATCTCTTCT-CAGGCTTTCTCAACAGAAGCCAAAGCGGGCCAGCGATCT-GATACCTGATGCAGCGATTG<1066     1311<CATACTCATCAGATACACCACAGTCTGATCAATAAATTGATCTCTTCTGAAACTGATGAGGCTGACATCACAGTCTCTCTGCAGTCTCTGCCGATCATAC<1212           *          *         *         *         *         *         *         *         *         *         5499>atatg-tctggtaatcttgttcaggatctttacagcaaatccgatatgcggctaaaacaagaactcgtgggtcagcaaaagtccaaagctagtttaacag>5597     1208<ATATGTTCTGGTAATC-TG-TCAGGATC-TTACAGCAAATCCGATATGCGGCTAAAACAAGAACTCGTGGGTCAGCAAAAGTCCAAAGCTAGTTTAACAG<1112     1065<ATATG-TCTGGTAATCTTGTTCAGGATCTTTACAGCAAATCCGATATGCGGCTAAAACAAGAACTCGTGGGTCAGCAAAAGTCCAAAGCTAGTTTAACAG<967      1354<TCTGG-TAAATTCTTGTTCAGGATCCTTACAGCAAATCCCGAATATTGCGGCTAAACAAGAACTCCGTGGGTCAGCAAAAGTCCAAAGCTAGTT-AACAG<1257     1211<ACATC-ATTCTATCTCCCAGGATCTCAGCTGTTGTGTTACCGGATGCACAAACTCACCGCTGTTTCATGATGTGTGGGACACTCAGTGAACGGTCTCAAG<1113     1065<ATATG-TCTGGTAATCTTGTTCAGGATCTTTACAGCAAATCCGATATGCGGCTAAAACAAGAACTCGTGGGTCAGCAAAAGTCCAAAGCTAGTTTAACAG<967      1211<AAGCA-GCATCATCTATCTCCCAGGATCTCAGCTGTGTGTACGGATTCCACAAACTCACGCTGTTCATGATGTGTGGGACACTCAGTTGAACGGTCTCAA<1113            *         *         *          *         *         *          *         *         *         *       5598>atcatcaactagaagcatctgcctc-tggaacttcttacggtttagatggaggcgaaaacaac-agacaacaaaatttcttggctccaacttttggcctt>5695     1111<ATCATCAACTAGAAGCATCTGCCTC-TGGAACTTCTTACGGTTTAGATGGAGGCGAAAACAAC-AGACAACAAAATTTCTTGGCTCCAACTTTTGGCCTT<1014      966<ATCATCAACTAGAAGCATCTGCCTC-TGGAACTTCTTACGGTTTAGATGGAGGCGAAAACAAC-AGACAACAAAATTTCTTGGCTCCAACTTTTGGCCTT<869      1256<ATCATCAACTAGAAGCATCTGCCTCCTGGAACTTCTTACGGTTTAGATG-A-GCGAAAACAACAAGACAACAAAATTTCTTGGCTCCAACTTTTGGCCTT<1159     1112<TTTGACCAGTTCAGTCCCTGATGCA-GCAGGACCTTATGCTAGTCAGAATATCTGTATGAGTA-ATAGCACAACCAGTAACATTCTAGATCCTCCACTCT<1015      966<ATCATCAACTAGAAGCATCTGCCTC-TGGAACTTCTTACGGTTTAGATGGAGGCGAAAACAAC-AGACAACAAAATTTCTTGGCTCCAACTTTTGGCCTT<869      1112<GTTTGACCAGTCAGTCCCTGATGCA-GCAGGACCTTTATGCTAGTCAGAATATCTGTATGAGT-AATAGCACAACCAGTAACATTCTAGATCCTCCACTC<1015              *         *         *         *         *         *         *         *         *         *       5696>gacggtgattccaggaacagcttgctcggtggagctaatgttgataatggctttgtgcctgacacgctactctcgaggggatatgactcccagaaagatc>5795     1013<GACGGTGATTCCAGGAACAGCTTGCTCGGTGGAGCTAATGTTGATAATGGCTTTGTGCCTGACACGCTACTCTCGAGGGGATATGACTCCCAGAAAGATC<914       868<GACGGTGATTCCAGGAACAGCTTGCTCGGTGGAGCTAATGTTGATAATGGCTTTGTGCCTGACACGCTACTCTCGAGGGGATATGACTCCCAGAAAGATC<769      1158<GACGGTGAT-CCAGGAACAGCTTGCTCGGTGGAGCTAATGTTGATAATGGCTTTGTGCCTGACACGCTACTCTCGAGGGGATATGACTCCCAGAAAGATC<1060     1014<CAAACACAGTCCTTGATGACTTCTGTGCCATCAAAGACACTGATTTCCAGAACCACCCTTTCTGGTTGTTTGGTTGGAAACAACAACACTAGCTTTGCTC<915       868<GACGGTGATTCCAGGAACAGCTTGCTCGGTGGAGCTAATGTTGATAATGGCTTTGTGCCTGACACGCTACTCTCGAGGGGATATGACTCCCAGAAAGATC<769      1014<TCAAACACAGTCCTTGATGACTTCTGTGCCATCAAAGACACTGATTTCCAGAACCACCTTTCTGGTTGTTTGGTTGGAAACAACAACACTAGCTTTGCTC<915               *         *         *         *         *         *         *         *         *         *       5796>ttcagaacatgctttcaaactatggaggagtgacaaatgacattggtacagagatgtctacttcagctgtaagaactcaatcttttggtgtccccaatgt>5895      913<TTCAGAACATGCTTTCAAACTATGGAGGAGTGACAAATGACATTGGTACAGAGATGTCTACTTCAGCTGTAAGAACTCAATCTTTTGGTGTCCCCAATGT<814       768<TTCAGAACATGCTTTCAAACTATGGAGGAGTGACAAATGACATTGGTACAGAGATGTCTACTTCAGCTGTAAGAACTCAATCTTTTGGTGTCCCCAATGT<669      1059<TTCAGAACATGCTTTCAAACTATGGAGGAGTGACAAATGACATTGGTACAGAGATGTCTACTTCAGCTGTAAGAACTCAATCTTTTGGTGTCCCCAATGT<960       914<AAGATGTCCAGTCGCAGATCACATCAGCTAGCTTTGCAGACTCACAGGCCTTCTCTCGCCAAGATTTTCCAGATAATTCTGGAGGCACTGGTACATCTTC<815       768<TTCAGAACATGCTTTCAAACTATGGAGGAGTGACAAATGACATTGGTACAGAGATGTCTACTTCAGCTGTAAGAACTCAATCTTTTGGTGTCCCCAATGT<669       914<AAGATGTCCAGTCGCAGATCACATCAGCTAGCTTTGCAGACTCACAGGCCTTCTCTCGCCAAGATTTTCCAGATAATTCTGGAGGCACTGGTACATCTTC<815               *         *         *         *         *         *         *         *         *         *       5896>gcccgccatttcgaacgatctagctgtcaacgatgctggagttcttggtggtggattgtggccagctcagactcagcgaatgcgaactggcagcgatctg>5995      813<GCCCGCCATTTCGAACGATCTAGCTGTCAACGATGCTGGAGTTCTTGGTGGTGGATTGTGGCCAGCTCAGACTCAGCGAATGCGAACTGGCAGCGATCTG<714       668<GCCCGCCATTTCGAACGATCTAGCTGTCAACGATGCTGGAGTTCTTGGTGGTGGATTGTGGCCAGCTCAGACTCAGCGAATGCGAACTGGCAGCGATCTG<569       959<GCCCGCCATTTCGAACGATCTAGCTGTCAACGATGCTGGAGTTCTTGGTGGTGGATTGTGGCCAGCTCAGACTCAGCGAATGCGAACTGGCAGCGATCTG<860       814<AAGCAATGTTGATTTTGATGATTGTAGTCTGCGGCAAAATAGTAAAGGCTCATCATGGCAGAAAATTGCGACACCCCGCGTCCGAACCGGCAGCGATCTG<715       668<GCCCGCCATTTCGAACGATCTAGCTGTCAACGATGCTGGAGTTCTTGGTGGTGGATTGTGGCCAGCTCAGACTCAGCGAATGCGAACTGGCAGCGATCTG<569       814<AAGCAATGTTGATTTTGATGATTGTAGTCTGCGGCAAAATAGTAAAGGCTCATCATGGCAGAAAATTGCGACACCCCGCGTCCGAACCGGCAGCGATCTG<715               *         *         *         *         *         *         *         *         *         *       5996>ggtaaaaagctgctggaagcagccgcggccggccaagatgatgaggtgcgtattctgatggcgaatggggccgatgttaacgcaaccgacgacgatggcc>6095      713<GGTAAAAAGCTGCTGGAAGCAGCCGCGGCCGGCCAAGATGATGAGGTGCGTATTCTGATGGCGAATGGGGCCGATGTTAACGCAACCGACGACGATGGCC<614       568<GGTAAAAAGCTGCTGGAAGCAGCCGCGGCCGGCCAAGATGATGAGGTGCGTATTCTGATGGCGAATGGGGCCGATGTTAACGCAACCGACGACGATGGCC<469       859<GGTAAAAAGCTGCTGGAAGCAGCCGCGGCCGGCCAAGATGATGAGGTGCGTATTCTGATGGCGAATGGGGCCGATGTTAACGCAACCGACGACGATGGCC<760       714<GGTAAAAAGCTGCTGGAAGCAGCCGCGGCCGGCCAAGATGATGAGGTGCGTATTCTGATGGCGAATGGGGCCGATGTTAACGCAACCGACGACAATGGCC<615       568<GGTAAAAAGCTGCTGGAAGCAGCCGCGGCCGGCCAAGATGATGAGGTGCGTATTCTGATGGCGAATGGGGCCGATGTTAACGCAACCGACGACGATGGCC<469       714<GGTAAAAAGCTGCTGGAAGCAGCCGCGGCCGGCCAAGATGATGAGGTGCGTATTCTGATGGCGAATGGGGCCGATGTTAACGCAACCGACGACAATGGCC<615               *         *         *         *         *         *         *                                     6096>tgactccgctgcacctggcggctgcaaacgggcaactggaaatcgtagaggtactgctgaaaaatggcgc------------------------------>6165      613<TGACTCCGCTGCACCTGGCGGCTGCAAACGGGCAACTGGAAATCGTAGAGGTACTGCTGAAAAATGGCGC------------------------------<544       468<TGACTCCGCTGCACCTGGCGGCTGCAAACGGGCAACTGGAAATCGTAGAGGTACTGCTGAAAAATGGCGC------------------------------<399       759<TGACTCCGCTGCACCTGGCGGCTGCAAACGGGCAACTGGAAATCGTAGAGGTACTGCTGAAAAATGGCGCCGATGTTAACGCAACCGACGACGATGGCCT<660       614<TGACTCCGCTGCACCTGGCGGCTGCAAACGGGCAACTGGAAATCGTAGAGGTACTGCTGAAAAATGGCGC------------------------------<545       468<TGACTCCGCTGCACCTGGCGGCTGCAAACGGGCAACTGGAAATCGTAGAGGTACTGCTGAAAAATGGCGC------------------------------<399       614<TGACTCCGCTGCACCTGGCGGCTGCAAACGGGCAACTGGAAATCGTAGAGGTACTGCTGAAAAATGGCGC------------------------------<545                                                                                                                 6165>---------------------------------------------------------------------------------------------------->6165      544<----------------------------------------------------------------------------------------------------<544       399<----------------------------------------------------------------------------------------------------<399       659<GACTCCGCTGCACCTGCCGATGTTAACGCAACCGACGACGATGGCCTGACTCCGCTGCACCTGGCGGCTGCAAACGGGCAACTGGAAATCGTAGAGGTAC<560       545<----------------------------------------------------------------------------------------------------<545       399<----------------------------------------------------------------------------------------------------<399       545<----------------------------------------------------------------------------------------------------<545                               *         *         *         *         *         *         *         *           6166>----------------cgatgtgaacgcttctgatagtgcgggtattactccgctgcacctggccgcttatgacggccatctggagattgtcgaagtcct>6249      543<----------------CGATGTGAACGCTTCTGATAGTGCGGGTATTACTCCGCTGCACCTGGCCGCTTATGACGGCCATCTGGAGATTGTCGAAGTCCT<460       398<----------------CGATGTGAACGCTTCTGATAGTGCGGGTATTACTCCGCTGCACCTGGCCGCTTATGACGGCCATCTGGAGATTGTCGAAGTCCT<315       559<TGCTGAAAAATGGCGCCGATGTGAACGCTTCTGATAGTGCGGGTATTACTCCGCTGCACCTGGCCGCTTATGACGGCCATCTGGAGATTGTCGAAGTCCT<460       544<----------------CGATGTGAACGCTTCTGATAGTGCGGGTATTACTCCGCTGCACCTGGCCGCTTATGACGGCCATCTGGAGATTGTCGAAGTCCT<461       398<----------------CGATGTGAACGCTTCTGATAGTGCGGGTATTACTCCGCTGCACCTGGCCGCTTATGACGGCCATCTGGAGATTGTCGAAGTCCT<315       544<----------------CGATGTGAACGCTTCTGATAGTGCGGGTATTACTCCGCTGCACCTGGCCGCTTATGACGGCCATCTGGAGATTGTCGAAGTCCT<461           *         *         *         *         *         *         *         *         *         *           6250>gctgaagcacggggctgacgttaatgcgtacgaccgcgccgggtggacaccgctgcacctagcagcgctgagtggccaactggagattgtggaagttctg>6349      459<GCTGAAGCACGGGGCTGACGTTAATGCGTACGACCGCGCCGGGTGGACACCGCTGCACCTAGCAGCGCTGAGTGGCCAACTGGAGATTGTGGAAGTTCTG<360       314<GCTGAAGCACGGGGCTGACGTTAATGCGTACGACCGCGCCGGGTGGACACCGCTGCACCTAGCAGCGCTGAGTGGCCAACTGGAGATTGTGGAAGTTCTG<215       459<GCTGAAGCACGGGGCTGACGTTAATGCGTACGACCGCGCCGGGTGGACACCGCTGCACCTAGCAGCGCTGAGTGGCCAACTGGAGATTGTGGAAGTTCTG<360       460<GCTGAAGCACGGGGCTGACGTTAATGCGTACGACCGCGCCGGGTGGACACCGCTGCACCTAGCAGCGCTGAGTGGCCAACTGGAGATTGTGGAAGTTCTG<361       314<GCTGAAGCACGGGGCTGACGTTAATGCGTACGACCGCGCCGGGTGGACACCGCTGCACCTAGCAGCGCTGAGTGGCCAACTGGAGATTGTGGAAGTTCTG<215       460<GCTGAAGCACGGGGCTGACGTTAATGCGTACGACCGCGCCGGGTGGACACCGCTGCACCTAGCAGCGCTGAGTGGCCAACTGGAGATTGTGGAAGTTCTG<361           *         *         *         *         *         *         *         *         *         *           6350>ctgaaacacggcgcagatgtcaacgcccaagacgcactgggcctgaccgcgtttgatatctcgattaatcaaggtcaggaagatctggcagagatcctgc>6449      359<CTGAAACACGGCGCAGATGTCAACGCCCAAGACGCACTGGGCCTGACCGCGTTTGATATCTCGATTAATCAAGGTCAGGAAGATCTGGCAGAGATCCTGC<260       214<CTGAAACACGGCGCAGATGTCAACGCCCAAGACGCACTGGGCCTGACCGCGTTTGATATCTCGATTAATCAAGGTCAGGAAGATCTGGCAGAGATCCTGC<115       359<CTGAAACACGGCGCAGATGTCAACGCCCAAGACGCACTGGGCCTGACCGCGTTTGATATCTCGATTAATCAAGGTCAGGAAGATCTGGCAGAGATCCTGC<260       360<CTGAAACACGGCGCAGATGTCAACGCCCAAGACGCACTGGGCCTGACCGCGTTTGATATCTCGATTAATCAAGGTCAGGAAGATCTGGCAGAGATCCTGC<261       214<CTGAAACACGGCGCAGATGTCAACGCCCAAGACGCACTGGGCCTGACCGCGTTTGATATCTCGATTAATCAAGGTCAGGAAGATCTGGCAGAGATCCTGC<115       360<CTGAAACACGGCGCAGATGTCAACGCCCAAGACGCACTGGGCCTGACCGCGTTTGATATCTCGATTAATCAAGGTCAGGAAGATCTGGCAGAGATCCTGC<261           *         *         *         *                                                                                     H  H  H  H  H  H      ---------------------------------------------------------------------               CACcacCACcacCACcac     ---------------------------------------------------------------------  6450>aactcgagcaccaccaccaccaccactgaca--------------------------------------------------------------------->6480      259<AACTCGAGCACCACCACCACCACCACTGACACCCAGCTTTCTTGTCCACCACCACTGACACCCAGCTTTCTTGTCCACCACCACTGACACCCAGCTTTCT<160       114<AACTCGAGCACCACCACCACCACCACTGACA---------------------------------------------------------------------<84        259<AACTCGAGCACCACCACCACCACCACTGACACCCAGCTTTCTTGTCCACCACCACTGACACCCAGCTTTCTTGTCCACCACCACTGACACCCAGCTTTCT<160       260<AACTCGAGCACCACCACCACCACCACTGACACCCAGCTTTCTTGTCCACCACCACTGACACCCAGCTTTCTTGTCCACCACCACTGACACCCAGCTTTCT<161       114<AACTCGAGCACCACCACCACCACCACTGACA---------------------------------------------------------------------<84        260<AACTCGAGCACCACCACCACCACCACTGACACCCAGCTTTCTTGTCCACCACCACTGACACCCAGCTTTCTTGTCCACCACCACTGACACCCAGCTTTCT<161                                                                                                *         *      6481>----------------------------------------------------------------------------cccagctttcttgtacaaagtggt>6504      159<TGTCCACCACCACTGACACCCAGCTTTCTTGTCCACCACCACTGACACCCAGCTTTCTTGTCCACCACCACTGACACCCAGCTTTCTTGT----------<70         83<----------------------------------------------------------------------------CCCAGCTTTCTTGTAC--------<68        159<TGTCCACCACCACTGACACCCAGCTTTCTTGTCCACCACCACTGACACCCAGCTTTCTTGTCCACCACCACTGACACCCAGCTTTCTTGT----------<70        160<TGTCCACCACCACTGACACCCAGCTTTCTTGTCCACCACCACTGACACCCAGCTTTCTTGTCCACCACCACTGACACCCAGCTTTCTTGT----------<71         83<----------------------------------------------------------------------------CCCAGCTTTCTTGTAC--------<68        160<TGTCCACCACCACTGACACCCAGCTTTCTTGTCCACCACCACTGACACCCAGCTTTCTTGTCCACCACCACTGACACCCAGCTTTCTTGT----------<71                 *         *         *         *         *         *         *         *         *         *      6505>tcttgtacaaagtggtgatgggctgcaggaattcgatatcaagcttatcgataccgtcgacctcgagtcatgtaattagttatgtcacgcttacattcac>6604       69<------ACAAAGTGGTGATGGGCTGCAGGAATTCGAT-TCAAGCTTATCGATACCGTCGACCTCGAGTCA------------------------------<7          67<--------AAAGTGGTGATGGGCTGCAGGAATTCGATTTCAAGCTTATCGATACCGTCGACCTCGAGTCA------------------------------<6          69<------ACAAAGTGGTGATGGGCTGCAGGAATTCGATTTCAAGCTTATCGATACCGTCGACCTCGAGTCA------------------------------<6          70<------ACAAAGTGGTGATGGGCTGCAGGAATTCGATTTCAAGCTTATCGATACCGTCGACCTCGAGTC-------------------------------<8          67<--------AAAGTGGTGATGGGCTGCAGGAATTCGAT-TCAAGCTTATCGATACCGTCGACCTCGAGTCA------------------------------<7          70<------ACAAAGTGGTGATGGGCTGCAGGAATTCGATTTCAAGCTTATCGATACCGTCGACCTCGAGTCA------------------------------<7                  *         *         *         *         *         *         *         *         *         *      6605>gccctccccccacatccgctctaaccgaaaaggaaggagttagacaacctgaagtctaggtccctatttatttttttatagttatgttagtattaagaac>6704        7<----------------------------------------------------------------------------------------------------<7           6<----------------------------------------------------------------------------------------------------<6           6<----------------------------------------------------------------------------------------------------<6           8<----------------------------------------------------------------------------------------------------<8           7<----------------------------------------------------------------------------------------------------<7           7<----------------------------------------------------------------------------------------------------<7                  *         *         *         *         *         *         *         *         *         *      6705>gttatttatatttcaaatttttcttttttttctgtacagacgcgtgtacgcatgtaacattatactgaaaaccttgcttgagaaggttttgggacgctcg>6804        7<----------------------------------------------------------------------------------------------------<7           6<----------------------------------------------------------------------------------------------------<6           6<----------------------------------------------------------------------------------------------------<6           8<----------------------------------------------------------------------------------------------------<8           7<----------------------------------------------------------------------------------------------------<7           7<----------------------------------------------------------------------------------------------------<7                  *         *         *         *         *         *         *         *         *         *      6805>aaggctttaatttgtgacaccgattatttaaagctgcagcatacgatatatatacatgtgtatatatgtatacctatgaatgtcagtaagtatgtatacg>6904        7<----------------------------------------------------------------------------------------------------<7           6<----------------------------------------------------------------------------------------------------<6           6<----------------------------------------------------------------------------------------------------<6           8<----------------------------------------------------------------------------------------------------<8           7<----------------------------------------------------------------------------------------------------<7           7<----------------------------------------------------------------------------------------------------<7                  *         *         *         *         *         *         *         *         *         *      6905>aacagtatgatactgaagatgacaaggtaatgcatcattctatacgtgtcattctgaacgaggcgcgctttccttttttctttttgctttttcttttttt>7004        7<----------------------------------------------------------------------------------------------------<7           5<----GTATG~~~~~~~~~~~~~~~~~~~~~~~~~~~~~~~~~~~~~~~~~~~~~~~~~~~~~~~~~~~~~~~~~~~~~~~~~~~~~~~~~~~~~~~~~~~<1           6<----------------------------------------------------------------------------------------------------<6           8<----------------------------------------------------------------------------------------------------<8           7<----------------------------------------------------------------------------------------------------<7           7<----------------------------------------------------------------------------------------------------<7                  *         *         *         *         *         *         *         *         *         *      7005>ttctcttgaactcgagaaaaaaaatataaaagagatggaggaacgggaaaaagttagttgtggtgataggtggcaagtggtattccgtaagaacaacaag>7104        7<----------------------------------------------------------------------------------------------------<7           1<~~~~~~~~~~~~~~~~~~~~~~~~~~~~~~~~~~~~~~~~~~~~~~~~~~~~~~~~~~~~~~~~~~~~~~~~~~~~~~~~~~~~~~~~~~~~~~~~~~~~<1           5<--------------------------------------------------------------------------------------GTAAG~~~~~~~~~<1           8<----------------------------------------------------------------------------------------------------<8           7<----------------------------------------------------------------------------------------------------<7           6<------------------------------------------------------------------------------GGTATT~~~~~~~~~~~~~~~~<1                  *         *         *         *         *         *         *         *         *         *      7105>aaaagcatttcatattatggctgaactgagcgaacaagtgcaaaatttaagcatcaacgacaacaacgagaatggttatgttcctcctcacttaagagga>7204        7<----------------------------------------------------------------------------------------------------<7           1<~~~~~~~~~~~~~~~~~~~~~~~~~~~~~~~~~~~~~~~~~~~~~~~~~~~~~~~~~~~~~~~~~~~~~~~~~~~~~~~~~~~~~~~~~~~~~~~~~~~~<1           1<~~~~~~~~~~~~~~~~~~~~~~~~~~~~~~~~~~~~~~~~~~~~~~~~~~~~~~~~~~~~~~~~~~~~~~~~~~~~~~~~~~~~~~~~~~~~~~~~~~~~<1           8<----------------------------------------------------------------------------------------------------<8           7<----------------------------------------------------------------------------------------------------<7           1<~~~~~~~~~~~~~~~~~~~~~~~~~~~~~~~~~~~~~~~~~~~~~~~~~~~~~~~~~~~~~~~~~~~~~~~~~~~~~~~~~~~~~~~~~~~~~~~~~~~~<1                  *         *         *         *         *         *         *         *         *         *      7205>aaaccaagaagtgccagaaataacagtagcaactacaataacaacaacggcggctacaacggtggccgtggcggtggcagcttctttagcaacaaccgtc>7304        7<----------------------------------------------------------------------------------------------------<7           1<~~~~~~~~~~~~~~~~~~~~~~~~~~~~~~~~~~~~~~~~~~~~~~~~~~~~~~~~~~~~~~~~~~~~~~~~~~~~~~~~~~~~~~~~~~~~~~~~~~~~<1           1<~~~~~~~~~~~~~~~~~~~~~~~~~~~~~~~~~~~~~~~~~~~~~~~~~~~~~~~~~~~~~~~~~~~~~~~~~~~~~~~~~~~~~~~~~~~~~~~~~~~~<1           8<----------------------------------------------------------------------------------------------------<8           7<----------------------------------------------------------------------------------------------------<7           1<~~~~~~~~~~~~~~~~~~~~~~~~~~~~~~~~~~~~~~~~~~~~~~~~~~~~~~~~~~~~~~~~~~~~~~~~~~~~~~~~~~~~~~~~~~~~~~~~~~~~<1                  *         *         *         *         *         *         *         *         *         *      7305>gtggtggttacggcaacggtggtttcttcggtggaaacaacggtggcagcagatctaacggccgttctggtggtagatggatcgatggcaaacatgtccc>7404        7<----------------------------------------------------------------------------------------------------<7           1<~~~~~~~~~~~~~~~~~~~~~~~~~~~~~~~~~~~~~~~~~~~~~~~~~~~~~~~~~~~~~~~~~~~~~~~~~~~~~~~~~~~~~~~~~~~~~~~~~~~~<1           1<~~~~~~~~~~~~~~~~~~~~~~~~~~~~~~~~~~~~~~~~~~~~~~~~~~~~~~~~~~~~~~~~~~~~~~~~~~~~~~~~~~~~~~~~~~~~~~~~~~~~<1           8<----------------------------------------------------------------------------------------------------<8           7<----------------------------------------------------------------------------------------------------<7           1<~~~~~~~~~~~~~~~~~~~~~~~~~~~~~~~~~~~~~~~~~~~~~~~~~~~~~~~~~~~~~~~~~~~~~~~~~~~~~~~~~~~~~~~~~~~~~~~~~~~~<1                  *         *         *         *         *         *         *         *         *         *      7405>agctccaagaaacgaaaaggccgagatcgccatatttggtgtccccgaggatccaaatttccaatcttctggtattaacttcgataactacgatgatatt>7504        7<----------------------------------------------------------------------------------------------------<7           1<~~~~~~~~~~~~~~~~~~~~~~~~~~~~~~~~~~~~~~~~~~~~~~~~~~~~~~~~~~~~~~~~~~~~~~~~~~~~~~~~~~~~~~~~~~~~~~~~~~~~<1           1<~~~~~~~~~~~~~~~~~~~~~~~~~~~~~~~~~~~~~~~~~~~~~~~~~~~~~~~~~~~~~~~~~~~~~~~~~~~~~~~~~~~~~~~~~~~~~~~~~~~~<1           8<----------------------------------------------------------------------------------------------------<8           7<----------------------------------------------------------------------------------------------------<7           1<~~~~~~~~~~~~~~~~~~~~~~~~~~~~~~~~~~~~~~~~~~~~~~~~~~~~~~~~~~~~~~~~~~~~~~~~~~~~~~~~~~~~~~~~~~~~~~~~~~~~<1                  *         *         *         *         *         *         *         *         *         *      7505>ccagtggacgcctctggtaaggatgttcctgaaccaatcacagaatttacctcacctccattggacggattgttattggaaaacatcaaattggcccgtt>7604        7<----------------------------------------------------------------------------------------------------<7           1<~~~~~~~~~~~~~~~~~~~~~~~~~~~~~~~~~~~~~~~~~~~~~~~~~~~~~~~~~~~~~~~~~~~~~~~~~~~~~~~~~~~~~~~~~~~~~~~~~~~~<1           1<~~~~~~~~~~~~~~~~~~~~~~~~~~~~~~~~~~~~~~~~~~~~~~~~~~~~~~~~~~~~~~~~~~~~~~~~~~~~~~~~~~~~~~~~~~~~~~~~~~~~<1           8<----------------------------------------------------------------------------------------------------<8           7<----------------------------------------------------------------------------------------------------<7           1<~~~~~~~~~~~~~~~~~~~~~~~~~~~~~~~~~~~~~~~~~~~~~~~~~~~~~~~~~~~~~~~~~~~~~~~~~~~~~~~~~~~~~~~~~~~~~~~~~~~~<1                  *         *         *         *         *         *         *         *         *         *      7605>tcaccaagccaacacctgtgcaaaaatactccgtccctatcgttgccaacggcagagatttgatggcctgtgcgcagaccggttctggtaagactggtgg>7704        7<----------------------------------------------------------------------------------------------------<7           1<~~~~~~~~~~~~~~~~~~~~~~~~~~~~~~~~~~~~~~~~~~~~~~~~~~~~~~~~~~~~~~~~~~~~~~~~~~~~~~~~~~~~~~~~~~~~~~~~~~~~<1           1<~~~~~~~~~~~~~~~~~~~~~~~~~~~~~~~~~~~~~~~~~~~~~~~~~~~~~~~~~~~~~~~~~~~~~~~~~~~~~~~~~~~~~~~~~~~~~~~~~~~~<1           8<----------------------------------------------------------------------------------------------------<8           7<----------------------------------------------------------------------------------------------------<7           1<~~~~~~~~~~~~~~~~~~~~~~~~~~~~~~~~~~~~~~~~~~~~~~~~~~~~~~~~~~~~~~~~~~~~~~~~~~~~~~~~~~~~~~~~~~~~~~~~~~~~<1                  *         *         *         *         *         *         *         *         *         *      7705>gtttttattcccagtgttgtccgaatcatttaagactggaccatctcctcaaccagagtctcaaggctccttttaccaaagaaaggcctacccaactgct>7804        7<----------------------------------------------------------------------------------------------------<7           1<~~~~~~~~~~~~~~~~~~~~~~~~~~~~~~~~~~~~~~~~~~~~~~~~~~~~~~~~~~~~~~~~~~~~~~~~~~~~~~~~~~~~~~~~~~~~~~~~~~~~<1           1<~~~~~~~~~~~~~~~~~~~~~~~~~~~~~~~~~~~~~~~~~~~~~~~~~~~~~~~~~~~~~~~~~~~~~~~~~~~~~~~~~~~~~~~~~~~~~~~~~~~~<1           8<----------------------------------------------------------------------------------------------------<8           7<----------------------------------------------------------------------------------------------------<7           1<~~~~~~~~~~~~~~~~~~~~~~~~~~~~~~~~~~~~~~~~~~~~~~~~~~~~~~~~~~~~~~~~~~~~~~~~~~~~~~~~~~~~~~~~~~~~~~~~~~~~<1                  *         *         *         *         *         *         *         *         *         *      7805>gtcattatggctccagtttaaaccatggtcatagctgtttcctgtgtgaaattgttatccgctcacaattccacacaacataggagccggaagcataaag>7904        7<----------------------------------------------------------------------------------------------------<7           1<~~~~~~~~~~~~~~~~~~~~~~~~~~~~~~~~~~~~~~~~~~~~~~~~~~~~~~~~~~~~~~~~~~~~~~~~~~~~~~~~~~~~~~~~~~~~~~~~~~~~<1           1<~~~~~~~~~~~~~~~~~~~~~~~~~~~~~~~~~~~~~~~~~~~~~~~~~~~~~~~~~~~~~~~~~~~~~~~~~~~~~~~~~~~~~~~~~~~~~~~~~~~~<1           8<----------------------------------------------------------------------------------------------------<8           7<----------------------------------------------------------------------------------------------------<7           1<~~~~~~~~~~~~~~~~~~~~~~~~~~~~~~~~~~~~~~~~~~~~~~~~~~~~~~~~~~~~~~~~~~~~~~~~~~~~~~~~~~~~~~~~~~~~~~~~~~~~<1                  *         *         *         *         *         *         *         *         *         *      7905>tgtaaagcctggggtgcctaatgagtgaggtaactcacattaattgcgttgcgctcactgcccgctttccagtcgggaaacctgtcgtgccagctgcatt>8004        6<-GTAAAG~~~~~~~~~~~~~~~~~~~~~~~~~~~~~~~~~~~~~~~~~~~~~~~~~~~~~~~~~~~~~~~~~~~~~~~~~~~~~~~~~~~~~~~~~~~~~<1           1<~~~~~~~~~~~~~~~~~~~~~~~~~~~~~~~~~~~~~~~~~~~~~~~~~~~~~~~~~~~~~~~~~~~~~~~~~~~~~~~~~~~~~~~~~~~~~~~~~~~~<1           1<~~~~~~~~~~~~~~~~~~~~~~~~~~~~~~~~~~~~~~~~~~~~~~~~~~~~~~~~~~~~~~~~~~~~~~~~~~~~~~~~~~~~~~~~~~~~~~~~~~~~<1           8<----------------------------------------------------------------------------------------------------<8           7<----------------------------------------------------------------------------------------------------<7           1<~~~~~~~~~~~~~~~~~~~~~~~~~~~~~~~~~~~~~~~~~~~~~~~~~~~~~~~~~~~~~~~~~~~~~~~~~~~~~~~~~~~~~~~~~~~~~~~~~~~~<1                  *         *         *         *         *         *         *         *         *         *      8005>aatgaatcggccaacgcgcggggagaggcggtttgcgtattgggcgctcttccgcttcctcgctcactgactcgctgcgctcggtcgttcggctgcggcg>8104        1<~~~~~~~~~~~~~~~~~~~~~~~~~~~~~~~~~~~~~~~~~~~~~~~~~~~~~~~~~~~~~~~~~~~~~~~~~~~~~~~~~~~~~~~~~~~~~~~~~~~~<1           1<~~~~~~~~~~~~~~~~~~~~~~~~~~~~~~~~~~~~~~~~~~~~~~~~~~~~~~~~~~~~~~~~~~~~~~~~~~~~~~~~~~~~~~~~~~~~~~~~~~~~<1           1<~~~~~~~~~~~~~~~~~~~~~~~~~~~~~~~~~~~~~~~~~~~~~~~~~~~~~~~~~~~~~~~~~~~~~~~~~~~~~~~~~~~~~~~~~~~~~~~~~~~~<1           8<----------------------------------------------------------------------------------------------------<8           7<----------------------------------------------------------------------------------------------------<7           1<~~~~~~~~~~~~~~~~~~~~~~~~~~~~~~~~~~~~~~~~~~~~~~~~~~~~~~~~~~~~~~~~~~~~~~~~~~~~~~~~~~~~~~~~~~~~~~~~~~~~<1                  *         *         *         *         *         *         *         *         *         *      8105>agcggtatcagctcactcaaaggcggtaatacggttatccacagaatcaggggataacgcaggaaagaacatgtgagcaaaaggccagcaaaaggccagg>8204        1<~~~~~~~~~~~~~~~~~~~~~~~~~~~~~~~~~~~~~~~~~~~~~~~~~~~~~~~~~~~~~~~~~~~~~~~~~~~~~~~~~~~~~~~~~~~~~~~~~~~~<1           1<~~~~~~~~~~~~~~~~~~~~~~~~~~~~~~~~~~~~~~~~~~~~~~~~~~~~~~~~~~~~~~~~~~~~~~~~~~~~~~~~~~~~~~~~~~~~~~~~~~~~<1           1<~~~~~~~~~~~~~~~~~~~~~~~~~~~~~~~~~~~~~~~~~~~~~~~~~~~~~~~~~~~~~~~~~~~~~~~~~~~~~~~~~~~~~~~~~~~~~~~~~~~~<1           8<----------------------------------------------------------------------------------------------------<8           7<----------------------------------------------------------------------------------------------------<7           1<~~~~~~~~~~~~~~~~~~~~~~~~~~~~~~~~~~~~~~~~~~~~~~~~~~~~~~~~~~~~~~~~~~~~~~~~~~~~~~~~~~~~~~~~~~~~~~~~~~~~<1                  *         *         *         *         *         *         *         *         *         *      8205>aaccgtaaaaaggccgcgttgctggcgtttttccataggctcggcccccctgacgagcatcacaaaaatcgacgctcaagtcagaggtggcgaaacccga>8304        1<~~~~~~~~~~~~~~~~~~~~~~~~~~~~~~~~~~~~~~~~~~~~~~~~~~~~~~~~~~~~~~~~~~~~~~~~~~~~~~~~~~~~~~~~~~~~~~~~~~~~<1           1<~~~~~~~~~~~~~~~~~~~~~~~~~~~~~~~~~~~~~~~~~~~~~~~~~~~~~~~~~~~~~~~~~~~~~~~~~~~~~~~~~~~~~~~~~~~~~~~~~~~~<1           1<~~~~~~~~~~~~~~~~~~~~~~~~~~~~~~~~~~~~~~~~~~~~~~~~~~~~~~~~~~~~~~~~~~~~~~~~~~~~~~~~~~~~~~~~~~~~~~~~~~~~<1           8<----------------------------------------------------------------------------------------------------<8           7<----------------------------------------------------------------------------------------------------<7           1<~~~~~~~~~~~~~~~~~~~~~~~~~~~~~~~~~~~~~~~~~~~~~~~~~~~~~~~~~~~~~~~~~~~~~~~~~~~~~~~~~~~~~~~~~~~~~~~~~~~~<1                  *         *         *         *         *         *         *         *         *         *      8305>caggactataaagataccaggcgttcccccctggaagctccctcgtgcgctctcctgttccgaccctgccgcttaccggatacctgtccgcctttctccc>8404        1<~~~~~~~~~~~~~~~~~~~~~~~~~~~~~~~~~~~~~~~~~~~~~~~~~~~~~~~~~~~~~~~~~~~~~~~~~~~~~~~~~~~~~~~~~~~~~~~~~~~~<1           1<~~~~~~~~~~~~~~~~~~~~~~~~~~~~~~~~~~~~~~~~~~~~~~~~~~~~~~~~~~~~~~~~~~~~~~~~~~~~~~~~~~~~~~~~~~~~~~~~~~~~<1           1<~~~~~~~~~~~~~~~~~~~~~~~~~~~~~~~~~~~~~~~~~~~~~~~~~~~~~~~~~~~~~~~~~~~~~~~~~~~~~~~~~~~~~~~~~~~~~~~~~~~~<1           8<----------------------------------------------------------------------------------------------------<8           7<----------------------------------------------------------------------------------------------------<7           1<~~~~~~~~~~~~~~~~~~~~~~~~~~~~~~~~~~~~~~~~~~~~~~~~~~~~~~~~~~~~~~~~~~~~~~~~~~~~~~~~~~~~~~~~~~~~~~~~~~~~<1                  *         *         *         *         *         *         *         *         *         *      8405>ttcgggaagcgtggcgctttctcaatgctcacgctgtaggtatctcagttcggtgtaggtcgttcgctccaagctgggctgtgtgcacgaaccccccgtt>8504        1<~~~~~~~~~~~~~~~~~~~~~~~~~~~~~~~~~~~~~~~~~~~~~~~~~~~~~~~~~~~~~~~~~~~~~~~~~~~~~~~~~~~~~~~~~~~~~~~~~~~~<1           1<~~~~~~~~~~~~~~~~~~~~~~~~~~~~~~~~~~~~~~~~~~~~~~~~~~~~~~~~~~~~~~~~~~~~~~~~~~~~~~~~~~~~~~~~~~~~~~~~~~~~<1           1<~~~~~~~~~~~~~~~~~~~~~~~~~~~~~~~~~~~~~~~~~~~~~~~~~~~~~~~~~~~~~~~~~~~~~~~~~~~~~~~~~~~~~~~~~~~~~~~~~~~~<1           8<----------------------------------------------------------------------------------------------------<8           7<----------------------------------------------------------------------------------------------------<7           1<~~~~~~~~~~~~~~~~~~~~~~~~~~~~~~~~~~~~~~~~~~~~~~~~~~~~~~~~~~~~~~~~~~~~~~~~~~~~~~~~~~~~~~~~~~~~~~~~~~~~<1                  *         *         *         *         *         *         *         *         *         *      8505>cagcccgaccgctgcgccttatccggtaactatcgtcttgagtccaacccggtaagacacgacttatcgccactggcagcagccactggtaacaggatta>8604        1<~~~~~~~~~~~~~~~~~~~~~~~~~~~~~~~~~~~~~~~~~~~~~~~~~~~~~~~~~~~~~~~~~~~~~~~~~~~~~~~~~~~~~~~~~~~~~~~~~~~~<1           1<~~~~~~~~~~~~~~~~~~~~~~~~~~~~~~~~~~~~~~~~~~~~~~~~~~~~~~~~~~~~~~~~~~~~~~~~~~~~~~~~~~~~~~~~~~~~~~~~~~~~<1           1<~~~~~~~~~~~~~~~~~~~~~~~~~~~~~~~~~~~~~~~~~~~~~~~~~~~~~~~~~~~~~~~~~~~~~~~~~~~~~~~~~~~~~~~~~~~~~~~~~~~~<1           8<----------------------------------------------------------------------------------------------------<8           7<----------------------------------------------------------------------------------------------------<7           1<~~~~~~~~~~~~~~~~~~~~~~~~~~~~~~~~~~~~~~~~~~~~~~~~~~~~~~~~~~~~~~~~~~~~~~~~~~~~~~~~~~~~~~~~~~~~~~~~~~~~<1                  *         *         *         *         *         *         *         *         *         *      8605>gcagagcgaggtatgtaggcggtgctacagagttcttgaagtggtggcctaactacggctacactagaaggacagtatttggtatctgcgctctgctgaa>8704        1<~~~~~~~~~~~~~~~~~~~~~~~~~~~~~~~~~~~~~~~~~~~~~~~~~~~~~~~~~~~~~~~~~~~~~~~~~~~~~~~~~~~~~~~~~~~~~~~~~~~~<1           1<~~~~~~~~~~~~~~~~~~~~~~~~~~~~~~~~~~~~~~~~~~~~~~~~~~~~~~~~~~~~~~~~~~~~~~~~~~~~~~~~~~~~~~~~~~~~~~~~~~~~<1           1<~~~~~~~~~~~~~~~~~~~~~~~~~~~~~~~~~~~~~~~~~~~~~~~~~~~~~~~~~~~~~~~~~~~~~~~~~~~~~~~~~~~~~~~~~~~~~~~~~~~~<1           8<----------------------------------------------------------------------------------------------------<8           7<----------------------------------------------------------------------------------------------------<7           1<~~~~~~~~~~~~~~~~~~~~~~~~~~~~~~~~~~~~~~~~~~~~~~~~~~~~~~~~~~~~~~~~~~~~~~~~~~~~~~~~~~~~~~~~~~~~~~~~~~~~<1                  *         *         *         *         *         *         *         *         *         *      8705>gccagttaccttcggaaaaagagttggtagctcttgatccggcaaacaaaccaccgctggtagcggtggtttttttgtttgcaagcagcagattacgcgc>8804        1<~~~~~~~~~~~~~~~~~~~~~~~~~~~~~~~~~~~~~~~~~~~~~~~~~~~~~~~~~~~~~~~~~~~~~~~~~~~~~~~~~~~~~~~~~~~~~~~~~~~~<1           1<~~~~~~~~~~~~~~~~~~~~~~~~~~~~~~~~~~~~~~~~~~~~~~~~~~~~~~~~~~~~~~~~~~~~~~~~~~~~~~~~~~~~~~~~~~~~~~~~~~~~<1           1<~~~~~~~~~~~~~~~~~~~~~~~~~~~~~~~~~~~~~~~~~~~~~~~~~~~~~~~~~~~~~~~~~~~~~~~~~~~~~~~~~~~~~~~~~~~~~~~~~~~~<1           8<----------------------------------------------------------------------------------------------------<8           7<----------------------------------------------------------------------------------------------------<7           1<~~~~~~~~~~~~~~~~~~~~~~~~~~~~~~~~~~~~~~~~~~~~~~~~~~~~~~~~~~~~~~~~~~~~~~~~~~~~~~~~~~~~~~~~~~~~~~~~~~~~<1                  *         *         *         *         *         *         *         *         *         *      8805>agaaaaaaaggatctcaagaagatcctttgatcttttctacggggtctgacgctcagtggaacgaaaactcacgttaagggattttggtcatgagattat>8904        1<~~~~~~~~~~~~~~~~~~~~~~~~~~~~~~~~~~~~~~~~~~~~~~~~~~~~~~~~~~~~~~~~~~~~~~~~~~~~~~~~~~~~~~~~~~~~~~~~~~~~<1           1<~~~~~~~~~~~~~~~~~~~~~~~~~~~~~~~~~~~~~~~~~~~~~~~~~~~~~~~~~~~~~~~~~~~~~~~~~~~~~~~~~~~~~~~~~~~~~~~~~~~~<1           1<~~~~~~~~~~~~~~~~~~~~~~~~~~~~~~~~~~~~~~~~~~~~~~~~~~~~~~~~~~~~~~~~~~~~~~~~~~~~~~~~~~~~~~~~~~~~~~~~~~~~<1           8<----------------------------------------------------------------------------------------------------<8           7<----------------------------------------------------------------------------------------------------<7           1<~~~~~~~~~~~~~~~~~~~~~~~~~~~~~~~~~~~~~~~~~~~~~~~~~~~~~~~~~~~~~~~~~~~~~~~~~~~~~~~~~~~~~~~~~~~~~~~~~~~~<1                  *         *         *         *         *         *         *         *         *         *                                                                                                    *  W  H  K                                                                                                TTAccaATGctt  8905>caaaaaggatcttcacctagatccttttaaattaaaaatgaagttttaaatcaatctaaagtatatatgagtaaacttggtctgacagttaccaatgctt>9004        1<~~~~~~~~~~~~~~~~~~~~~~~~~~~~~~~~~~~~~~~~~~~~~~~~~~~~~~~~~~~~~~~~~~~~~~~~~~~~~~~~~~~~~~~~~~~~~~~~~~~~<1           1<~~~~~~~~~~~~~~~~~~~~~~~~~~~~~~~~~~~~~~~~~~~~~~~~~~~~~~~~~~~~~~~~~~~~~~~~~~~~~~~~~~~~~~~~~~~~~~~~~~~~<1           1<~~~~~~~~~~~~~~~~~~~~~~~~~~~~~~~~~~~~~~~~~~~~~~~~~~~~~~~~~~~~~~~~~~~~~~~~~~~~~~~~~~~~~~~~~~~~~~~~~~~~<1           8<----------------------------------------------------------------------------------------------------<8           7<----------------------------------------------------------------------------------------------------<7           1<~~~~~~~~~~~~~~~~~~~~~~~~~~~~~~~~~~~~~~~~~~~~~~~~~~~~~~~~~~~~~~~~~~~~~~~~~~~~~~~~~~~~~~~~~~~~~~~~~~~~<1                  *         *         *         *         *         *         *         *         *         *            I  L  S  A  G  I  E  A  I  Q  R  N  R  E  D  M  T  A  Q  S  G  T  T  Y  I  V  V  I  R  S  P  K  G         AATcagTGAggcACCtatCTCagcGATctgTCTattTCGttcATCcatAGTtgcCTGactGCCcgtCGTgtaGATaacTACgatACGggaGGGcttACCa  9005>aatcagtgaggcacctatctcagcgatctgtctatttcgttcatccatagttgcctgactgcccgtcgtgtagataactacgatacgggagggcttacca>9104        1<~~~~~~~~~~~~~~~~~~~~~~~~~~~~~~~~~~~~~~~~~~~~~~~~~~~~~~~~~~~~~~~~~~~~~~~~~~~~~~~~~~~~~~~~~~~~~~~~~~~~<1           1<~~~~~~~~~~~~~~~~~~~~~~~~~~~~~~~~~~~~~~~~~~~~~~~~~~~~~~~~~~~~~~~~~~~~~~~~~~~~~~~~~~~~~~~~~~~~~~~~~~~~<1           1<~~~~~~~~~~~~~~~~~~~~~~~~~~~~~~~~~~~~~~~~~~~~~~~~~~~~~~~~~~~~~~~~~~~~~~~~~~~~~~~~~~~~~~~~~~~~~~~~~~~~<1           8<----------------------------------------------------------------------------------------------------<8           7<----------------------------------------------------------------------------------------------------<7           1<~~~~~~~~~~~~~~~~~~~~~~~~~~~~~~~~~~~~~~~~~~~~~~~~~~~~~~~~~~~~~~~~~~~~~~~~~~~~~~~~~~~~~~~~~~~~~~~~~~~~<1                  *         *         *         *         *         *         *         *         *         *           D  P  G  L  A  A  I  I  G  R  S  G  R  E  G  A  G  S  K  D  A  I  F  W  G  A  P  L  A  S  R  L  L  P       tcTGGcccCAGtgcTGCaatGATaccGCGagaCCCacgCTCaccGGCtccAGAtttATCagcAATaaaCCAgccAGCcggAAGggcCGAgcgCAGaagTG  9105>tctggccccagtgctgcaatgataccgcgagacccacgctcaccggctccagatttatcagcaataaaccagccagccggaagggccgagcgcagaagtg>9204        1<~~~~~~~~~~~~~~~~~~~~~~~~~~~~~~~~~~~~~~~~~~~~~~~~~~~~~~~~~~~~~~~~~~~~~~~~~~~~~~~~~~~~~~~~~~~~~~~~~~~~<1           1<~~~~~~~~~~~~~~~~~~~~~~~~~~~~~~~~~~~~~~~~~~~~~~~~~~~~~~~~~~~~~~~~~~~~~~~~~~~~~~~~~~~~~~~~~~~~~~~~~~~~<1           1<~~~~~~~~~~~~~~~~~~~~~~~~~~~~~~~~~~~~~~~~~~~~~~~~~~~~~~~~~~~~~~~~~~~~~~~~~~~~~~~~~~~~~~~~~~~~~~~~~~~~<1           8<----------------------------------------------------------------------------------------------------<8           7<----------------------------------------------------------------------------------------------------<7           1<~~~~~~~~~~~~~~~~~~~~~~~~~~~~~~~~~~~~~~~~~~~~~~~~~~~~~~~~~~~~~~~~~~~~~~~~~~~~~~~~~~~~~~~~~~~~~~~~~~~~<1                  *         *         *         *         *         *         *         *         *         *             G  A  V  K  D  A  E  M  W  D  I  L  Q  Q  R  S  A  L  T  L  L  E  G  T  L  L  K  R  L  T  T  A  M        GtccTGCaacTTTatcCGCctcCATccaGTCtatTAAttgTTGccgGGAagcTAGagtAAGtagTTCgccAGTtaaTAGtttGCGcaaCGTtgtTGCcat  9205>gtcctgcaactttatccgcctccatccagtctattaattgttgccgggaagctagagtaagtagttcgccagttaatagtttgcgcaacgttgttgccat>9304        1<~~~~~~~~~~~~~~~~~~~~~~~~~~~~~~~~~~~~~~~~~~~~~~~~~~~~~~~~~~~~~~~~~~~~~~~~~~~~~~~~~~~~~~~~~~~~~~~~~~~~<1           1<~~~~~~~~~~~~~~~~~~~~~~~~~~~~~~~~~~~~~~~~~~~~~~~~~~~~~~~~~~~~~~~~~~~~~~~~~~~~~~~~~~~~~~~~~~~~~~~~~~~~<1           1<~~~~~~~~~~~~~~~~~~~~~~~~~~~~~~~~~~~~~~~~~~~~~~~~~~~~~~~~~~~~~~~~~~~~~~~~~~~~~~~~~~~~~~~~~~~~~~~~~~~~<1           8<----------------------------------------------------------------------------------------------------<8           7<----------------------------------------------------------------------------------------------------<7           1<~~~~~~~~~~~~~~~~~~~~~~~~~~~~~~~~~~~~~~~~~~~~~~~~~~~~~~~~~~~~~~~~~~~~~~~~~~~~~~~~~~~~~~~~~~~~~~~~~~~~<1                  *         *         *         *         *         *         *         *         *         *            A  V  P  M  T  T  D  R  E  D  N  P  I  A  E  N  L  E  P  E  W  R  D  L  R  T  V  H  D  G  M  N  H         TGCtacAGGcatCGTggtGTCacgCTCgtcGTTtggTATggcTTCattCAGctcCGGttcCCAacgATCaagGCGagtTACatgATCcccCATgttGTGa  9305>tgctacaggcatcgtggtgtcacgctcgtcgtttggtatggcttcattcagctccggttcccaacgatcaaggcgagttacatgatcccccatgttgtga>9404        1<~~~~~~~~~~~~~~~~~~~~~~~~~~~~~~~~~~~~~~~~~~~~~~~~~~~~~~~~~~~~~~~~~~~~~~~~~~~~~~~~~~~~~~~~~~~~~~~~~~~~<1           1<~~~~~~~~~~~~~~~~~~~~~~~~~~~~~~~~~~~~~~~~~~~~~~~~~~~~~~~~~~~~~~~~~~~~~~~~~~~~~~~~~~~~~~~~~~~~~~~~~~~~<1           1<~~~~~~~~~~~~~~~~~~~~~~~~~~~~~~~~~~~~~~~~~~~~~~~~~~~~~~~~~~~~~~~~~~~~~~~~~~~~~~~~~~~~~~~~~~~~~~~~~~~~<1           8<----------------------------------------------------------------------------------------------------<8           7<----------------------------------------------------------------------------------------------------<7           1<~~~~~~~~~~~~~~~~~~~~~~~~~~~~~~~~~~~~~~~~~~~~~~~~~~~~~~~~~~~~~~~~~~~~~~~~~~~~~~~~~~~~~~~~~~~~~~~~~~~~<1                  *         *         *         *         *         *         *         *         *         *           F  F  A  T  L  E  K  P  G  G  I  T  T  L  L  L  N  A  A  T  N  D  S  M  T  I  A  A  S  C  L  E  R  V       aaAAAagcGGTtagCTCcttCGGtccTCCgatCGTtgtCAGaagTAAgttGGCcgcAGTgttATCactCATggtTATggcAGCactGCAtaaTTCtctTA  9405>aaaaaagcggttagctccttcggtcctccgatcgttgtcagaagtaagttggccgcagtgttatcactcatggttatggcagcactgcataattctctta>9504        1<~~~~~~~~~~~~~~~~~~~~~~~~~~~~~~~~~~~~~~~~~~~~~~~~~~~~~~~~~~~~~~~~~~~~~~~~~~~~~~~~~~~~~~~~~~~~~~~~~~~~<1           1<~~~~~~~~~~~~~~~~~~~~~~~~~~~~~~~~~~~~~~~~~~~~~~~~~~~~~~~~~~~~~~~~~~~~~~~~~~~~~~~~~~~~~~~~~~~~~~~~~~~~<1           1<~~~~~~~~~~~~~~~~~~~~~~~~~~~~~~~~~~~~~~~~~~~~~~~~~~~~~~~~~~~~~~~~~~~~~~~~~~~~~~~~~~~~~~~~~~~~~~~~~~~~<1           8<----------------------------------------------------------------------------------------------------<8           7<----------------------------------------------------------------------------------------------------<7           1<~~~~~~~~~~~~~~~~~~~~~~~~~~~~~~~~~~~~~~~~~~~~~~~~~~~~~~~~~~~~~~~~~~~~~~~~~~~~~~~~~~~~~~~~~~~~~~~~~~~~<1                  *         *         *         *         *         *         *         *         *         *             T  M  G  D  T  L  H  K  E  T  V  P  S  Y  E  V  L  D  N  Q  S  Y  H  I  R  R  G  L  Q  E  Q  G  A        CtgtCATgccATCcgtAAGatgCTTttcTGTgacTGGtgaGTActcAACcaaGTCattCTGagaATAgtgTATgcgGCGaccGAGttgCTCttgCCCggc  9505>ctgtcatgccatccgtaagatgcttttctgtgactggtgagtactcaaccaagtcattctgagaatagtgtatgcggcgaccgagttgctcttgcccggc>9604        1<~~~~~~~~~~~~~~~~~~~~~~~~~~~~~~~~~~~~~~~~~~~~~~~~~~~~~~~~~~~~~~~~~~~~~~~~~~~~~~~~~~~~~~~~~~~~~~~~~~~~<1           1<~~~~~~~~~~~~~~~~~~~~~~~~~~~~~~~~~~~~~~~~~~~~~~~~~~~~~~~~~~~~~~~~~~~~~~~~~~~~~~~~~~~~~~~~~~~~~~~~~~~~<1           1<~~~~~~~~~~~~~~~~~~~~~~~~~~~~~~~~~~~~~~~~~~~~~~~~~~~~~~~~~~~~~~~~~~~~~~~~~~~~~~~~~~~~~~~~~~~~~~~~~~~~<1           8<----------------------------------------------------------------------------------------------------<8           7<----------------------------------------------------------------------------------------------------<7           1<~~~~~~~~~~~~~~~~~~~~~~~~~~~~~~~~~~~~~~~~~~~~~~~~~~~~~~~~~~~~~~~~~~~~~~~~~~~~~~~~~~~~~~~~~~~~~~~~~~~~<1                  *         *         *         *         *         *         *         *         *         *            D  I  R  S  L  V  A  G  C  L  L  V  K  F  T  S  M  M  P  F  R  E  E  P  R  F  S  E  L  I  K  G  S         GTCaatACGggaTAAtacCGCgccACAtagCAGaacTTTaaaAGTgctCATcatTGGaaaACGttcTTCgggGCGaaaACTctcAAGgatCTTaccGCTg  9605>gtcaatacgggataataccgcgccacatagcagaactttaaaagtgctcatcattggaaaacgttcttcggggcgaaaactctcaaggatcttaccgctg>9704        1<~~~~~~~~~~~~~~~~~~~~~~~~~~~~~~~~~~~~~~~~~~~~~~~~~~~~~~~~~~~~~~~~~~~~~~~~~~~~~~~~~~~~~~~~~~~~~~~~~~~~<1           1<~~~~~~~~~~~~~~~~~~~~~~~~~~~~~~~~~~~~~~~~~~~~~~~~~~~~~~~~~~~~~~~~~~~~~~~~~~~~~~~~~~~~~~~~~~~~~~~~~~~~<1           1<~~~~~~~~~~~~~~~~~~~~~~~~~~~~~~~~~~~~~~~~~~~~~~~~~~~~~~~~~~~~~~~~~~~~~~~~~~~~~~~~~~~~~~~~~~~~~~~~~~~~<1           8<----------------------------------------------------------------------------------------------------<8           7<----------------------------------------------------------------------------------------------------<7           1<~~~~~~~~~~~~~~~~~~~~~~~~~~~~~~~~~~~~~~~~~~~~~~~~~~~~~~~~~~~~~~~~~~~~~~~~~~~~~~~~~~~~~~~~~~~~~~~~~~~~<1                  *         *         *         *         *         *         *         *         *         *           N  L  D  L  E  I  Y  G  V  R  A  G  L  Q  D  E  A  D  K  V  K  V  L  T  E  P  H  A  F  V  P  L  C  F       ttGAGatcCAGttcGATgtaACCcacTCGtgcACCcaaCTGatcTTCagcATCtttTACtttCACcagCGTttcTGGgtgAGCaaaAACaggAAGgcaAA  9705>ttgagatccagttcgatgtaacccactcgtgcacccaactgatcttcagcatcttttactttcaccagcgtttctgggtgagcaaaaacaggaaggcaaa>9804        1<~~~~~~~~~~~~~~~~~~~~~~~~~~~~~~~~~~~~~~~~~~~~~~~~~~~~~~~~~~~~~~~~~~~~~~~~~~~~~~~~~~~~~~~~~~~~~~~~~~~~<1           1<~~~~~~~~~~~~~~~~~~~~~~~~~~~~~~~~~~~~~~~~~~~~~~~~~~~~~~~~~~~~~~~~~~~~~~~~~~~~~~~~~~~~~~~~~~~~~~~~~~~~<1           1<~~~~~~~~~~~~~~~~~~~~~~~~~~~~~~~~~~~~~~~~~~~~~~~~~~~~~~~~~~~~~~~~~~~~~~~~~~~~~~~~~~~~~~~~~~~~~~~~~~~~<1           8<----------------------------------------------------------------------------------------------------<8           7<----------------------------------------------------------------------------------------------------<7           1<~~~~~~~~~~~~~~~~~~~~~~~~~~~~~~~~~~~~~~~~~~~~~~~~~~~~~~~~~~~~~~~~~~~~~~~~~~~~~~~~~~~~~~~~~~~~~~~~~~~~<1                  *         *         *         *         *         *         *         *         *         *             A  A  F  F  P  I  L  A  V  R  F  H  Q  I  S  M                                                           AtgcCGCaaaAAAgggAATaagGGCgacACGgaaATGttgAATactCAT                                                     9805>atgccgcaaaaaagggaataagggcgacacggaaatgttgaatactcatactcttcctttttcaatattattgaagcatttatcagggttattgtctcat>9904        1<~~~~~~~~~~~~~~~~~~~~~~~~~~~~~~~~~~~~~~~~~~~~~~~~~~~~~~~~~~~~~~~~~~~~~~~~~~~~~~~~~~~~~~~~~~~~~~~~~~~~<1           1<~~~~~~~~~~~~~~~~~~~~~~~~~~~~~~~~~~~~~~~~~~~~~~~~~~~~~~~~~~~~~~~~~~~~~~~~~~~~~~~~~~~~~~~~~~~~~~~~~~~~<1           1<~~~~~~~~~~~~~~~~~~~~~~~~~~~~~~~~~~~~~~~~~~~~~~~~~~~~~~~~~~~~~~~~~~~~~~~~~~~~~~~~~~~~~~~~~~~~~~~~~~~~<1           8<----------------------------------------------------------------------------------------------------<8           7<----------------------------------------------------------------------------------------------------<7           1<~~~~~~~~~~~~~~~~~~~~~~~~~~~~~~~~~~~~~~~~~~~~~~~~~~~~~~~~~~~~~~~~~~~~~~~~~~~~~~~~~~~~~~~~~~~~~~~~~~~~<1                  *         *         *         *         *         *         *         *         *         *      9905>gagcggatacatatttgaatgtatttagaaaaataaacaaataggggttccgcgcacatttccccgaaaagtgccacctgacgtcttattatcatgacat>10004       1<~~~~~~~~~~~~~~~~~~~~~~~~~~~~~~~~~~~~~~~~~~~~~~~~~~~~~~~~~~~~~~~~~~~~~~~~~~~~~~~~~~~~~~~~~~~~~~~~~~~~<1           1<~~~~~~~~~~~~~~~~~~~~~~~~~~~~~~~~~~~~~~~~~~~~~~~~~~~~~~~~~~~~~~~~~~~~~~~~~~~~~~~~~~~~~~~~~~~~~~~~~~~~<1           1<~~~~~~~~~~~~~~~~~~~~~~~~~~~~~~~~~~~~~~~~~~~~~~~~~~~~~~~~~~~~~~~~~~~~~~~~~~~~~~~~~~~~~~~~~~~~~~~~~~~~<1           7<-------------------------------------C----AGGGGT~~~~~~~~~~~~~~~~~~~~~~~~~~~~~~~~~~~~~~~~~~~~~~~~~~~~<1           7<----------------------------------------------------------------------------------------------------<7           1<~~~~~~~~~~~~~~~~~~~~~~~~~~~~~~~~~~~~~~~~~~~~~~~~~~~~~~~~~~~~~~~~~~~~~~~~~~~~~~~~~~~~~~~~~~~~~~~~~~~~<1                  *         *         *         * 10005>taacctataaaaataggcgtatcacgaggccctttcgtc>10043       1<~~~~~~~~~~~~~~~~~~~~~~~~~~~~~~~~~~~~~~~<1           1<~~~~~~~~~~~~~~~~~~~~~~~~~~~~~~~~~~~~~~~<1           1<~~~~~~~~~~~~~~~~~~~~~~~~~~~~~~~~~~~~~~~<1           1<~~~~~~~~~~~~~~~~~~~~~~~~~~~~~~~~~~~~~~~<1           6<------------------GTAT---------------TC<1           1<~~~~~~~~~~~~~~~~~~~~~~~~~~~~~~~~~~~~~~~<1      
