# Supplementary material for: AssemblyTron: flexible automation of DNA assembly with Opentrons OT-2 lab robots
Source: Synth Biol (Oxf). 2022 Dec 22;8(1):ysac032. doi: 10.1093/synbio/ysac032 (PMC9832943; doi:10.1093/synbio/ysac032)
Supplement: ysac032_Supp [file ysac032_supp.zip › suppl_data/File9_AssemblyTron_Vignette.pdf]

AssemblyTron

AssemblyTron is a package for automating DNA assembly with an Opentrons liquid handling robot. DNA-Assembly line implements combinatorial DNA assemblies based on designs created with j5 (Hillson, N.J., Rosengarten, R.D., and Keasling J.D. (2012) j5 DNA Assembly Design Automation Software. ACS Synthetic Biology 1 (1), 14-21. DOI: 10.1021/sb2000116), currently accessibe via: https://public-diva.jbei.org/.

This vignette is intended to guide a novice user through installation and use of AssemblyTron to build DNA constructs via Golden Gate assembly in the Opentrons OT-2 robot. We assume that the user has a folder/directory containing the results of a j5 combinatorial assembly design on the computer connected to their OT-2 robot. AssemblyTron then operates on the files within this folder to generate specific assembly instructions for the user and the OT-2 robot which are saved in this folder.

Technical documentation is provided in html format in the AssemblyTron/docs folder and hosted at: https://assemblytron.readthedocs.io/en/latest/AssemblyTron.html

Open terminal and install AssemblyTron with pip

In [1]:

```
! pip install AssemblyTron
```

```
Collecting AssemblyTron==0.0.5
  Using cached AssemblyTron-0.0.5-py3-none-any.whl (479 kB)
Requirement already satisfied: numpy in c:\users\opentrons\anaconda3\lib\site-packages
(from AssemblyTron==0.0.5) (1.20.1)
Requirement already satisfied: pandas in c:\users\opentrons\anaconda3\lib\site-packages
(from AssemblyTron==0.0.5) (1.2.4)
Collecting datetime
  Using cached DateTime-4.5-py2.py3-none-any.whl (52 kB)
Requirement already satisfied: pytz in c:\users\opentrons\anaconda3\lib\site-packages (f
rom datetime->AssemblyTron==0.0.5) (2021.1)
Requirement already satisfied: zope.interface in c:\users\opentrons\anaconda3\lib\site-p
ackages (from datetime->AssemblyTron==0.0.5) (5.3.0)
Requirement already satisfied: python-dateutil>=2.7.3 in c:\users\opentrons\anaconda3\li
b\site-packages (from pandas->AssemblyTron==0.0.5) (2.8.1)
Requirement already satisfied: six>=1.5 in c:\users\opentrons\anaconda3\lib\site-package
s (from python-dateutil>=2.7.3->pandas->AssemblyTron==0.0.5) (1.15.0)
Requirement already satisfied: setuptools in c:\users\opentrons\anaconda3\lib\site-packa
ges (from zope.interface->datetime->AssemblyTron==0.0.5) (52.0.0.post20210125)
Installing collected packages: datetime, AssemblyTron
Successfully installed AssemblyTron-0.0.5 datetime-4.5
```

Navigate to your local ~/site-packages directory to access AssemblyLine. Click on the DNA-AssemblyLine folder, and open the software directory.

In [2]:

```
! pip show AssemblyTron
```

```
Name: AssemblyTron
Version: 0.0.5
Summary: A package for automating DNA assembly with an Opentrons liquid handling robot
```

```
Home-page: https://github.com/PlantSynBioLab/opentrons
Author: John Bryant
Author-email: jbryant2@vt.edu
License: Apache License 2.0
Location: c:\users\opentrons\anaconda3\lib\site-packages
Requires: numpy, datetime, pandas
Required-by:
```

In [4]:
```
! cd c:\users\opentrons\anaconda3\lib\site-packages
```

Setup AssemblyTron

Run the pathswriter module in order to record your working directory and enable protocol setup scripts to navigate accurately. The pathswriter.py must be run in the AssemblyLine working directory so that the correct path is generated, and the paths.csv file is saved in the proper location.

In [8]:
```
! python -m AssemblyTron.pathswriter
```

Since AssemblyTron has an R-script integrated, the pathswriter module will prompt you to select the folder where 'Rsript.exe' file is located with a `tkinter` file explorer window. This can be found in the folder where R was downloaded, and the AssemblyTron prompt offers an example of where to look. Of course this will vary between machines.

Rscript.exe is often in a hidden folder, so be sure to show hidden files and folders on the file explorer.

Select confirm to create the paths.csv file.

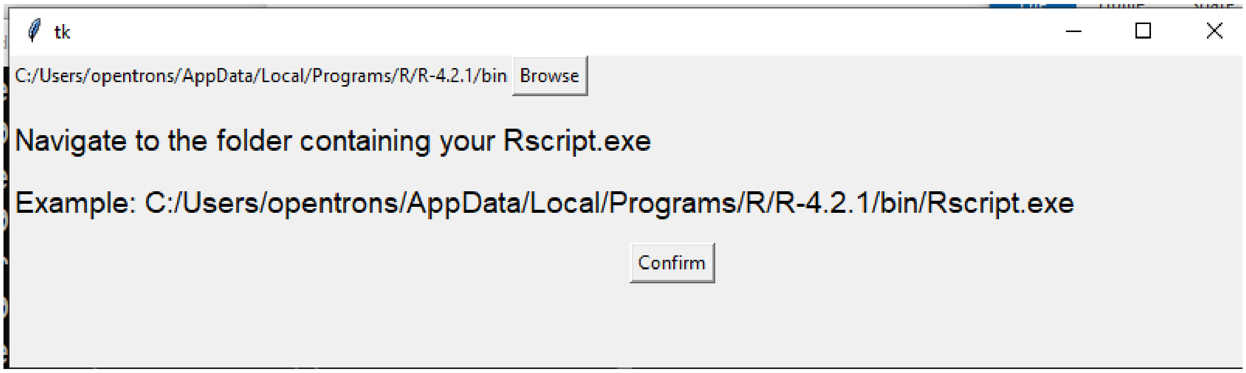

Once paths.csv has been created, there are five options for protocols.

The first option is a homology dependent assembly with up to 24 combined primers and templates.

In [ ]:
```
! python -m AssemblyTron.Cloning.Setup_seppcr_gradient_24
```

The second option is a homology dependent assembly with up to 96 combined primers and templates.

In [ ]:
```
! python -m AssemblyTron.Cloning.Setup_seppcr_gradient_96
```

The third option is a Golden Gate assembly with a destination plasmid with restriction sites. The destination plasmid is typically the backbone fragment and does not require PCR amplification.

In [ ]:
```
! python -m AssemblyTron.Golden_Gate.Setup_digests_gradient
```

The fourth option is a Golden Gate assembly with 24 combined primers and templates.

In [ ]:
```
! python -m AssemblyTron.Golden_Gate.Setup_nodigests_seppcr_gradient_24
```

The fifth option is a Golden Gate assembly with 96 combined primers and templates.

In [ ]:
```
! python -m AssemblyTron.Golden_Gate.Setup_nodigests_seppcr_gradient_96
```

This vignette will go through the workflow of option five, which is a Golden Gate assembly with 96 possible primers and templates. After running the module, a `tkinter` file explorer window will pop up. This is for the user to navigate to the j5 combinatorial assembly design folder saved on the computer attached to the OT-2.

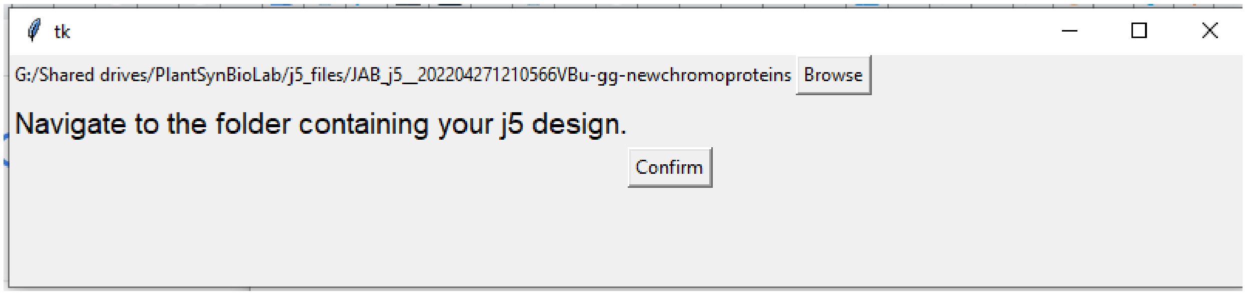

Once the folder containing the assembly design file is selected, select confirm to proceed.

AssemblyTron will run the R script for parsing the combinatorial design file at this point. No additonal actions are required.

A reagents setup text file window will automatically appear. This specifies where to place primers, templates, labware, etc. For the 96 well dilution script, it specifies the deck slot and tube rack position where the template belongs as well, since the deck has up to four slots with 24-tube racks for stock primers and templates. The absolute path, date, and time are also included at the top of this file.

We will return to this file when we are ready to insert stock primers and templates into the OT-2 deck. Close for now, and follow the instructuion when finished with the setup script.

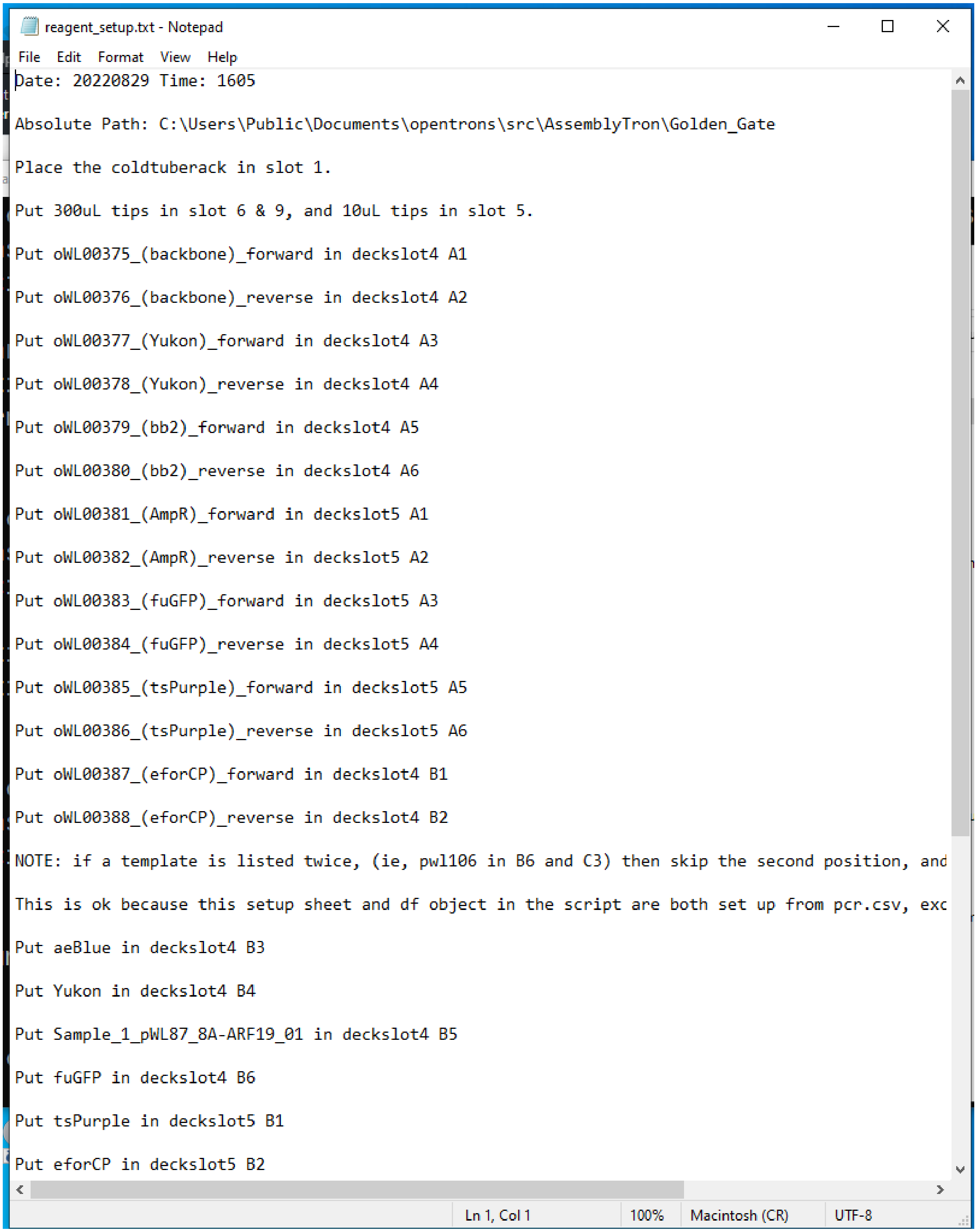

reagent_setup.txt - Notepad

File Edit Format View Help

```
Date: 20220829 Time: 1605

Absolute Path: C:\Users\Public\Documents\opentrons\src\AssemblyTron\Golden_Gate

Place the coldtuberack in slot 1.

Put 300uL tips in slot 6 & 9, and 10uL tips in slot 5.

Put oWL00375_(backbone)_forward in deckslot4 A1

Put oWL00376_(backbone)_reverse in deckslot4 A2

Put oWL00377_(Yukon)_forward in deckslot4 A3

Put oWL00378_(Yukon)_reverse in deckslot4 A4

Put oWL00379_(bb2)_forward in deckslot4 A5

Put oWL00380_(bb2)_reverse in deckslot4 A6

Put oWL00381_(AmpR)_forward in deckslot5 A1

Put oWL00382_(AmpR)_reverse in deckslot5 A2

Put oWL00383_(fuGFP)_forward in deckslot5 A3

Put oWL00384_(fuGFP)_reverse in deckslot5 A4

Put oWL00385_(tsPurple)_forward in deckslot5 A5

Put oWL00386_(tsPurple)_reverse in deckslot5 A6

Put oWL00387_(eforCP)_forward in deckslot4 B1

Put oWL00388_(eforCP)_reverse in deckslot4 B2

NOTE: if a template is listed twice, (ie, pwl106 in B6 and C3) then skip the second position, and

This is ok because this setup sheet and df object in the script are both set up from pcr.csv, exc

Put aeBlue in deckslot4 B3

Put Yukon in deckslot4 B4

Put Sample_1_pWL87_8A-ARF19_01 in deckslot4 B5

Put fuGFP in deckslot4 B6

Put tsPurple in deckslot5 B1

Put eforCP in deckslot5 B2
```

Ln 1, Col 1 | 100% | Macintosh (CR) | UTF-8

Specifying stock concentrations and other parameters

Next, another window will appear for specifying parameters of the assembly. All of the volume and concentration reaction parameters are autofilled with default values, however the user can change these as necessary. This window also displays the DNA templates that will be used for the assembly, allowing the user to retrieve stocks of these and input their concentrations in ng/μL, at this time. Finally, there are two slots for extra parameters in case the user wants to modify the source code,

this can be done without having to modify this user interface. These extra parameters are 0 by default.

Once the parameter are set, click confirm.

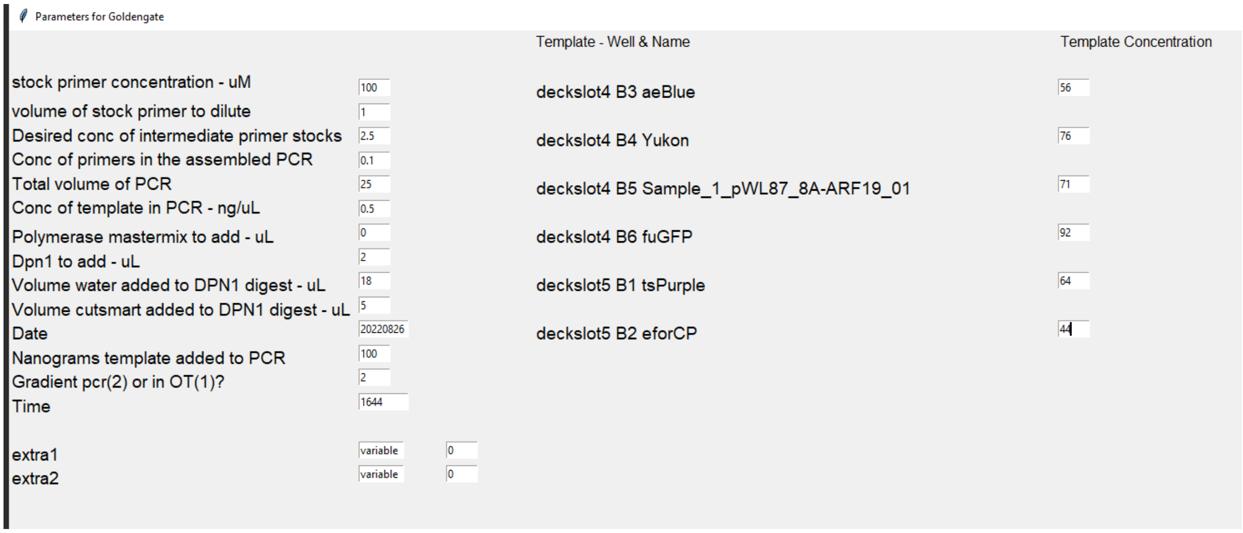

Next, a new window will appear where the user can choose which steps of the assembly to perform. Often, it will be necessary to skip to later steps of the workflow or only perform the first steps. So this tool gives the user the ability to only run the parts of the script that they need, again without having to modify source code. Once the desired protocol steps are selected, click confirm.

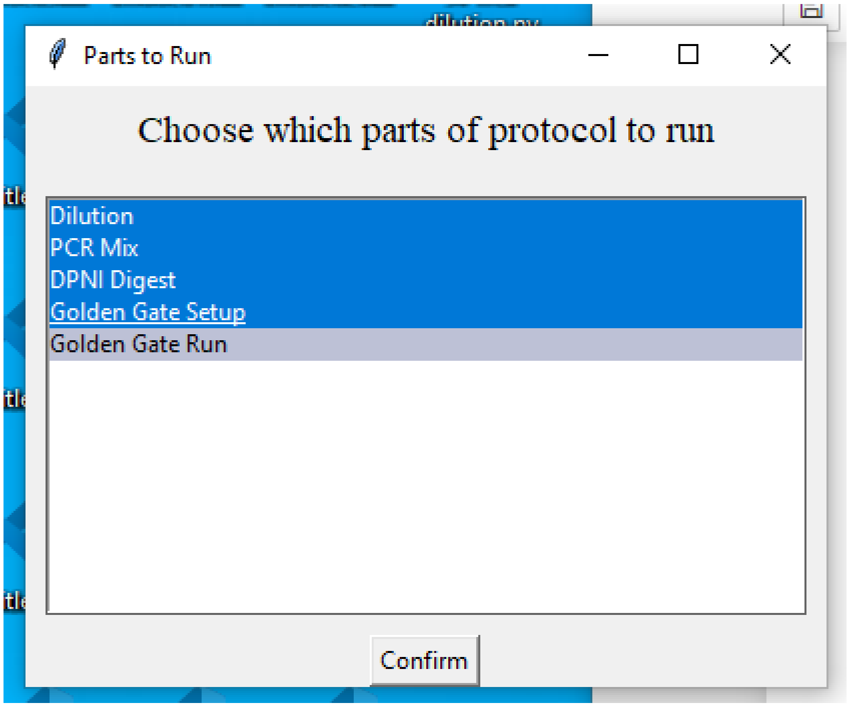

Next, a reaction setup text file will pop up to specify the tube configuration that needs to be used on the thermocycler. This is similar to the reagent_setup text file. Both are saved in the Golden Gate folder with the current date.

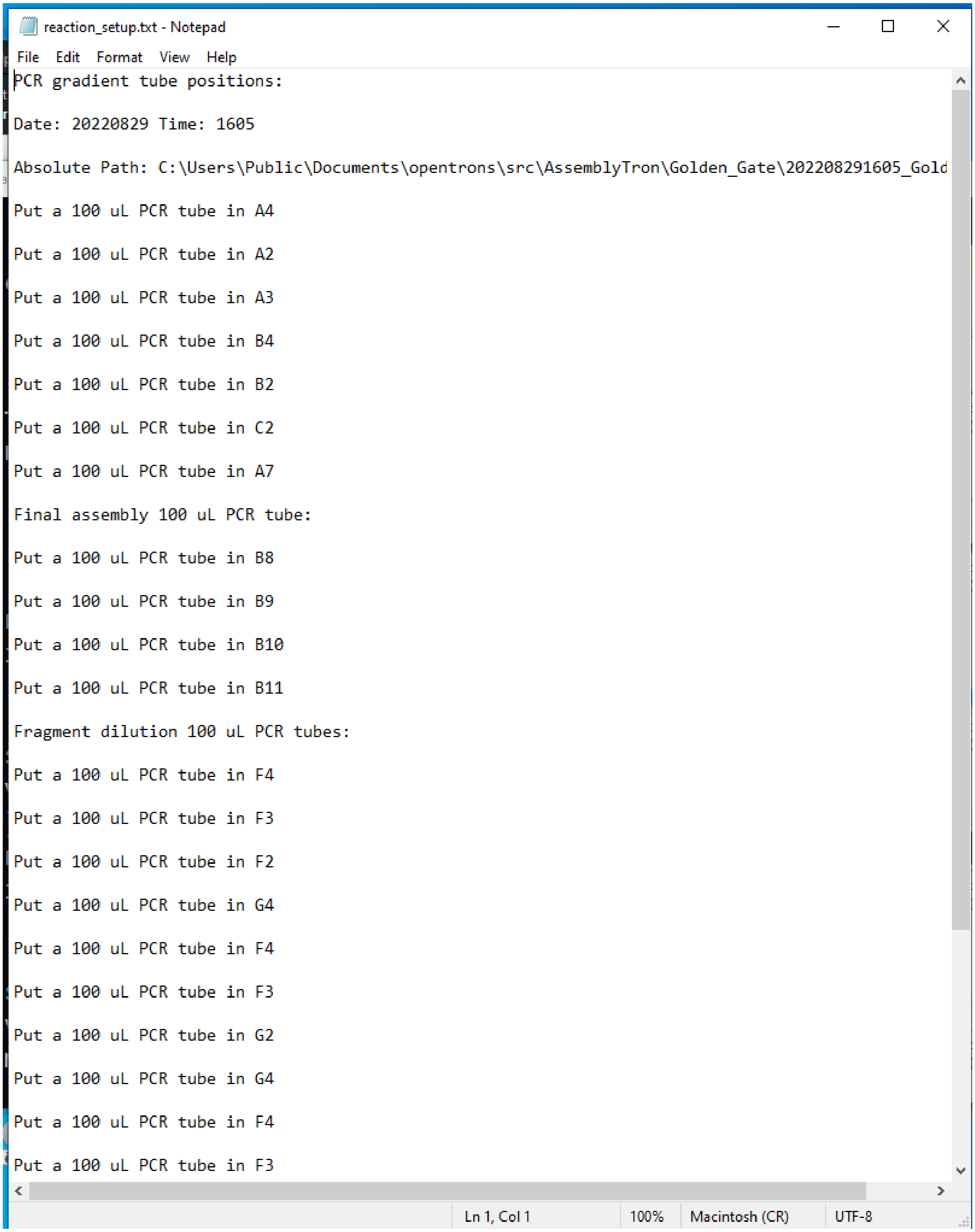

reaction_setup.txt - Notepad

File Edit Format View Help

```
PCR gradient tube positions:

Date: 20220829 Time: 1605

Absolute Path: C:\Users\Public\Documents\opentrons\src\AssemblyTron\Golden_Gate\202208291605_Gold

Put a 100 uL PCR tube in A4

Put a 100 uL PCR tube in A2

Put a 100 uL PCR tube in A3

Put a 100 uL PCR tube in B4

Put a 100 uL PCR tube in B2

Put a 100 uL PCR tube in C2

Put a 100 uL PCR tube in A7

Final assembly 100 uL PCR tube:

Put a 100 uL PCR tube in B8

Put a 100 uL PCR tube in B9

Put a 100 uL PCR tube in B10

Put a 100 uL PCR tube in B11

Fragment dilution 100 uL PCR tubes:

Put a 100 uL PCR tube in F4

Put a 100 uL PCR tube in F3

Put a 100 uL PCR tube in F2

Put a 100 uL PCR tube in G4

Put a 100 uL PCR tube in F4

Put a 100 uL PCR tube in F3

Put a 100 uL PCR tube in G2

Put a 100 uL PCR tube in G4

Put a 100 uL PCR tube in F4

Put a 100 uL PCR tube in F3
```

Ln 1, Col 1 | 100% | Macintosh (CR) | UTF-8

Close this for now, and re-open after completing these setup steps. The protocol is now complete and ready to be loaded onto the OT-2.

Move the protocols to the OT-2

Run the file transfer script on bash to move CSV files onto the Opentrons OT-2's internal computer for the run. The paths within this script will need to be modified to match the AssemblyLine working

directory (found using pip show DNA-AssemblyLine, assuming you have installed AssemblyTron using pip). This script is called Copy_Cloning.bat and is in the src directory of AssemblyTron.

It is necessary to coordinate with your computer systems administrator and Opentrons tech support to set a a wired static ip address for your OT-2. Opentrons will provide a protocol script for setting this up, and keys will need to be set up for automated ssh access to the robot.

Do not set the IP address as either your computer or OT-2, and do not change the subnet mask. The static IP address needs to be reachable from the computer with the Opentrons App (aka selected from the proper subnet). The network needs to be specially set up to account for the OT-2 having the static IP address. Otherwise, the OT-2 could conflict with another device on the network.

There is a python script for setting up the wired static IP address in the ~/src/AssemblyTron/ directory called static_wired_IP_address.py (this script was obtained from Opentrons).

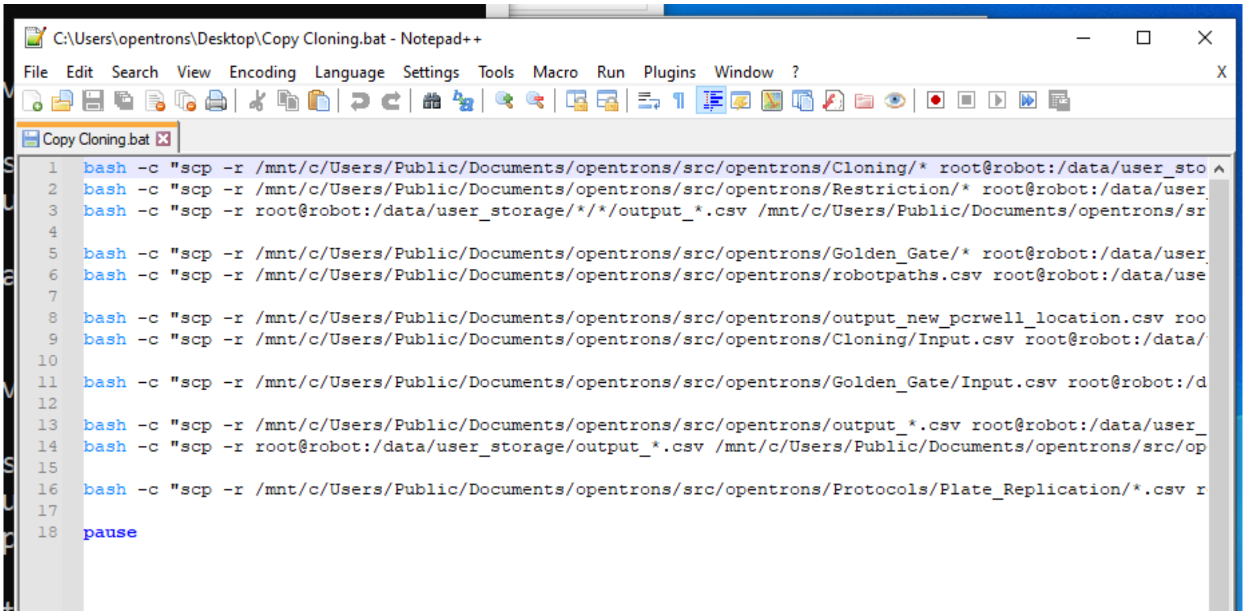

Run the protocols on the OT-2

Power on the OT-2 and start the Run app. Select the protocol tab on the left side of the window. Click on choose file. Navigate to the AssemblyTron working directory (found using pip show AssemblyTron), then navigate to the Golden_Gate/date+time_GoldenGate directory you just generated.

First, run 1_Update_Input.py to ensure the robot calls the correct Input.csv file. Failure to complete this step could result in errors and failure. This file is run on the command line witht the command:

In [ ]:
```
! python -m AssemblyTron.Golden_Gate.{insert dated folder}.1_Update_Input.py
```

or if your current working directory is the new directory you just generated:

In [ ]:
```
!python -m 1_Update_Input.py
```

Next, run the protocol 2_dilution_96.py. Select this file and drag to protocol upload are on the OT-2 run app, or open directly through the run app.

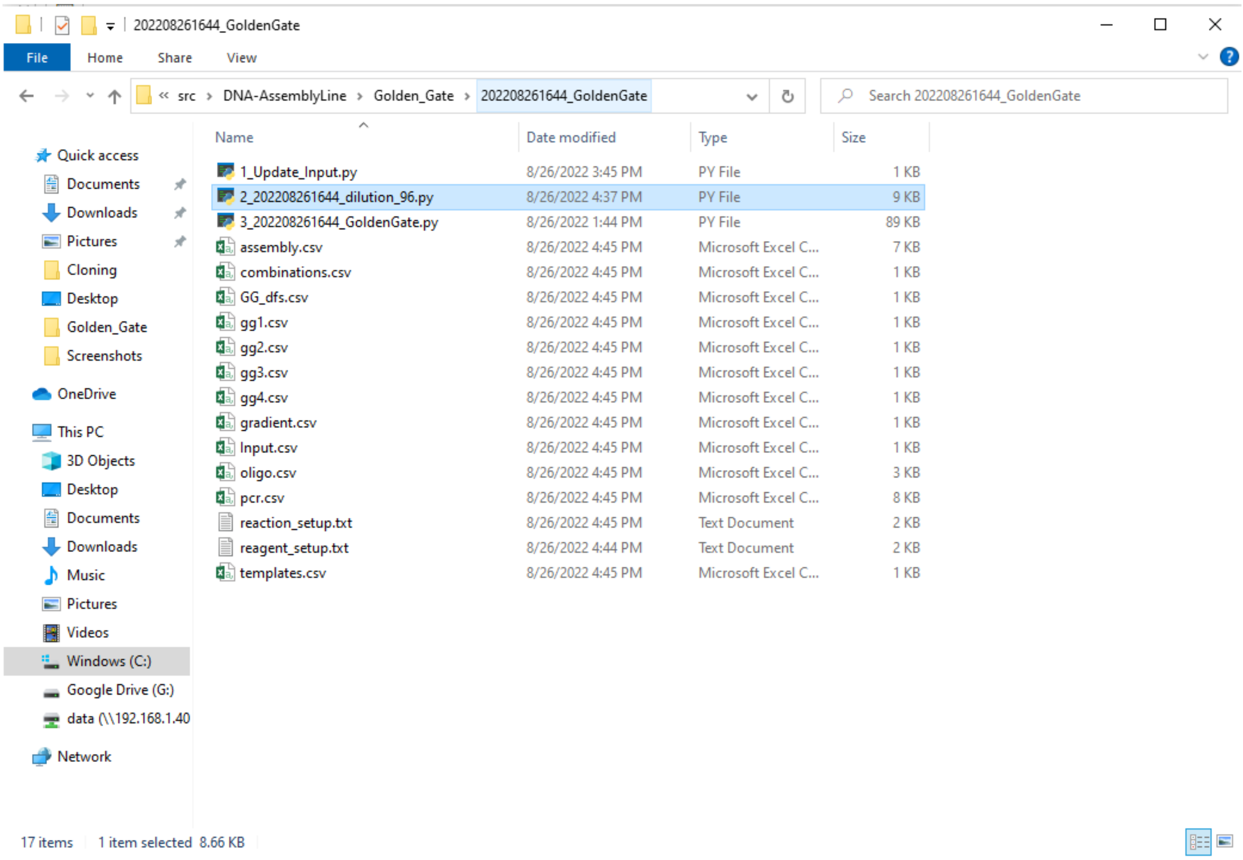

The opentrons app will then read this protocol script and show the deck setup for the initial primer and template dilution step.

Now open the reagent_setup.txt file and follow the slot and tube position designations to place primer and template stock tubes in the correct locations.

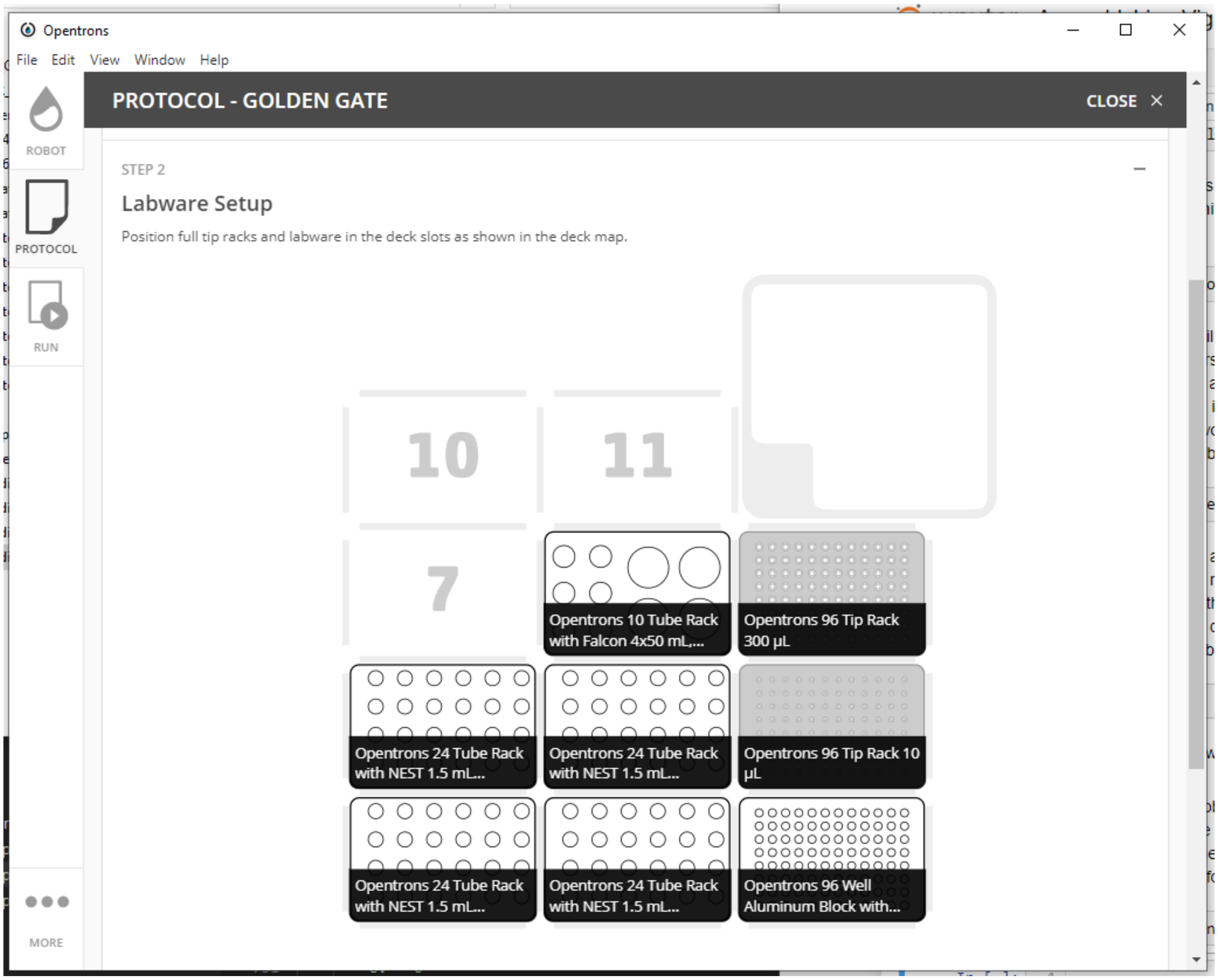

Review the protocol steps and run the dilution script.

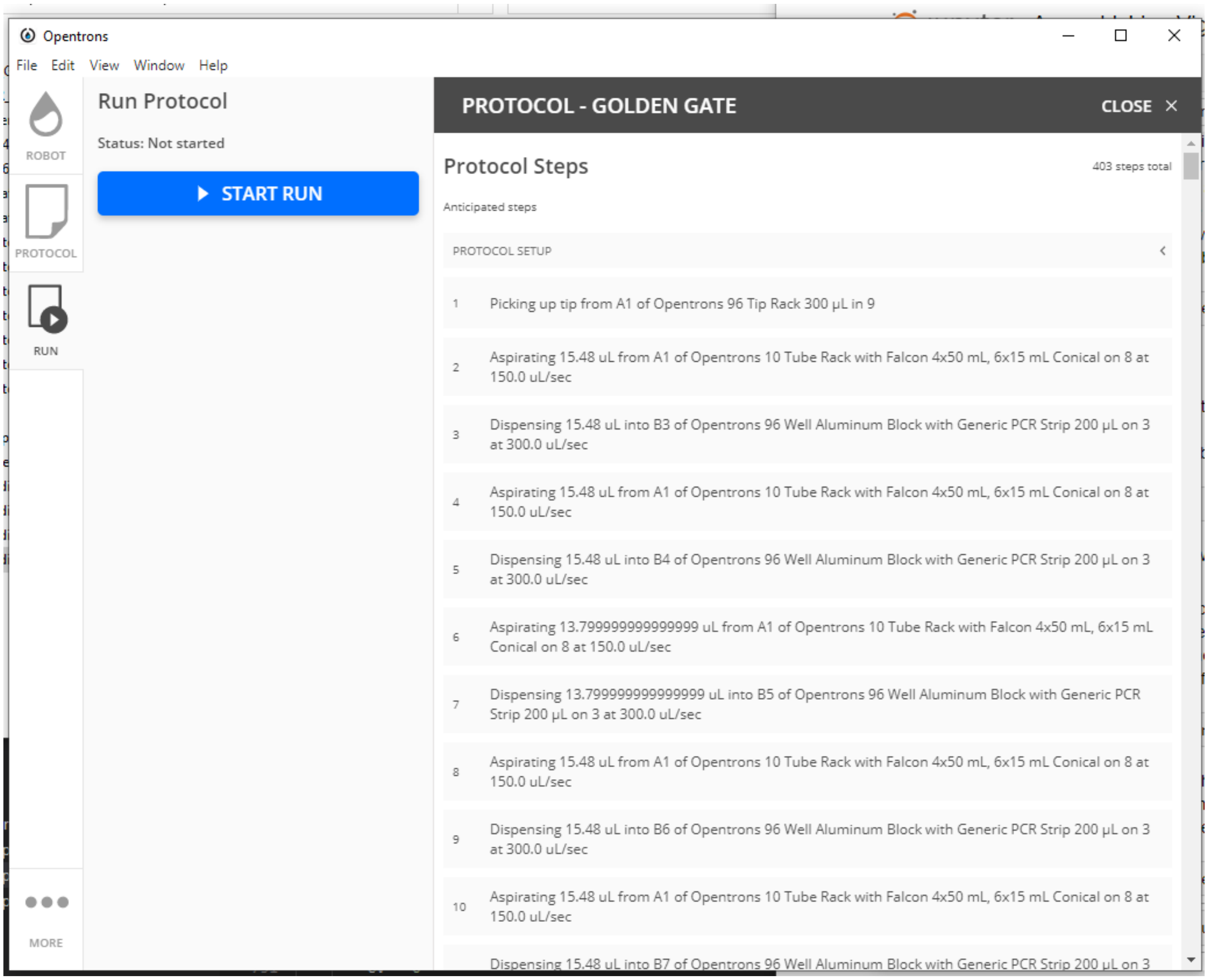

At the conclusion of this run, return to the protocol tab and close the dilution script. Restart the OT-2 to avoid system freezing or crashing. Select CHOOSE FILE... again and open the 2_date+time_GoldenGate.py script. Follow the layout shown on the OT-2 for rearranging the OT-2 deck, set up reagents with reagent_setup.txt, set up PCR tubes with reaction_setup.txt, and review the protocol steps, then select START RUN.

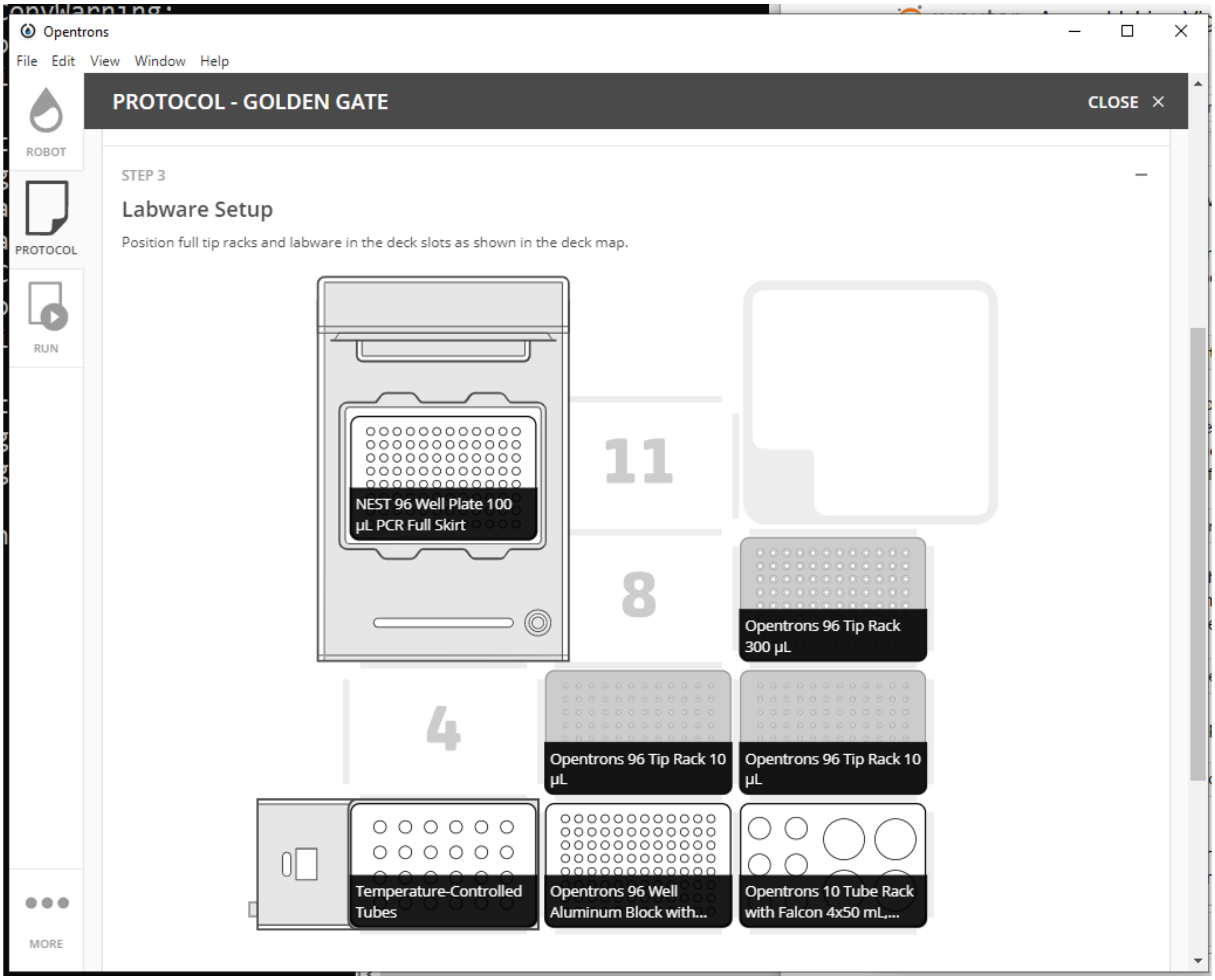

Follow prompts throughout the protocol run. For example, after PCRs are mixed, the protocol pauses and the user is prompted to move PCR strips to a gradient thermocycler. Optimized annealing and extension parameter are provided to set up the gradient thermocycler.

PAUSE PROTOCOL

385 move to gradient thermocycler. set gradiet to be between 61.16767806650506 and 70.06570375337782. Extension time should be 1 31.74 Name: Length, dtype: float64 seconds. Follow normal parameters for everything else. A1 is cool, A8 is hot.

The user will also be prompted to refill tipracks, remove enzymes, etc.

PAUSE PROTOCOL

587 REFILL TIP RACKS, and wait until its time to dispense the product

At the conclusion of the run, retrieve assemblies for transformation. To power down, return all reagents to the freezer, power off the modules and OT-2, and clean the OT-2 deck.
